# A general mathematical method for predicting spatio-temporal correlations emerging from agent-based models

**DOI:** 10.1098/rsif.2020.0655

**Published:** 2020-10-28

**Authors:** Otso Ovaskainen, Panu Somervuo, Dmitri Finkelshtein

**Affiliations:** 1 Organismal and Evolutionary Biology Research Programme, University of Helsinki, P.O. Box 65, Helsinki FI-00014, Finland; 2 Centre for Biodiversity Dynamics, Department of Biology, Norwegian University of Science and Technology, N-7491 Trondheim, Norway; 3 Department of Mathematics, Swansea University, Fabian Way, Swansea SA1 8EN, UK

**Keywords:** agent-based model, marked point process, Markov evolution, theoretical ecology, spatio-temporal correlation

## Abstract

Agent-based models are used to study complex phenomena in many fields of science. While simulating agent-based models is often straightforward, predicting their behaviour mathematically has remained a key challenge. Recently developed mathematical methods allow the prediction of the emerging spatial patterns for a general class of agent-based models, whereas the prediction of spatio-temporal pattern has been thus far achieved only for special cases. We present a general and mathematically rigorous methodology that allows deriving the spatio-temporal correlation structure for a general class of individual-based models. To do so, we define an auxiliary model, in which each agent type of the primary model expands to three types, called the original, the past and the new agents. In this way, the auxiliary model keeps track of both the initial and current state of the primary model, and hence the spatio-temporal correlations of the primary model can be derived from the spatial correlations of the auxiliary model. We illustrate the agreement between analytical predictions and agent-based simulations using two example models from theoretical ecology. In particular, we show that the methodology is able to correctly predict the dynamical behaviour of a host–parasite model that shows spatially localized oscillations.

## 1. Introduction

In many fields of science, ranging from physics to biology to humanities, it is of major interest to understand how the microscopic interactions among agents lead to emerging patterns at the macroscopic level. Often the interactions are spatially localized, meaning that an interaction between agents is more likely to take place if they are located close to each other in space. Localized interactions lead generally to emerging patterns where agents are non-randomly distributed with respect to each other over space and time. Such emerging patterns can be characterized with the help of spatial and spatio-temporal correlation structures. Spatial correlation structures concern patterns that can be observed at a single time, describing e.g. how much more or less likely it is to observe an agent in a given location, given that another agent is observed in a nearby location. Spatio-temporal correlation structures in turn concern comparisons of spatial patterns observed at multiple times, describing e.g. how much more or less likely it is to observe an agent in a given location at a given time, given that another agent was observed in a nearby location some time ago.

Agent-based models provide a generic tool for studying complex dynamical phenomena in many fields of science [1–4]. They are typically straightforward to simulate, and emerging properties of the dynamics, such as spatial or spatio-temporal correlations, are routinely measured from their output. However, it has remained difficult to develop generic tools that could be used to supplement simulation results with analytical insights. Recently developed mathematical methods are filling this gap, as they allow mathematically rigorous predictions of the emerging spatial patterns for a very general class of agent-based models [5,6]. However, at the moment, there are no general mathematical methods that would allow one to predict the spatio-temporal correlation structures generated by the dynamics of agent-based models. This is a major caveat, because understanding the causes and consequences of spatio-temporal correlation structures is central to the study of many fields of research.

As one example of the relevance of spatio-temporal correlation structures, a central topic in ecology is that of spatial synchrony in spatial population dynamics, meaning that spatially nearby populations tend to fluctuate over time in synchrony with each other. Ecologists have long been interested in the mechanisms behind such synchrony [7], including why and how patterns of such synchrony vary over geographical regions [8]. Theoretical works based on simulations [9] and mathematical analyses [10,11] have shown that spatial synchrony in population dynamics can be generated both by dispersal and by environmental stochasticity, the relative roles between these two depending on the strength of density regulation [10]. Such theoretical insights may be compared to empirical data, e.g. a case study on forest insect outbreaks suggesting that spatially correlated environmental stochasticity (the so-called Moran effect) is more important than dispersal in generating spatial synchrony in population dynamics [12]. Spatio-temporal correlation structures can have major consequences to large-scale dynamics, such as synchrony in population dynamics increasing the risk of global extinction [13]. As another example of the relevance of spatio-temporal correlation structures, the prediction of an upcoming earthquake mainshock can be based on the distribution of smaller events in the nearby area [14]. Based on the spatio-temporal correlation structure of the Burridge–Knopoff model [15], preceding the mainshock, the frequency of smaller events is gradually enhanced, whereas it is dramatically suppressed in the close vicinity just before the upcoming mainshock.

Understanding the emergence of spatio-temporal correlation structures can be especially important during transient dynamics, as e.g. the fate of an evolving population can depend critically on small chance events earlier in time. As one example, empirically derived spatio-temporal correlation functions describing plant community dynamics were used to explore how interactions among and within species influence the pathways of primary successional dynamics [16]. As another example, spatio-temporal correlation structures emerging from agent-based models were used to understand within-host immune response during the early spread of flu infection [17] as well as the early phase of viral propagation of HIV [18]. As a third example, simulations of an agent-based model were used to characterize the spatial synchrony of an influenza epidemic spreading over the continental scale of Australia [19].

Spatio-temporal correlation structures emerging from stochastic and spatial models have thus far been derived mainly from simulation approaches, whereas mathematical results are available for special cases only. As one example of a mathematical result, the exact two-point spatio-temporal correlation function for the Takayusa model of mass aggregation has been derived analytically [20]. The Takayusa lattice model describes a system in which masses diffuse, coalesce upon contact, and adsorb mass from outside, and it can be considered as a simple toy model for earthquake dynamics [20]. As another example of a mathematical result, [21] derived the spatio-temporal correlation structure of a lattice-based self-organized critical model of punctuated equilibrium, with results mimicking patterns observed in the fossil record and in earthquake data. As a third example of a mathematical result, the spatial scale of population synchrony was shown to depend additively on the spatial scales of environmental stochasticity and dispersal [10]. This result was not derived from an agent-based model but a stochastic model of a spatially continuous population, and it relied on the assumption of small noise, the consequences of which assumption were examined later [22]. In all three cases, the availability of mathematical results on spatio-temporal correlation structures has allowed for more general insights than would have been possible if relying on a simulation-based approach only.

As discussed above, being able to predict spatio-temporal correlation structures emerging from agent-based models would be highly valuable, but thus far this has been possible only for special cases. Here, we overcome this caveat by extending the recently developed approach for predicting spatial patterns [5,6] to cover also the prediction of spatio-temporal patterns. We note that the mathematically rigorous methods of [5,6] were preceded by heuristic methods [23], in which methods were used to derive not only spatial but also spatio-temporal correlation structures, e.g. of a metapopulation model [24] and of a host–parasite model [23]. However, in addition to these results not being mathematically rigorous, they required tedious model-specific derivations. Here we provide a step-change by introducing a systematic and mathematically rigorous procedure for predicting the spatio-temporal correlation structure of a very general class of agent-based models.

## 2. The mathematical framework of reactant–catalyst–product models

We consider the modelling framework of spatio-temporal point processes, also called Markov evolutions in the space of locally finite configurations [5]. In particular, we consider the general class of models suggested by [6] that can be generated with the help of reactants (R), catalysts (C) and products (P), called henceforth RCP models. Reactants are any agents that disappear in a reaction, products are any agents that appear in a reaction, whereas catalysts are any agents that remain unchanged but they modify a rate of a reaction. RCP models apply to a broad class of situations, such as models of population ecology [5,6,25], metapopulation ecology [24,26], community ecology [23,27,28], pathogenesis [29], evolutionary ecology [6,30,31] and movement ecology [6,32].

The general theory and mathematical framework of RCP models is discussed in detail in [5,6]. Briefly, the state of an RCP model at any time *t* is a point configuration $\gamma_{t}$ in the *d*-dimensional Euclidean space $R^{d}$, where typically *d* = 1,2,3. We consider marked configurations, so that $\gamma_{t}$ contains not only information on the locations of the agents, but also their types, so that the process can contain e.g. multiple species. We assume that the number of individuals within any finite region is finite, so that mathematically, we consider the space of locally finite configurations, $\Gamma :=\{\gamma \subset R^{d},|\gamma \cap \Lambda |<\infty \text{ for any bounded Lambda }\subset R^{d}\}$, where, if *A* is a discrete set of points, then |A| stands for the number of points in *A*. A probability measure $\mu_{t}$ on Γ describes the state of the system at time *t*. Informally, the measure $\mu_{t}$ describes how likely the system is to be in a given configuration at time *t*, given that it starts from an initial state described by the measure $\mu_{0}$ at time 0. The evolution of the measure is defined with the help of observables *F*, i.e. functions on Γ which typically take real values. Given a configuration γ, the observable F(γ) is a numerical quantity that characterizes some property of the configuration, such as the number of agents in a particular subdomain. A pairing between an observable *F* and a measure μ is defined as $\langle F,\mu \rangle :=\int_{\Gamma }F(\gamma )\text{d}\mu (\gamma ),\;$ and this pairing gives information about the state of the system, i.e. the measure μ. The dynamics of the configuration over time is described by a linear operator *L* acting on the observables, which operator defines the set of model components, i.e. the population events that can take place, as well as how the rates at which the reactions take place depend on the current configuration of the agents. The evolution of states is then defined through the differential equation $\left(\text{d/d}t)\langle F,\mu_{t}\rangle =\langle LF,\mu_{t}\right\rangle$. For a more detailed discussion of the mathematical framework, see [5,6].

As an example used to derive our argument, we consider the spatial and stochastic logistic model [5], henceforth, the SSLM. This model includes three processes, which we call density-independent death, density-dependent death and reproduction. The rate at which density-independent death takes place is a constant *m*. In the terminology of RCP models, the agent that dies is a reactant, as it disappears in the reaction. The rate of density-dependent death for an agent located at *x* is $\sum_{y\in \gamma_{t}\setminus x}a^{-}(x-y)$, where $\;a^{-}(\cdot )$ is the competition kernel, which defines how the rate by which an agent at *y* increases the death rate of another agent located at *x* depends on the difference x−y of their spatial coordinates, typically (but not necessarily) on the distance |x−y|. In the terminology of RCP models, the agent at *x* is a reactant as it disappears during the reaction, whereas the agent at *y* is a catalyst as it remains unchanged during the reaction. Finally, the per-unit-area rate of reproduction at location *y* is $\sum_{x\in \gamma_{t}}a^{+}(x-y),$ where the reproduction kernel $a^{+}(x-y)$ defines the per-unit-area rate by which the mother at *x* produces offspring to the vicinity of *y*. In the terminology of RCP models, the mother is a catalyst, as it remains unchanged when the process takes places, whereas the offspring is a product, as it appears in the reaction. While we have described here the model verbally, we refer to [5,6] and the electronic supplementary material on how RCP models can be defined mathematically in terms of time evolution of a probability distribution of point configurations.

The utility of the RCP framework is the availability of mathematical tools that make it possible to predict many properties of the dynamics generated by the models, even if the models are spatial, stochastic and can include nonlinear behaviour. Most importantly, it is possible to write down an exact equation for the time evolution of spatial correlation functions. The one-point correlation function $k_{t}^{\left(1\right)}(x)$ measures expected agent density at location *x*, so that the expected number of agents within an area Λ can be computed as

2.1 $$E(\left|\gamma_{t}\cap \Lambda \right|)=\int_{\Lambda }k_{t}^{\left(1\right)}(x)\text{d}x.$$

The two-point correlation function $k_{t}^{\left(2\right)}(x_{1},x_{2})$ measures the expected product for the numbers of agents that are found within the areas $\Lambda_{1}$ and $\Lambda_{2}$ by

2.2 $$E(\left|\gamma_{t}\cap \Lambda_{1}||\gamma_{t}\cap \Lambda_{2}\right|)=\int_{\Lambda_{1}}\int_{\Lambda_{2}}k_{t}^{\left(2\right)}(x_{1},x_{2})\text{d}x_{2}\text{d}x_{1}+\int_{\Lambda_{1}\cap \Lambda_{2}}k_{t}^{\left(1\right)}(x)\text{d}x.$$

We note that the second term appears because if the areas $\Lambda_{1}$ and $\Lambda_{2}$ are not disjoint, as then the expectation $E(\left|\gamma_{t}\cap \Lambda_{1}||\gamma_{t}\cap \Lambda_{2}\right|)$ counts also the pair of the agent and itself, and that the density of such self-pairs is given by the density $k_{t}^{\left(1\right)}(x)$ of the agents themselves. Similarly, we may define three-point or four-point correlation functions, or more generally the correlation functions of any order. Correlation functions of all possible orders are collected into the vector $k_{t}(\eta )$, where $\eta \in \Gamma_{0}$, and $\Gamma_{0}$ is the set of all finite subsets of $R^{d}$.

The focus of the present paper is to develop methods for predicting spatio-temporal correlation functions that emerge from the dynamics of any RCP model. While the methodology extends to an arbitrary number of time points, we restrict the treatment here to two time points, denoted by *t* and t+Δt, where Δt≥0. With the spatio-temporal correlation function $k_{t,\Delta t}(x_{1},x_{2})$, we wish to ‘look' at the system at location *x*_1_ at time *t*, and at location *x*_2_ at time t+Δt, thus having a time lag Δt between the two observations. The spatio-temporal correlation function relates to the expected product between the number of agents that are in the area $\Lambda_{1}$ at time *t*, and the number of agents that are in the area $\Lambda_{2}$ at time t+Δt:

2.3 $$E(\left|\gamma_{t}\cap \Lambda_{1}||\gamma_{t+\Delta t}\cap \Lambda_{2}\right|)=\int_{\Lambda_{1}}\int_{\Lambda_{2}}k_{t,\Delta t}(x_{1},x_{2})\text{d}x_{2}\text{d}x_{1}+\int_{\Lambda_{1}\cap \Lambda_{2}}k_{t,\Delta t}(x)\text{d}x.$$

We note that as with equation (2.3), we have included here the term $k_{t,\Delta t}(x)$ that arises due to a possibility of a self-pair, if an agent that was present at location *x* at time *t* is still there at time t+Δt.

Earlier work [6] shows that for any RCP model, the vector of correlation functions of all orders follows a differential equation that can be written as

2.4 $$\frac{\text{d}}{\text{d}t}k_{t}(\eta )=(L^{\Delta }k_{t})(\eta ),$$

where the linear operator $L^{\Delta }$ can be derived for any RCP model using the systematic and mathematically rigorous procedure of [6]. For example, the dynamical equation of the one-point correlation function in the SSLM is given by

2.5 $$\frac{\text{d}}{\text{d}t}k_{t}^{\left(1\right)}(x)=-mk_{t}^{\left(1\right)}(x)-\int_{R^{d}}a^{-}(x-y)k_{t}^{\left(2\right)}(x,y)\text{d}y+\int_{R^{d}}a^{+}(x-y)k_{t}^{\left(1\right)}(y)\text{d}y.$$

The general expression for the operator $L^{\Delta }$ describes the dynamics simultaneously for all orders of correlation functions and is given for the SSLM in [5].

While the mathematical methods for deriving the dynamical equations of spatial correlation functions are well developed for RCP models [6], a corresponding theory for spatio-temporal correlation functions has been lacking. Thus, the open question that we solve here is how to obtain an expression for the spatio-temporal correlation functions $k_{t,\Delta t}(x_{1},x_{2})$.

## 3. The auxiliary model: original, new and past agents

**Figure 1.**
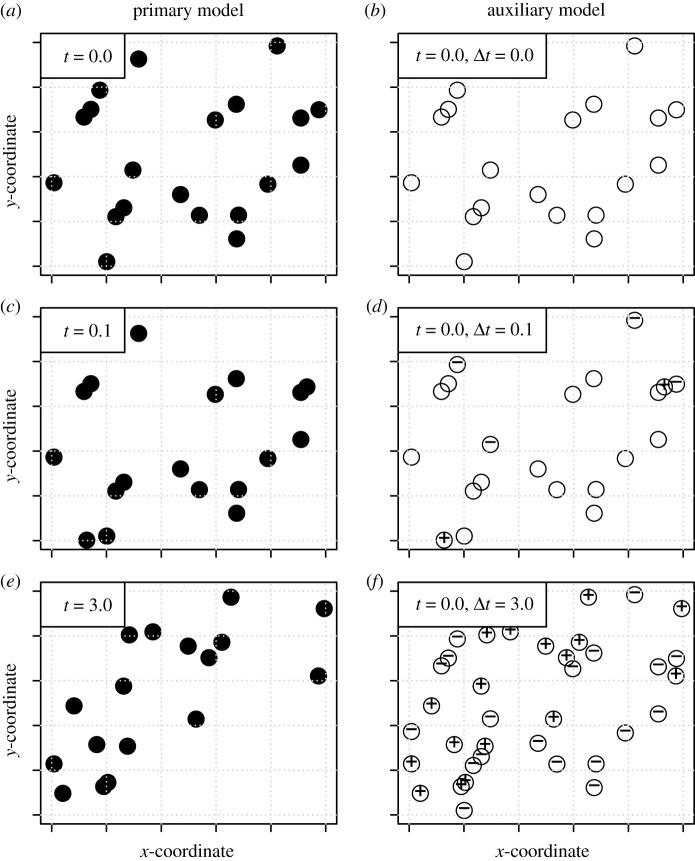
An illustration of the relationship between an primary model and the corresponding auxiliary model. The primary model is shown in (*a,c,e*), and it consists of a single entity type, shown by the filled circles. The auxiliary model is shown in (*b,d,f*), and it consists of three entity types: original agents (empty circles), past agents (circles with minus sign) that were present initially but that are not present currently, and new agents (circles with plus sign) that were not present initially but are present currently. For any time lag Δt, the initial configuration of the primary model (*a*) can be reconstructed from the auxiliary model as the union of the original and past agents, whereas the current configuration of the primary model (e.g. (*e*)) can be reconstructed from the auxiliary model (e.g. panel (*f*)) as the union of the original and new agents.

The main idea behind our solution of how to obtain spatio-temporal correlation functions is illustrated in figure 1. Panels *a*,*c* and *e* illustrate the dynamics of a particular parameterization of the SSLM on $R^{2}$, the locations of the agents shown for a small subset of the simulation domain. The snapshots shown in *a*, *c* and *e* illustrate the dynamics at time points t=0,t=0.1 and *t* = 3.0, respectively. To address spatio-temporal correlations, we consider an auxiliary model, the dynamics of which are illustrated in *b*, *d* and *f* of figure 1. In the auxiliary process, the time *t* of the primary model is kept fixed, here *t* = 0. The dynamic time variable of the auxiliary model is Δt, which is the time since the auxiliary process was initiated. The auxiliary model involves three kinds of agents. The first set of agents, called the original agents (or the *O* agents), are those that were present initially (at time *t* when Δt=0) and that are still present at the current time Δt. The new agents (or the + agents) are those agents that were not present initially but are present at the current time (when Δt>0). The past agents (or the - agents) are those that were present initially but that are not present anymore (at Δt>0). We denote by $\gamma_{\Delta t}^{O}$ the set of original agents, by $\gamma_{\Delta t}^{+}$ the set of new agents and by $\gamma_{\Delta t}^{-}$ the set of past agents. Initially (at Δt=0), the auxiliary model consists solely of the original agents, whose distribution is thus identical to the distribution of the agents of the primary model (figure 1*a,b*) so that $\gamma_{\Delta t=0}^{O}=\gamma_{t}$ whereas $\gamma_{\Delta t}^{+}$ and $\gamma_{\Delta t}^{-}$ are empty. At a later time, the state of the auxiliary model will involve a mixture of the original, new and past agents (figure 1*d*). After a long enough time, the state of the auxiliary model will consist solely of the new and past agents (figure 1*f*), assuming that the dynamics of the system are such that all agents have a positive death rate (or more generally, may act as reactants), as is the case with SSLM.

The utility of the auxiliary model is that it allows one to re-construct both the initial (Δt=0) and current (Δt>0) configuration of the primary model. Namely, the initial configuration of the primary model is the union of the original and the past agents ($\gamma_{t}=\gamma_{\Delta t}^{O}\cup \gamma_{\Delta t}^{-}$), whereas the current configuration of the primary model is the union of original and the new agents ($\gamma_{t+\Delta t}=\gamma_{\Delta t}^{O}\cup \gamma_{\Delta t}^{+}$). The spatial correlation function of the auxiliary model thus includes the necessary information for constructing the spatio-temporal correlation function of the primary model. Namely, denoting the spatial correlation function of between agent types A∈{O,+,−} and B∈{O,+,−} in the auxiliary model by$\;k_{\Delta t}^{AB}(x,y)$, we can compute the spatio-temporal correlation function of the primary model as

3.1 $$\begin{array}{ccc} k_{t,\Delta t}(x,y) & = & k_{\Delta t}^{OO}(x,y)+k_{\Delta t}^{O+}(x,y)+k_{\Delta t}^{-O}(x,y)\\ & & \;+k_{\Delta t}^{-+}(x,y),\\ k_{t,\Delta t}(x) & = & k_{\Delta t}^{O}(x) \end{array}$$

If the primary model belongs to the class of RCP models, the auxiliary model will also do so (though see electronic supplementary material for a more technical discussion), and hence it is possible to use earlier established methods to describe the time evolution of its spatial correlation functions (equation (2.4)), and hence by equation (3.1) the spatio-temporal correlations of the primary model.

We next develop the above idea in more detail by defining the auxiliary model corresponding to the SSLM. Let us first consider the process of density-dependent death. If an original agent dies, it becomes a past agent, which will remain in the system indefinitely. If a new agent dies, it will disappear from the system. Thus, the density-independent death induces two reactions for the auxiliary model: change of type from original to past agents and disappearance of new agents, both taking place with rate *m*. Similarly, density-dependent death induces the two reactions, in one of which original agents change their type to become past agents, and in the other one of which new agents disappear. Both of these reactions take place with rate $\sum_{y\in \gamma_{\Delta t}^{O}\cup \gamma_{\Delta t}^{+}}a^{-}(x-y)$, where the sum goes over the union of original and new agents as these are the agents that are actually present in terms of the primary model. Finally, reproduction produces new agents in the auxiliary model at location *y* with the per-unit-area rate $\sum_{x\in \gamma_{\Delta t}^{O}\cup \gamma_{\Delta t}^{+}}a^{+}(x-y)$. Again, the sum goes over the union of original and new agents, as these are the agents that are actually present and thus produce offspring. The full mathematical definition of this auxiliary model is given in the electronic supplementary material.

## 4. Solving spatial and spatio-temporal correlations with a perturbation expansion

While equation (2.4) is exact, it is typically not closed in the sense that the dynamics of the lower order correlation functions depend on higher order correlation functions [5,6], e.g. the dynamics of the first-order correlation function depending on the second-order correlation function in equation (2.5). This means that equation (2.4) cannot be solved analytically or numerically except for some trivial cases, such as the SSLM without density-dependent death. To resolve this issue, [5,6] developed a perturbative approach by which equation (2.4) can be solved approximately, the accuracy of the approximation increasing with increasing length scales of the spatial kernels involved. To do so, all spatial kernels, such as the competition kernel $a^{-}$ and reproduction kernel *a* ^+^ , are scaled as

4.1 $$a_{\varepsilon }(x):=\varepsilon^{d}a(\varepsilon x).\;$$

This scaling preserves the integral of the kernel but makes the spatial interactions increasingly long ranged when ε→0. We denote correlation functions of the scaled process by $k_{\varepsilon ,t}$. The utility of the perturbation expansion is that it results in a mathematically rigorous approximation of correlation functions. Namely, it can be shown [5,6] that for the first-order correlation, i.e. for population density, it holds that

4.2 $$k_{\varepsilon ,t}(x)=q_{t}(\varepsilon x)+\varepsilon^{d}p_{t}(\varepsilon x)+o(\varepsilon^{d}).\;$$

Here $q_{t}(x)$ is the mean-field term, $p_{t}(x)$ is the first-order correction to it, and $o(\varepsilon^{d})$ denotes a term that, when divided by $\varepsilon^{d}$, vanishes when ε→0. For higher orders of the correlation function, the perturbation expansion is most naturally written in terms of the cumulants instead of the correlation functions. The second-order cumulant is defined by u(x,y)=k(x,y)−k(x)k(y), and a general expression for the cumulant of order *n* is given in [5]. It can be shown [5,6] that the second-order cumulant follows the perturbation expansion

4.3 $$u_{\varepsilon ,t}(x,y)=\varepsilon^{d}g_{t}(\varepsilon x,\varepsilon y)+o(\varepsilon^{d}),$$

where $g_{t}(x,y)$ is the leading term of the second-order cumulant.

The key utility of the perturbation expansion is that it produces a closed set of equations that can be solved explicitly. Namely, for the general class of RCP models, the perturbation expansion results in the set of equations [6]:

4.4 $$\frac{\text{d}}{\text{d}t}q_{t}=H_{q}(q_{t}),$$

4.5 $$\frac{\text{d}}{\text{d}t}g_{t}=H_{g}(q_{t},g_{t}),$$

4.6 $$\frac{\text{d}}{\text{d}t}p_{t}=H_{p}(q_{t},g_{t},p_{t}),$$

meaning that one can first solve (analytically or numerically) *q_t_* from equation (4.4), then *g_t_* from equation (4.5), and finally *p_t_* from equation (4.6).

It is worth noting that equations (4.5) and (4.6) are linear in $g_{t}$ and $p_{t}$, respectively, and hence are always solvable analytically, in terms of $q_{t}$. On the other hand, these equations are non-homogeneous, in particular, zero-functions do not solve them. This means that even if the initial distribution of agents is spatially completely random (follows the Poisson distribution), the dynamics will not follow the mean-field model over time but spatial correlation will evolve. Note also that, in the case of agents of different types, all equations (4.4)–(4.6) are vector equations. The reference [6] provides a full mathematical explanation of the equations (4.4)–(4.6), as well as computer code that allows one to automatically generate them for any specific RCP model.

To simplify the notation, we consider in the examples below the case where the initial condition is spatially homogeneous. In this case, the first-order correlation function is independent of spatial location, and we may thus write $q_{t}(x)=q_{t}$ and $p_{t}(x)=p_{t}$. Further, second-order correlation functions and cumulants depend only on the distance between the two points, and thus we may write $g_{t}(x,y)=g_{t}(x-y)$.

While the perturbation expansion for spatial correlations (equations (4.4)–(4.6)) was published earlier [5,6], we now turn to the new result, which is the perturbation expansion for spatio-temporal correlations. To do so, we first extend the definition of the two-point spatial cumulant into the two-point spatio-temporal cumulant as

4.7 $$u_{t,\Delta t}(x,y)=k_{t,\Delta t}(x,y)-k_{t}(x)k_{t+\Delta t}(y).\;$$

In the space-homogeneous case, we can write $u_{t,\Delta t}(x,y)=u_{t,\Delta t}(x-y)$. Applying equation (3.1), we may write the spatio-temporal cumulant of the primary model in terms of spatial cumulants of the auxiliary model,

4.8 $$u_{t,\Delta t}(x)=u_{\Delta t}^{OO}(x)+u_{\Delta t}^{O+}(x)+u_{\Delta t}^{-O}(x)+u_{\Delta t}^{-+}(x).\;$$

Applying next the perturbation expansion, we may write

4.9 $$u_{\varepsilon ,t,\Delta t}(x)=\varepsilon^{d}g_{t,\Delta t}(\varepsilon x)+o(\varepsilon^{d}),$$

where $g_{t,\Delta t}(x)$ is the leading term of the second-order spatio-temporal cumulant. By applying the perturbation expansion for spatial correlation functions to the auxiliary model (for details, see electronic supplementary material), we obtain that, similarly to equation (4.8)

4.10 $$g_{t,\Delta t}(x)=g_{\Delta t}^{OO}(x)+g_{\Delta t}^{O+}(x)+g_{\Delta t}^{-O}(x)+g_{\Delta t}^{-+}(x).$$

Since the leading terms of the second-order spatial cumulants for the auxiliary model can be solved from equation (4.5), one can solve $g_{t,\Delta t}(x)$ by summing these up according to equation (4.10). In the space-homogeneous case, one can, however, solve $g_{t,\Delta t}(x)$ also more directly. Namely, if defining $h_{t,\Delta t}(\xi )={\tilde{g}}_{t,\Delta t}(\xi )+q_{\Delta t}^{O}$, where $\tilde{g}$ denotes the Fourier transform of the function *g*, then $h_{t,\Delta t}$ satisfies a linear equation

4.11 $$\frac{\text{d}}{\text{d}\Delta t}h_{t,\Delta t}=H_{h,\Delta t}(q_{t+\Delta t},h_{t,\Delta t}),$$

where $q_{t+\Delta t}$ is the solution to equation (4.4) at time t+Δt. In contrast with equation (4.5) and (4.6), equation (4.11) is homogeneous, i.e. the zero-function formally solves it, making it efficient for mathematical analyses. We note that at time lag Δt=0 the spatio-temporal cumulant coincides with the spatial cumulant, and hence $g_{t,0}(x)=g_{t}(x)$ and $q_{\Delta t=0}^{O}=q_{t}$, which provide the initial condition for equation (4.10): $h_{t,0}(\xi )={\tilde{g}}_{t}(\xi )+q_{t}$. The equations for $q_{\Delta t}^{O},q_{t+\Delta t}$ and $h_{t,\Delta t}$ form a closed system, and hence it is possible to solve $g_{t,\Delta t}(x)$ at least numerically. In the electronic supplementary material, we derive equation (4.11) for all the processes needed to construct the two example models of this paper. We hypothesize that equation (4.11) holds more generally for any RCP model and expect to prove this in a forthcoming paper.

## 5. Spatio-temporal correlations in the spatial and stochastic logistic model

We next solve the spatio-temporal correlation structure of the SSLM. We first recall from earlier work [5] that in the translationally invariant case, the mean-field equation of the SSLM is given by the deterministic and non-spatial logistic model,

5.1 $$H_{q}(q_{t})=(A^{+}-m)q_{t}-A^{-}q_{t}^{2},$$

where $A^{+}=\int_{R^{d}}a^{+}(x)\text{d}x$ and $A^{-}=\int_{R^{d}}a^{-}(x)\text{d}x$. The equations (4.4)–(4.5) and equation (4.10) allow one to consider how the leading terms of population densities, spatial correlation functions and spatio-temporal correlation functions evolve during transient dynamics (for details, see electronic supplementary material). At the stationary regime, i.e. the limit of t→∞, we recall from [5] that the leading term of the population density converges (assuming a positive initial population density) to

5.2 $$q_{*}=\frac{A^{+}-m}{A^{-}}\;,$$

and that the fixed point to equation (4.5) is given by

5.3 $${\tilde{g}}_{*}(\xi )=q_{*}\frac{{\tilde{a}}^{+}(\xi )-q_{*}{\tilde{a}}^{-}(\xi )}{A^{+}-{\tilde{a}}^{+}(\xi )+q_{*}{\tilde{a}}^{-}(\xi )}.\;$$

As detailed in the electronic supplementary material, for the SSLM the limit of equation (4.10) as t→∞ is described by

5.4 $${\tilde{g}}_{\infty ,\Delta t}(\xi )=(q_{*}+{\tilde{g}}_{*}(\xi ))\exp\{-[A^{+}-{\tilde{a}}^{+}(\xi )+q_{*}{\tilde{a}}^{-}(\xi )]\Delta t\}-q_{*}\exp\{-A^{+}\Delta t\}.$$

**Figure 2.**
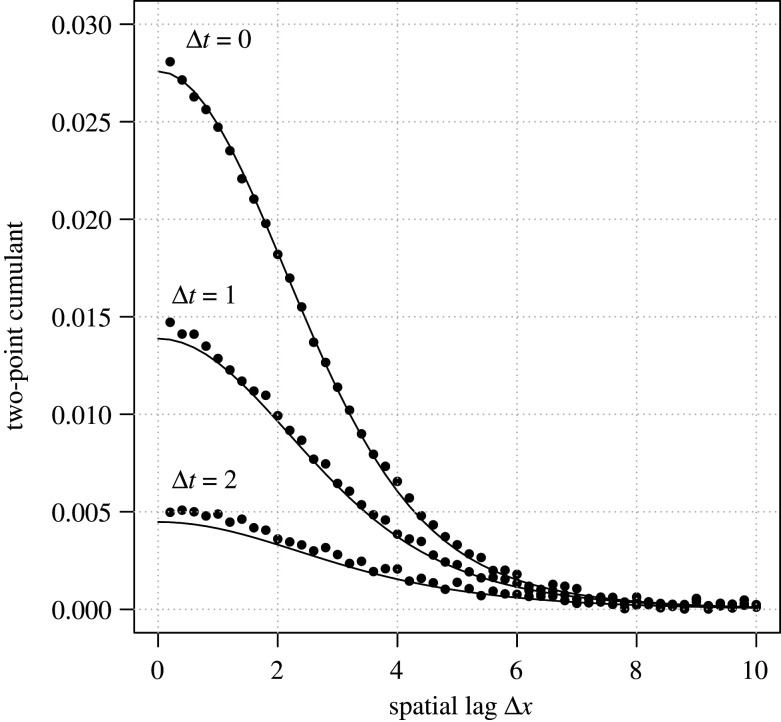
Spatio-temporal correlations in the spatial and stochastic logistic model. Continuous lines are based on analytical calculations and dots are based on simulations. Model parameters were set to $A^{+}=2$, *A*^−^ = 1, and *m* = 1. Both the reproduction kernel *a*^+^ as well as the competition kernel $a^{-}$ were assumed to have a Gaussian shape $\exp(-|x{|}^{2}/2)$ and length scale ε=1/2 . The initial condition was Poisson (complete spatial randomness) with intensity 1. Simulations were conducted with the simulator of [6] in a 800-by-800 unit square with periodic boundary conditions, with the Gaussian kernels truncated with truncation radius 12. Spatio-temporal cumulants were calculated from the time-period between 10 and 30 time units in order to ignore the initial transient. The dots show the averages over three simulation replicates.

Hence, under assumptions on the reproduction and competition kernels described in the electronic supplementary material, in the SSLM the spatio-temporal correlations decay exponentially with increasing time lag Δt, with the rate of decay depending on the frequency parameter ξ in the Fourier space. Moreover, we verify in the electronic supplementary material that with increasing time lag (Δt→∞), the cumulant vanishes also in the real-space $(g_{t,\Delta t}(x)\to 0),$ confirming the intuitively obvious expectation that point configurations become statistically independent of each other if the time between recording them becomes very long. We also note that this property holds in the SSLM not only at the stationary state (t→∞), but more generally also for any fixed *t* (see the electronic supplementary material). To obtain the spatio-temporal correlations in the real-space, the inverse Fourier transform of ${\tilde{g}}_{\Delta t}^{*}$ can be computed numerically. Figure 2 illustrates the match between such a numerically derived solution (the lines) and simulation-based results (the dots) is accurate not only for the two-point spatial cumulant (lines with Δt=0), but also for spatio-temporal cumulants (lines with Δt>0).

## 6. Spatio-temporal correlations in a host–parasite model

To illustrate how the methodology described above applies to the general class of RCP models, we next derive the spatio-temporal correlations for a host–parasite model, called henceforth the HP model. The HP model differs from the SSLM in two qualitative aspects. First, while the SSLM concerns only one type of agents, the HP model involves two types of agents, namely the hosts and the parasites. Second, while the dynamical behaviour of the SSLM is very simple, the HP model shows much richer dynamics, with damping oscillations in the mean-field dynamics and locally synchronized oscillations in the full stochastic and spatial model.

We denote the host and parasite configurations by $\gamma^{H}$ and $\gamma^{P}$, respectively. The model can be defined verbally as follows: hosts produce other hosts with kernel $a^{+}$ and have density-dependent mortality with kernel $a^{-}$. Parasitized hosts turn uninfected hosts into parasitized hosts with kernel *b*, and parasitized hosts have density-independent mortality with rate *m*. The full mathematical description of the model is given in the electronic supplementary material, and its mean-field approximation is given by

6.1 $$\begin{array}{c} \frac{\text{d}}{\text{d}t}q_{t}^{H}=q_{t}^{H}(A^{+}-A^{-}q_{t}^{H}-Bq_{t}^{P})\;\\ \frac{\text{d}}{\text{d}t}q_{t}^{P}=q_{t}^{P}(Bq_{t}^{H}-m)\; \end{array}\}.$$

Here *A*^+^, *A*^−^ and *B* denote the integrals of the kernels *a*^+^, *a*^−^ and *b*, respectively. We note that this HP model can be equally well interpreted as a predator–prey model.

**Figure 3.**
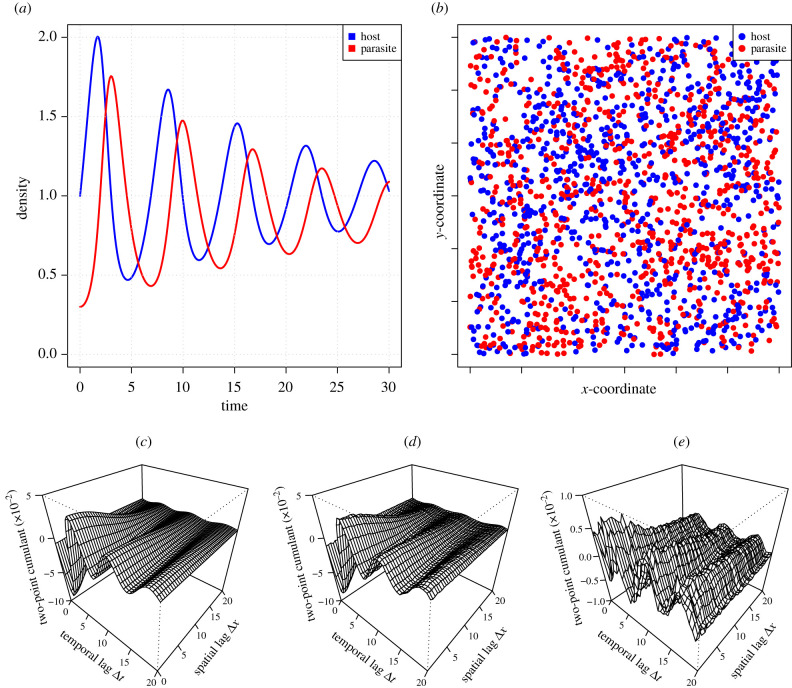
Spatio-temporal dynamics of the host–parasite model. These show mean-field dynamics (*a*), a snapshot of simulations (*b*), and the second-order spatio-temporal cumulant between parasites and hosts as resolved by the mathematical method (*c*), by simulations (*d*) and the difference between these two (*e*). Model parameters were set to $A^{+}=1$, $A^{-}=$ 0.1, *B* = 1 and *m* = 1. All kernels *a*^+^, $a^{-}$ and *b* were assumed to have a Gaussian shape $\exp(-|x{|}^{2}/2)$ and length scale ε=1/2 . The mean-field dynamics (*a*) was solved using deSolve package in R with initial density of hosts set to 1 and that of parasites set to 0.3. Simulations were conducted with the simulator of [6] in a 800-by-800 unit square with periodic boundary conditions, with the Gaussian kernels truncated with truncation radius 6. The snapshot (*b*) is a zoom into a 30-by-30 unit area recorded 70 time units after model initialization. The spatio-temporal cumulant (*d*) was calculated from the time-period between 20 and 70 time units in order to ignore the initial transient using the data from a single simulation. Note the different scale of the *z*-axis in (*e*) as compared to (*c,d*).

Figure 3 illustrates the damping oscillations generated by the parameterization we have chosen for the mean-field model, as well as a spatial patterning of the distribution of hosts and parasites as recorded from the stationary state of the agent-based simulation.

**Figure 4.**
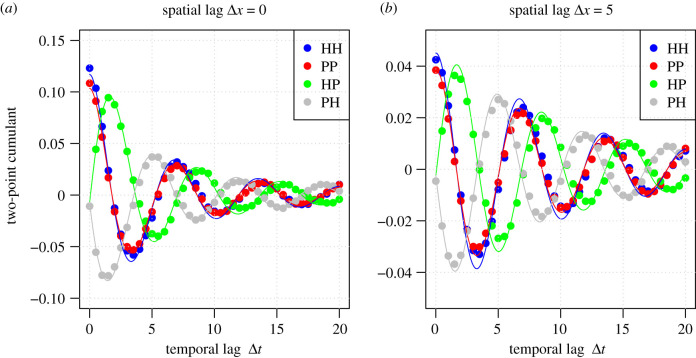
Comparison between mathematical predictions (lines) and simulation-based approximations (dots) of spatial (*a*) and spatio-temporal (*b*) correlations in the HP model. The model parameters, simulation strategy and computation of spatio-temporal cumulants are identical to those in figure 3. The two-point cumulants are shown for host–host (HH), parasite–parasite (PP), host–parasite (HP) and parasite–host (PH).

We computed the leading term of the spatio-temporal cumulant of the HP model by applying the methodology described above, i.e. by first constructing the auxiliary version of the HP model, and then applying the perturbation expansion approach to it (see electronic supplementary material for details). Figure 3 *c*, *d* and *e* show a strikingly good match between the analytically derived (figure 3*c*) and simulation-based (figure 3*d*) spatio-temporal cumulant between parasites (recorded at time *t*) and hosts (recorded at time t+Δt). Note that this two-point cumulant is negative for short time lags, meaning that areas with a high density of parasites will in the near future have a low density of hosts, as expected from the fact that parasites consume hosts. With increasing time lag Δt, the spatio-temporal cumulant shows damping oscillations that alternate between positive and negative values. With increasing spatial lag Δx, the spatio-temporal cumulant expectedly decreases to zero, meaning that the dynamics in locations far away from each other are statistically independent of each other. Figure 4 further shows the two-point cumulant as a function of the time lag Δt for all four pairs between the two types of agents. We observe that all four two-point cumulants oscillate over time, and that the rate of damping in the oscillations is faster for short spatial distances than for large spatial distances. Most importantly, figure 4 illustrates that the mathematical theory (lines) is a very accurate approximation of the simulated behaviour of the agent-based model (dots). Further illustrations, including a movie that visualizes the time evolution of the point configurations, are given in electronic supplementary material.

## 7. Discussion

In this work, we have presented a general methodology for deriving the spatio-temporal correlation structure for the class of RCP models. The main idea behind our method is to define an auxiliary process which, even if being a ‘memoryless' Markov process, keeps track not only on the distribution of the present agents, but also the distribution of the agents at an earlier time. With the help of this property, the spatio-temporal correlations of the original process correspond to the spatial correlations of the auxiliary process, and thus the previously derived toolbox for assessing spatial correlations [5,6] applies also for spatio-temporal correlations. To our knowledge, the method developed here is the first one that can be used to mathematical study the spatio-temporal correlations in spatial Markov processes with local interactions.

In both of the example models we considered, we found the function $H_{g,\Delta t}$ of equation (4.11) to be related in a simple way to the function $H_{g}$ of equation (4.5) (see electronic supplementary material for details). While we are not able to prove that such a simple relationship between $H_{g}$ and $H_{g,\Delta t}$ holds in the general case, we conjecture that this is the case. If the conjecture holds, it would make it even more straightforward to derive spatio-temporal correlations, as one could directly write down the dynamical equation for the spatio-temporal cumulants without the need to first construct the auxiliary model. We thus hope that future work will resolve whether the conjecture holds for all RCP models or some special subset of them.

In both of the example models we considered, we found that the linear equation (4.11) for the auxiliary function $h_{t,\Delta t}={\tilde{g}}_{t,\Delta t}+q_{\Delta t}^{O}$ is homogeneous, making it especially convenient for further analysis. Supported by the fact that this property holds for all of the reactions included in the example models, we conjecture that the same property holds for all RCP models, or at least some large subset of them. We thus hope that future work will find out general form for equation (4.11) in terms of reaction rates of RCP models, as that would enable the straightforward incorporation of spatio-temporal correlation structures into the toolbox of [6].

While the methods presented here apply to the large class of RCP models as such, not all agent-based models can be formulated as RCP models. Namely, many agent-based simulation models that are primarily aimed at simulation tools can be difficult to even define mathematically, and they can combine deterministic and stochastic rules to incorporate e.g. environmental heterogeneity in space and time and processes related to the ageing or learning of the agents. Such models are clearly not possible to implement within the relatively simplistic framework of RCP models, as these models are restricted to continuous space, continuous time, point-like agents and solely stochastic processes. Yet, the fundamental idea shown in figure 1 that forms the basis of this work extends much beyond RCP models.

## Supplementary Material

Mathematical details of the presented method

## Data accessibility

This article has no data.
