# Supplementary material for: A general mathematical method for predicting spatio-temporal correlations emerging from agent-based models
Source: J R Soc Interface. 2020 Oct 28;17(171):20200655. doi: 10.1098/rsif.2020.0655 (PMC7653394; doi:10.1098/rsif.2020.0655)
Supplement: Mathematical details of the presented method [file rsif20200655supp1.pdf]

A general mathematical method for predicting spatiotemporal correlations emerging from agent-based models

by O. Ovaskainen, P. Somervuo, D. Finkelshtein:

Supporting Information

May 9, 2020

Contents

1 Spatial characteristics **2**
- 1.1 Stochastic dynamics 2
- 1.2 Spatial correlation functions and cumulants 3
- 1.3 Beyond mean-field expansion for spatial dynamics 8

2 Spatiotemporal characteristics **11**
- 2.1 Spatiotemporal correlations 11
- 2.2 Dynamics of the auxiliary model 15
- 2.3 Beyond mean-field expansion for spatiotemporal dynamics 16

3 Case study 1: Spatial and stochastic logistic model **21**
- 3.1 Spatial characteristics 21
- 3.2 Spatiotemporal characteristics 24
- 3.3 Derivation of equations 26
- 3.4 Numerics for the stationary regime on plane 29

4 Case study 2: Host-parasite model **31**
- 4.1 Spatial characteristics 31
- 4.2 Spatiotemporal characteristics 35
- 4.3 Derivation of equations 40
- 4.4 Analysis and numerics for the stationary regime 47

A Appendix: Mathematical proofs and discussions **55**

1 Spatial characteristics

1.1 Stochastic dynamics

We consider dynamics of systems consisting of indistinguishable *agents*. Each agent $x^{\mathtt{i}}$ is fully characterized by its position $x \in \mathbb{R}^d$, $d \geq 1$, and its type $\mathtt{i} \in \mathtt{I}_N$, where

$$\mathtt{I}_N = \{s_1, \ldots, s_N\}, \qquad N \geq 1.$$

Here $s_1, \ldots, s_N$ are some fixed labels. We will always assume that there are not two or more agents at the same position.

We will consider discrete systems only, finite or locally finite. The latter means that, if $\gamma_t = \{x^{\mathtt{i}}\}$ is a system of agents at some moment of time $t \geq 0$, then we assume that, in each ball of $\mathbb{R}^d$, there are a finite number of agents from γ_t only. In particular, of course, a finite γ_t is also locally finite. We will call such γ_t a (finite or locally finite) *configuration*.

Each γ_t hence can be expressed as the following *disjoint* union:

$$\gamma_t = \gamma_t^{s_1} \cup \ldots \cup \gamma_t^{s_N} =: (\gamma_t^{s_1}, \ldots, \gamma_t^{s_N}), \tag{1.1}$$

where all agents of $\gamma_t^{\mathtt{i}}$ has the same type $\mathtt{i} \in \mathtt{I}_N$.

Sometimes, we will omit the type of an agent, when it is clear from the context, so that we will write $x \in \gamma_t^{\mathtt{i}}$ rather than $x^{\mathtt{i}} \in \gamma_t^{\mathtt{i}}$. In particular, it will be also in the case where all agents of the system have the same type (i.e. when $N = 1$).

The agents of a configuration are *random*, hence we will speak about random configurations γ_t with respect to (w.r.t. henceforth) a probability distribution. Let Γ denote the space of configurations γ.

The dynamics of configurations in time t is defined through the dynamics of their distributions. Heuristically, the scheme is as follows. We consider a (formal) *Markov generator* on functions $F : \Gamma \to \mathbb{R}$. We will consider the following class of generators, called RCP-generators. Let $\mathtt{R}, \mathtt{C}, \mathtt{P}$ be nonnegative integers, and let

$$r_{\mathtt{R},\mathtt{C},\mathtt{P}}(x_1^{\mathtt{i}_1}, \ldots, x_{\mathtt{R}}^{\mathtt{i}_{\mathtt{R}}}, y_1^{\mathtt{j}_1}, \ldots, y_{\mathtt{C}}^{\mathtt{j}_{\mathtt{C}}}, z_1^{\mathtt{l}_1}, \ldots, z_{\mathtt{P}}^{\mathtt{l}_{\mathtt{P}}}) \geq 0 \tag{1.2}$$

be a function which is symmetric w.r.t. permutations within x-variables, within y-variables, and within z-variables; here $\mathtt{i}_1, \ldots, \mathtt{i}_{\mathtt{R}}, \mathtt{j}_1, \ldots, \mathtt{j}_{\mathtt{C}}, \mathtt{l}_1 \ldots, \mathtt{l}_{\mathtt{P}} \in \mathtt{I}_N$.

We define, for a function F on Γ, the following (formal) operator

$$\begin{aligned}(L_{\mathtt{R},\mathtt{C},\mathtt{P}}F)(\gamma) := &\sum_{\{x_1^{\mathtt{i}_1},\ldots,x_{\mathtt{R}}^{\mathtt{i}_{\mathtt{R}}}\}\subset\gamma} \ \sum_{\{y_1^{\mathtt{j}_1},\ldots,y_{\mathtt{C}}^{\mathtt{j}_{\mathtt{C}}}\}\subset\gamma\setminus\{x_1^{\mathtt{i}_1},\ldots,x_{\mathtt{R}}^{\mathtt{i}_{\mathtt{R}}}\}} \\ &\int_{(\mathbb{R}^d)^{\mathtt{P}}} r_{\mathtt{R},\mathtt{C},\mathtt{P}}(x_1^{\mathtt{i}_1}, \ldots, x_{\mathtt{R}}^{\mathtt{i}_{\mathtt{R}}}, y_1^{\mathtt{j}_1}, \ldots, y_{\mathtt{C}}^{\mathtt{j}_{\mathtt{C}}}, z_1^{\mathtt{l}_1}, \ldots, z_{\mathtt{P}}^{\mathtt{l}_{\mathtt{P}}}) \\ &\times \Bigl(F\bigl(\gamma \setminus \{x_1^{\mathtt{i}_1}, \ldots, x_{\mathtt{R}}^{\mathtt{i}_{\mathtt{R}}}\} \cup \{z_1^{\mathtt{l}_1}, \ldots, z_{\mathtt{P}}^{\mathtt{l}_{\mathtt{P}}}\}\bigr) - F\bigl(\gamma\bigr)\Bigr)\, dz_1 \ldots dz_{\mathtt{P}}.\end{aligned} \tag{1.3}$$

Note that if either of $\mathtt{R}$ and $\mathtt{C}$ is equal to 0, we omit the corresponding sum in (1.3), similarly if $\mathtt{P} = 0$, we omit the integral in (1.3). We assume however that $\mathtt{R} + \mathtt{P} > 0$ otherwise $L_{\mathtt{R},\mathtt{C},\mathtt{P}}F = 0$ for all F.

Finally, we consider a *finite sum* of RCP-operators:

$$L = \sum_{\substack{\mathtt{R},\mathtt{C},\mathtt{P}\geq 0\\ \mathtt{R}+\mathtt{P}>0}} L_{\mathtt{R},\mathtt{C},\mathtt{P}}. \tag{1.4}$$

Operator (1.3), and hence (1.4), has two properties: 1) $L_{\mathtt{R},\mathtt{C},\mathtt{P}}1 = 0$ and 2) if, for a given function F, a configuration γ_* is such that $F(\gamma_*) \geq F(\gamma)$ for all $\gamma \in \Gamma$ (i.e. if γ_* is a global maximum for F), then $(L_{\mathtt{R},\mathtt{C},\mathtt{P}}F)(\gamma_*) \leq 0$. Hence, formally, $L_{\mathtt{R},\mathtt{C},\mathtt{P}}$ and L are Markov generators.

Generator $L_{\mathtt{R},\mathtt{C},\mathtt{P}}$ describes the following *random event*. Let, at a moment of time $t \geq 0$ the system is given by a configuration γ_t. The event is that, within a small time-interval $[t, t+\delta t]$, a group of *reactants* $\{x_1^{\mathtt{i}_1}, \ldots, x_{\mathtt{R}}^{\mathtt{i}_{\mathtt{R}}}\}$ disappears from the configuration and a group of *products* $\{z_1^{\mathtt{l}_1}, \ldots, z_{\mathtt{P}}^{\mathtt{l}_{\mathtt{P}}}\}$ will become a part of the configuration, so that $z_1 \in \Lambda_1$, $\ldots$, $z_{\mathtt{P}} \in \Lambda_{\mathtt{P}}$ for some disjoint bounded subsets $\Lambda_1, \ldots, \Lambda_{\mathtt{P}}$ of $\mathbb{R}^d$. Thus,

$$\gamma_{t+\delta t} = \gamma_t \setminus \{x_1^{\mathtt{i}_1}, \ldots, x_{\mathtt{R}}^{\mathtt{i}_{\mathtt{R}}}\} \cup \{z_1^{\mathtt{l}_1}, \ldots, z_{\mathtt{P}}^{\mathtt{l}_{\mathtt{P}}}\}.$$

The probability of this event is then

$$\delta t \cdot \sum_{\{x_1^{\mathtt{i}_1},\ldots,x_{\mathtt{R}}^{\mathtt{i}_{\mathtt{R}}}\}\subset\gamma} \ \sum_{\{y_1^{\mathtt{j}_1},\ldots,y_{\mathtt{C}}^{\mathtt{j}_{\mathtt{C}}}\}\subset\gamma\setminus\{x_1^{\mathtt{i}_1},\ldots,x_{\mathtt{R}}^{\mathtt{i}_{\mathtt{R}}}\}} \int_{\Lambda_1}\cdots\int_{\Lambda_{\mathtt{P}}} \\ \times r(x_1^{\mathtt{i}_1}, \ldots, x_{\mathtt{R}}^{\mathtt{i}_{\mathtt{R}}}, y_1^{\mathtt{j}_1}, \ldots, y_{\mathtt{C}}^{\mathtt{j}_{\mathtt{C}}}, z_1^{\mathtt{l}_1}, \ldots, z_{\mathtt{P}}^{\mathtt{l}_{\mathtt{P}}})\, dz_1 \ldots dz_{\mathtt{P}} + o(\delta t),$$

where $\lim\limits_{\delta t\to 0} \frac{o(\delta t)}{\delta t} = 0$. The influence on *catalysts* $y_1^{\mathtt{j}_1}, \ldots, y_{\mathtt{C}}^{\mathtt{j}_{\mathtt{C}}}$ reflects the interaction between agents. The catalysts remain unchanged within the event, however, they influence the probability of the event.

We include also the case when, for some m, n, a reactant $x_m^{\mathtt{i}_m}$ and a product $z_n^{\mathtt{l}_n}$ are such that $x_m = z_n$, whereas $\mathtt{i}_m \neq \mathtt{l}_n$, i.e. when an agent keeps its position with changing its type only. In this the corresponding integral w.r.t. dz_n is omitted. We can also formally treat this as like the function r includes the factor $\delta(x_m - z_n)$; henceforth $\delta(x)$ is the Dirac delta-function.

The dynamics of γ_t if defined then through the differential equation:

$$\frac{d}{dt}\mathbb{E}\Big[F(\gamma_t)\Big] = \mathbb{E}\Big[(LF)(\gamma_t)\Big] \tag{1.5}$$

which should be satisfied for a large class of functions F.

1.2 Spatial correlation functions and cumulants

Definition 1.1. For each $\mathtt{i} \in \mathtt{I}_N$, a function $k_t^{\mathtt{i}}(x) \geq 0$ is said to be *the first order spatial correlation function* of type $\mathtt{i}$ (for the distribution of γ_t), if for any function $g_1(x) \geq 0$,

$$\mathbb{E}\Big[\sum_{x\in\gamma_t^{\mathtt{i}}} g_1(x)\Big] = \int_{\mathbb{R}^d} g_1(x) k_t^{\mathtt{i}}(x)\, dx. \tag{1.6}$$

Henceforth, $\mathbb{E}[\cdot]$ denotes the expected value of a random quantity (w.r.t. the distribution of γ_t).

The function $k_t^{\mathtt{i}}(x)$ is also called *the density* of agents of type i, since, taking $g_1(x) = 1\!\!1_\Lambda(x)$ for some bounded subset Λ of $\mathbb{R}^d$, where

$$1\!\!1_\Lambda(x) := \begin{cases} 1, & \text{if } x \in \Lambda, \\ 0, & \text{otherwise,} \end{cases}$$

we get from (1.6) that

$$\mathbb{E}\Big[|\gamma_t^{\mathtt{i}} \cap \Lambda|\Big] = \int_\Lambda k_t^{\mathtt{i}}(x)\,dx.$$

Henceforth, $|\eta|$ denotes number of points in a finite subset η of $\mathbb{R}^d$.

Definition 1.2. For each $\mathtt{i}, \mathtt{j} \in \mathtt{I}_N$, a function $k_t^{\mathtt{i},\mathtt{j}}(x_1, x_2) = k_t^{\mathtt{j},\mathtt{i}}(x_2, x_1) \geq 0$ is said to be *the second-order spatial correlation function* (between agents of the types $\mathtt{i}$ and $\mathtt{j}$), if, for any symmetric function $g_2(x_1, x_2) \geq 0$,

$$\mathbb{E}\Big[\sum_{\substack{x_1 \in \gamma_t^{\mathtt{i}},\ x_2 \in \gamma_t^{\mathtt{j}} \\ x_1 \neq x_2}} g_2(x_1, x_2)\Big] = \int_{\mathbb{R}^d}\int_{\mathbb{R}^d} g_2(x_1, x_2) k_t^{\mathtt{i},\mathtt{j}}(x_1, x_2)\,dx_1 dx_2. \tag{1.7}$$

Remark 1.3. Recall, we assume that agents cannot occupy the same position, hence, for $\mathtt{i} \neq \mathtt{j}$, $\gamma_t^{\mathtt{i}}$ and $\gamma_t^{\mathtt{j}}$ are disjoint, and thus the restriction $x_1 \neq x_2$ in (1.7) is redundant then.

Combining (1.7) with (1.6), we can also write, for all $\mathtt{i}, \mathtt{j} \in \mathtt{I}_N$,

$$\begin{aligned}\mathbb{E}\Big[\sum_{x_1 \in \gamma^{\mathtt{i}},\ x_2 \in \gamma^{\mathtt{j}}} g_2(x_1, x_2)\Big] \\ = \int_{\mathbb{R}^d}\int_{\mathbb{R}^d} g_2(x_1, x_2) k_t^{\mathtt{i},\mathtt{j}}(x_1, x_2)\,dx_1 dx_2 + 1\!\!1_{\mathtt{i}=\mathtt{j}} \int_{\mathbb{R}^d} g_2(x, x) k_t^{\mathtt{i}}(x)\,dx. \end{aligned}\tag{1.8}$$

Henceforth, $1\!\!1_{\mathtt{i}=\mathtt{j}}$ denotes the Kronecker delta (that is 1 if $\mathtt{i} = \mathtt{j}$ and 0 otherwise).

Substituting to (1.8) the symmetric function

$$g_2(x_1, x_2) = \frac{1}{2}\Big(1\!\!1_{\Lambda_1}(x_1)1\!\!1_{\Lambda_2}(x_2) + 1\!\!1_{\Lambda_1}(x_2)1\!\!1_{\Lambda_2}(x_1)\Big), \tag{1.9}$$

where Λ_1, Λ_2 are bounded subsets of $\mathbb{R}^d$, we get

$$\begin{aligned}\mathbb{E}\Big[|\gamma_t^{\mathtt{i}} \cap \Lambda_1|\,|\gamma_t^{\mathtt{j}} \cap \Lambda_2|\Big] &= \int_{\Lambda_1}\int_{\Lambda_2} k_t^{\mathtt{i},\mathtt{j}}(x_1, x_2)\,dx_1 dx_2 \\ &\quad + 1\!\!1_{\mathtt{i}=\mathtt{j}} \int_{\Lambda_1 \cap \Lambda_2} k_t^{\mathtt{i}}(x)\,dx. \end{aligned}\tag{1.10}$$

One can also consider *the centralized spatial moment* that is the expectation of the product of centralized random quantities $|\gamma_t^{\mathtt{i}} \cap \Lambda_1| - \mathbb{E}[|\gamma_t^{\mathtt{i}} \cap \Lambda_1|]$, $\mathtt{i} \in \mathtt{I}_N$ (called so because the expectation of each such quantity is 0):

$$\begin{aligned}
&\mathbb{E}\Big[\Big(|\gamma_t^{\mathtt{i}} \cap \Lambda_1| - \mathbb{E}\big[|\gamma_t^{\mathtt{i}} \cap \Lambda_1|\big]\Big)\Big(|\gamma_t^{\mathtt{j}} \cap \Lambda_2| - \mathbb{E}\big[|\gamma_t^{\mathtt{j}} \cap \Lambda_2|\big]\Big)\Big] \\
&= \mathbb{E}\Big[|\gamma_t^{\mathtt{i}} \cap \Lambda_1|\, |\gamma_t^{\mathtt{j}} \cap \Lambda_2|\Big] - \mathbb{E}\Big[|\gamma_t^{\mathtt{i}} \cap \Lambda_1|\Big]\, \mathbb{E}\Big[|\gamma_t^{\mathtt{j}} \cap \Lambda_2|\Big] \\
&= \int_{\Lambda_1}\int_{\Lambda_2} \Big(k_t^{\mathtt{i},\mathtt{j}}(x_1,x_2) - k_t^{\mathtt{i}}(x_1)\, k_t^{\mathtt{j}}(x_2)\Big)\, dx_1 dx_2 + 1\!\!1_{\mathtt{i}=\mathtt{j}} \int_{\Lambda_1\cap\Lambda_2} k_t^{\mathtt{i}}(x)\, dx.
\end{aligned}$$

Definition 1.4. The function

$$u_t^{\mathtt{i},\mathtt{j}}(x_1,x_2) := k_t^{\mathtt{i},\mathtt{j}}(x_1,x_2) - k_t^{\mathtt{i}}(x_1)\, k_t^{\mathtt{j}}(x_2), \tag{1.11}$$

is called *the second order spatial cumulant* between types $\mathtt{i}$ and $\mathtt{j}$.

We have hence

$$\begin{aligned}
&\mathbb{E}\Big[\Big(|\gamma_t^{\mathtt{i}} \cap \Lambda_1| - \mathbb{E}\big[|\gamma_t^{\mathtt{i}} \cap \Lambda_1|\big]\Big)\Big(|\gamma_t^{\mathtt{j}} \cap \Lambda_2| - \mathbb{E}\big[|\gamma_t^{\mathtt{j}} \cap \Lambda_2|\big]\Big)\Big] \\
&= \int_{\Lambda_1}\int_{\Lambda_2} u_t^{\mathtt{i},\mathtt{j}}(x_1,x_2)\, dx_1 dx_2 + 1\!\!1_{\mathtt{i}=\mathtt{j}} \int_{\Lambda_1\cap\Lambda_2} k_t^{\mathtt{i}}(x)\, dx.
\end{aligned} \tag{1.12}$$

We going to define now a general spatial correlation function.

Definition 1.5. Consider $n \in \mathbb{N}$ types $\mathtt{i}_1, \ldots, \mathtt{i}_n \in \mathtt{I}_N$ (some types may coincide). We define *n-th order spatial correlation function* $k_t^{\mathtt{i}_1,\ldots,\mathtt{i}_n}(x_1,\ldots,x_n) \geq 0$ as such that, for any symmetric function $g_n(x_1,\ldots,x_n) \geq 0$,

$$\mathbb{E}\Bigg[\sum_{\substack{x_1\in\gamma_t^{\mathtt{i}_1},\ \ldots,\ x_n\in\gamma_t^{\mathtt{i}_n} \\ x_j\neq x_l \text{ for } j\neq l}} g_n(x_1,\ldots,x_n)\Bigg] = \int_{\mathbb{R}^d}\cdots\int_{\mathbb{R}^d} g_n(x_1,\ldots,x_n) k_t^{\mathtt{i}_1,\ldots,\mathtt{i}_n}(x_1,\ldots,x_n)\, dx_1\ldots dx_n. \tag{1.13}$$

Remark 1.6. If $\sigma \in S_n$ is a permutation of $(1,\ldots,n)$ then

$$k_t^{\mathtt{i}_1,\ldots,\mathtt{i}_n}(x_1,\ldots,x_n) = k_t^{\mathtt{i}_{\sigma(1)},\ldots,\mathtt{i}_{\sigma(n)}}(x_{\sigma(1)},\ldots,x_{\sigma(n)}).$$

Remark 1.7. If $N = 1$, so that $\mathtt{i}_1 = \ldots = \mathtt{i}_n = s_1$, then we will normally use the notation:

$$k_t^{(n)}(x_1,\ldots,x_n) := k_t^{s_1,\ldots,s_1}(x_1,\ldots,x_n).$$

In particular, $k_t^{(1)}(x) := k_t^{s_1}(x)$. We can also rewrite then (1.13) as follows,

$$\mathbb{E}\Big[\sum_{\{x_1,\ldots,x_n\}\subset\gamma_t} g_n(x_1,\ldots,x_n)\Big] = \frac{1}{n!}\int_{\mathbb{R}^d}\cdots\int_{\mathbb{R}^d} g_n(x_1,\ldots,x_n) k_t^{(n)}(x_1,\ldots,x_n);\, dx_1\ldots dx_n.$$

and the spatial correlation function $k_t^{(n)}$ is also called *the n-th order spatial factorial moment*. Note that $k_t^{(n)}$ is a symmetric function.

To define *n-th order spatial cumulants*, we note that $[\mathtt{i}_1, \ldots, \mathtt{i}_n]$ is called a *multiset*, i.e. a collection of n (possibly repeating) elements from $\mathtt{I}_N$.

Definition 1.8. We set $u_t^{\mathtt{i}}(x) := k_t^{\mathtt{i}}(x)$, $\mathtt{i} \in \mathtt{I}_N$, and define spatial cumulants through the equality

$$k_t^{\mathtt{i}_1,\ldots,\mathtt{i}_n}(x_1, \ldots, x_n) = \sum u_t^{\mathtt{j}_1^{(1)},\ldots,\mathtt{j}_{n_1}^{(1)}}(x_1^{(1)}, \ldots, x_{n_1}^{(1)}) \ldots u_t^{\mathtt{j}_1^{(m)},\ldots,\mathtt{j}_{n_m}^{(m)}}(x_1^{(m)}, \ldots, x_{n_m}^{(m)}), \quad (1.14)$$

where sum is taken over all multiset partitions

$$[\mathtt{i}_1, \ldots, \mathtt{i}_n] = [\mathtt{j}_1^{(1)}, \ldots, \mathtt{j}_{n_1}^{(1)}] \cup \ldots \cup [\mathtt{j}_1^{(m)}, \ldots, \mathtt{j}_{n_m}^{(m)}]$$

so that $1 \le m \le n$, $n_1 + \ldots + n_m = n$ and

$$\{x_1, \ldots, x_n\} = \{x_1^{(1)}, \ldots, x_{n_1}^{(1)}\} \cup \ldots \cup \{x_1^{(m)}, \ldots, x_{n_m}^{(m)}\}.$$

Remark 1.9. To see that (1.14) indeed defines spatial cumulant u_t for given spatial correlation functions k_t, note that the right hand side (r.h.s. henceforth) of (1.14) contains the term with $m = 1$ which is just $u_t^{\mathtt{i}_1,\ldots,\mathtt{i}_n}(x_1, \ldots, x_n)$ (since then $n_1 = n$), i.e. the spatial cumulant of the same order as the spatial correlation function $k_t^{\mathtt{i}_1,\ldots,\mathtt{i}_n}(x_1, \ldots, x_n)$. For $2 \le m \le n$, we have $n_1 < n$, …, $n_m < n$, hence all other terms correspond to products of spatial cumulants of smaller orders. Hence, one can get $u_t^{\mathtt{i}_1,\ldots,\mathtt{i}_n}(x_1, \ldots, x_n)$ inductively, e.g., cf. (1.11), for any $\mathtt{i}, \mathtt{j}, \mathtt{l} \in \mathtt{I}_N$,

$$\begin{aligned} u_t^{\mathtt{i},\mathtt{j}}(x_1, x_2) &= k_t^{\mathtt{i},\mathtt{j}}(x_1, x_2) - u_t^{\mathtt{i}}(x_1)\, u_t^{\mathtt{j}}(x_2), \\ u_t^{\mathtt{i},\mathtt{j},\mathtt{l}}(x_1, x_2, x_3) &= k_t^{\mathtt{i},\mathtt{j},\mathtt{l}}(x_1, x_2) - u_t^{\mathtt{i},\mathtt{j}}(x_1, x_2)\, u_t^{\mathtt{l}}(x_3) \\ &\qquad - u_t^{\mathtt{i},\mathtt{l}}(x_1, x_3)\, u_t^{\mathtt{j}}(x_2) - u_t^{\mathtt{j},\mathtt{l}}(x_2, x_3)\, u_t^{\mathtt{i}}(x_1). \end{aligned}$$

Differential equations for spatial correlation functions can be obtained from (1.5) by using the definition (1.13). Namely, we take as F in (1.5) the integrand in the left hand side (l.h.s. henceforth) of (1.13), i.e. $F = F_n$, where

$$F(\gamma_t) = F_n(\gamma_t^{\mathtt{i}_1}, \ldots, \gamma_t^{\mathtt{i}_n}) = \sum_{\substack{x_1 \in \gamma_t^{\mathtt{i}_1} \ldots x_n \in \gamma_t^{\mathtt{i}_n} \\ x_j \neq x_l \text{ for } j \neq l}} g_n(x_1, \ldots, x_n),$$

where g_n is a symmetric function such that, for some bounded subset Λ of $\mathbb{R}^d$, $g_n(x_1, \ldots, x_n) = 0$ if only $x_m \notin \Lambda$ for some $1 \le m \le n$.

Differentiating both part of (1.13), we will get then from (1.5) that

$$\int_{(\mathbb{R}^d)^n} g_n(x_1, \ldots, x_n)\, \frac{d}{dt} k_t^{\mathtt{i}_1,\ldots,\mathtt{i}_n}(x_1, \ldots, x_n)\, dx_1 \ldots dx_n = \mathbb{E}\Big[(LF_n)(\gamma_t)\Big],$$

where, recall, $F = F(g_n)$.

The next step is to represent

$$(LF)(\gamma_t) = \sum_m \sum_{\substack{x_1\in\gamma_t^{\mathtt{j}_1},\ \ldots,\ x_m\in\gamma_t^{\mathtt{j}_m}\\ x_i\neq x_l \text{ for } i\neq l}} \widehat{g}_m(x_1,\ldots,x_m),$$

where $\widehat{g}_m$ are also symmetric functions depending on g_n, and types $\mathtt{j}_1,\ldots,\mathtt{j}_m \in \mathtt{I}_N$; they all depend on the particular form of the operator L; in the case of an RCP-generator, $\widehat{g}_m$ and types depend on the rate (1.2). Note that then the summation in m is finite.

We will get then, by (1.13),

$$\begin{aligned}\int_{(\mathbb{R}^d)^n} & g_n(x_1,\ldots,x_n)\,\frac{d}{dt}k_t^{\mathtt{i}_1,\ldots,\mathtt{i}_n}(x_1,\ldots,x_n)\,dx_1\ldots dx_n\\ &= \sum_m \int_{(\mathbb{R}^d)^m} \widehat{g}_m(x_1,\ldots,x_m)\,k_t^{\mathtt{j}_1,\ldots,\mathtt{j}_m}(x_1,\ldots,x_m)\,dx_1\ldots dx_m.\end{aligned}$$

Since F_n depends on g_n linearly and LF depends on F linearly, we have that $\widehat{L}_{m,n} g_n := \widehat{g}_m$ depend on g_n linearly as well. By considering a dual operator $L^{\triangle}_{n,m} := (\widehat{L}_{m,n})^*$, we will get that

$$\begin{aligned}\int_{(\mathbb{R}^d)^n} & g_n(x_1,\ldots,x_n)\,\frac{d}{dt}k_t^{\mathtt{i}_1,\ldots,\mathtt{i}_n}(x_1,\ldots,x_n)\,dx_1\ldots dx_n\\ &= \int_{(\mathbb{R}^d)^n} g_n(x_1,\ldots,x_n)\,\sum_m \bigl(L^{\triangle}_{n,m} k_t^{\mathtt{j}_1,\ldots,\mathtt{j}_m}\bigr)(x_1,\ldots,x_n)\,dx_1\ldots dx_n.\end{aligned}$$

Since g_n was arbitrary, we get then

$$\frac{d}{dt}k_t^{\mathtt{i}_1,\ldots,\mathtt{i}_n}(x_1,\ldots,x_n) = \sum_m \bigl(L^{\triangle}_{n,m} k_t^{\mathtt{j}_1,\ldots,\mathtt{j}_m}\bigr)(x_1,\ldots,x_n). \tag{1.15}$$

Considering an infinite vector k_t of all functions $k_t^{\mathtt{i}_1,\ldots,\mathtt{i}_n}$ indexed by $n \geq 1$ and by different multisets $[\mathtt{i}_1,\ldots,\mathtt{i}_n]$ of types, we can treat the r.h.s. of (1.15) as the action of an infinite matrix $L^{\triangle}$, whose entries are operators $L^{\triangle}_{n,m}$.

Stress that, typically, m can take values larger than n, so that the system of *linear* differential equations (1.15) is not closed and cannot be solved analytically nor numerically.

The explicit form for the action of $L^{\triangle}$ in case of the RCP-generator L, given by (1.3), can be found in [1, Supplementary Note 1].

The differential equations for spatial cumulants can be obtained by substituting (1.14) into (1.15). The equations will have a similar form

$$\frac{d}{dt}u_t^{\mathtt{i}_1,\ldots,\mathtt{i}_n}(x_1,\ldots,x_n) = \sum_m \bigl(Q^{\triangle}_{n,m} u_t^{\mathtt{j}_1,\ldots,\mathtt{j}_m}\bigr)(x_1,\ldots,x_n),$$

with, however, *nonlinear* operators $Q^{\triangle}_{n,m}$. For their explicit form, in the case of L given by (1.4), we also refer to [1, Supplementary Note 1].

1.3 Beyond mean-field expansion for spatial dynamics

Equation (1.15) has initial conditions at, say, time $t = 0$: $k_0^{\mathtt{i}_1,\dots,\mathtt{i}_n}(x_1,\dots,x_n)$. The important class of such initial conditions are product functions

$$k_0^{\mathtt{i}_1,\dots,\mathtt{i}_n}(x_1,\dots,x_n) = q_0^{\mathtt{i}_1}(x_1)\dots q_0^{\mathtt{i}_n}(x_n), \tag{1.16}$$

where $q_0^{\mathtt{i}_1}$, ..., $q_0^{\mathtt{i}_n}$ are nonnegative functions on $\mathbb{R}^d$. Spatial correlation function (1.16) corresponds to the Poisson distribution of configurations. The characteristic feature of the Poisson distribution is that random numbers

$$|\gamma_0^{\mathtt{i}_1} \cap \Lambda_1|, \dots, |\gamma_0^{\mathtt{i}_n} \cap \Lambda_n|$$

are independent for all *disjoint* bounded subsets $\Lambda_1,\dots,\Lambda_n$ of $\mathbb{R}^d$; in particular, all corresponding spatial cumulants of an order more than 1 are equal to 0, cf. (1.12). The Poisson distribution is also called *chaotic* because of the mentioned independence.

In most cases, however, the solution to (1.15) with the initial condition (1.16) does not have a product structure. The idea of the mean-field approximation (with a *small* parameter $\varepsilon > 0$) is to find a modification L_ε of the Markov operator L in (1.5), such that the solution $k_{\varepsilon,t}^{\mathtt{i}_1,\dots,\mathtt{i}_n}(x_1,\dots,x_n)$ to the corresponding equation, cf. (1.15),

$$\frac{d}{dt}k_{\varepsilon,t}^{\mathtt{i}_1,\dots,\mathtt{i}_n}(x_1,\dots,x_n) = \sum_m \big(L^{\triangle}_{\varepsilon,n,m} k_{\varepsilon,t}^{\mathtt{j}_1,\dots,\mathtt{j}_m}\big)(x_1,\dots,x_n) \tag{1.17}$$

would be *approximately* (up to certain order of ε) equal to a product function. Hence the distribution of $\gamma_{\varepsilon,t}$ would be approximately chaotic, in a certain sense. This is called *the propagation of chaos* in statistical physics.

The realization of the scaling procedure for the RCP-generator is as follows. We assume that r in (1.2) is given through combinations of various kernels of the form

$$a(v^{\mathtt{k}}, w^{\mathtt{m}}) = a_{\mathtt{k},\mathtt{m}}(v - w), \qquad \text{with } a_{\mathtt{k},\mathtt{m}}(-v) = a_{\mathtt{k},\mathtt{m}}(v), \tag{1.18}$$

where $v, w \in \mathbb{R}^d$, $\mathtt{k},\mathtt{m} \in \mathtt{I}_N$, $a_{\mathtt{k},\mathtt{m}} \ge 0$ is a function on $\mathbb{R}^d$. Here $v^{\mathtt{k}}, w^{\mathtt{m}}$ are some agents among reactants $x_1^{\mathtt{i}_1},\dots,x_{\mathtt{R}}^{\mathtt{i}_\mathtt{R}}$, catalysts $y_1^{\mathtt{j}_1},\dots,y_{\mathtt{C}}^{\mathtt{j}_\mathtt{C}}$ or products $z_1^{\mathtt{l}_1},\dots,z_{\mathtt{P}}^{\mathtt{l}_\mathtt{P}}$.

We consider L_ε given by (1.4) with $r_{\mathtt{R},\mathtt{C},\mathtt{P}}$ in (1.3) replaced by

$$(\varepsilon^d)^{\mathtt{R}+\mathtt{C}+\mathtt{P}-1} r_{\varepsilon,\mathtt{R},\mathtt{C},\mathtt{P}}(x_1^{\mathtt{i}_1},\dots,x_{\mathtt{R}}^{\mathtt{i}_\mathtt{R}}, y_1^{\mathtt{j}_1},\dots,y_{\mathtt{C}}^{\mathtt{j}_\mathtt{C}}, z_1^{\mathtt{l}_1},\dots,z_{\mathtt{P}}^{\mathtt{l}_\mathtt{P}}), \tag{1.19}$$

where $r_{\varepsilon,\mathtt{R},\mathtt{C},\mathtt{P}}$ has the same structure as $r_{\mathtt{R},\mathtt{C},\mathtt{P}}$, however, the kernels $a(v^{\mathtt{k}}, w^{\mathtt{m}})$, given previously by (1.18), are replaced now by

$$a_\varepsilon(v^{\mathtt{k}}, w^{\mathtt{m}}) = \varepsilon^d a_{\mathtt{k},\mathtt{m}}(\varepsilon v - \varepsilon w).$$

Note that

$$\int_{\mathbb{R}^d} \varepsilon^d a_{\mathtt{k},\mathtt{m}}(\varepsilon x)\,dx = \int_{\mathbb{R}^d} a_{\mathtt{k},\mathtt{m}}(x)\,dx,$$

i.e. the scaled kernels have the same full integral but a scaled (expanded) shape.

Next, we consider the initial condition to the corresponding equation (1.17), as follows

$$k_{\varepsilon,0}^{\mathtt{i}_1,\ldots,\mathtt{i}_n}(x_1,\ldots,x_n) = q_0^{\mathtt{i}_1}(\varepsilon x_1)\ldots q_0^{\mathtt{i}_n}(\varepsilon x_n) + o(1), \tag{1.20}$$

where $\lim\limits_{\varepsilon\to 0} o(1) = 0$ (in particular, one can consider the initial condition without that $o(1)$ at all). The statement is that then the solution to (1.17) has the property

$$k_{\varepsilon,t}^{\mathtt{i}_1,\ldots,\mathtt{i}_n}(x_1,\ldots,x_n) = q_t^{\mathtt{i}_1}(\varepsilon x_1)\ldots q_t^{\mathtt{i}_n}(\varepsilon x_n) + o(1), \tag{1.21}$$

where $q_t^{\mathtt{i}}$, $\mathtt{i}\in\mathtt{I}_N$, solve a system of (nonlinear) differential equations

$$\frac{d}{dt}q_t^{\mathtt{i}}(x) = H_q^{\mathtt{i}}\big(\overline{q}_t\big)(x), \qquad \mathtt{i}\in\mathtt{I}_N, \tag{1.22}$$

where $\overline{q}_t$ is the vector of all $q_t^{s_1},\ldots,q_t^{s_N}$; with certain (nonlinear) mappings $H_q^{s_1}$, $\ldots$, $H_q^{s_N}$. For the exact form of $H_q^{\mathtt{i}}$, $\mathtt{i}\in\mathtt{I}_N$, we refer to [1, Supplementary Note 1].

By (1.21), the cumulants of all orders bigger than 1 corresponding to the function $k_{\varepsilon,t}^{\mathtt{i}_1,\ldots,\mathtt{i}_n}(x_1,\ldots,x_n)$, $n\geq 2$, through expansion (1.14) are equal to $o(1)$. In particular, cf. (1.11),

$$u_{\varepsilon,t}^{\mathtt{i},\mathtt{j}}(x_1,x_2) = k_{\varepsilon,t}^{\mathtt{i},\mathtt{j}}(x_1,x_2) - k_{\varepsilon,t}^{\mathtt{i}}(x_1)\,k_{\varepsilon,t}^{\mathtt{j}}(x_2) = o(1). \tag{1.23}$$

The term $o(1)$ in (1.21) depends in a non-trivial way on both time t, variables $x_1,\ldots,x_n$ and types $\mathtt{i}_1,\ldots,\mathtt{i}_n$. To partially reveal this dependence, one needs the next term of the expansion.

It was shown in [1, Supplementary Note 1], that

$$\begin{aligned} k_{\varepsilon,t}^{\mathtt{i}}(x) = u_{\varepsilon,t}^{\mathtt{i}}(x) &= q_t^{\mathtt{i}}(\varepsilon x) + \varepsilon^d p_t^{\mathtt{i}}(\varepsilon x) + o(\varepsilon^d),\\ u_{\varepsilon,t}^{\mathtt{i},\mathtt{j}}(x_1,x_2) &= \varepsilon^d g_t^{\mathtt{i},\mathtt{j}}(\varepsilon x_1,\varepsilon x_2) + o(\varepsilon^d), \end{aligned} \tag{1.24}$$

where $\lim\limits_{\varepsilon\to 0}\frac{o(\varepsilon^d)}{\varepsilon^d} = 0$. Here $q_t^{\mathtt{i}}$ satisfies (1.22) and $p_t^{\mathtt{i}}, g_t^{\mathtt{i},\mathtt{j}}$ satisfy certain *linear* differential equations

$$\frac{d}{dt}g_t^{\mathtt{i},\mathtt{j}}(x_1,x_2) = H_g^{\mathtt{i},\mathtt{j}}[\overline{q}_t]\big(\overline{g}_t\big)(x_1,x_2), \tag{1.25}$$

$$\frac{d}{dt}p_t^{\mathtt{i}}(x) = H_p^{\mathtt{i}}[\overline{q}_t]\big(\overline{g}_t,\overline{p}_t\big)(x), \tag{1.26}$$

where

- $\overline{q}_t$ is a vector of all $q_t^{\mathtt{i}}$, $\mathtt{i}\in\mathtt{I}_N$, that solve (1.22);
- $\overline{p}_t$ is a vector of all $p_t^{\mathtt{i}}$, $\mathtt{i}\in\mathtt{I}_N$; and $\overline{g}_t$ is the vector of all $g_t^{\mathtt{i},\mathtt{j}}$, $\mathtt{i},\mathtt{j}\in\mathtt{I}_N$;
- $H_g^{\mathtt{i},\mathtt{j}}[\overline{q}_t](\cdot)$ and $H_p^{\mathtt{i}}[\overline{q}_t](\cdot)$ are multilinear mapping, i.e. both $H_g^{\mathtt{i},\mathtt{j}}[\overline{q}_t]\big(\overline{g}_t\big)$ and $H_p^{\mathtt{i}}[\overline{q}_t](\overline{g}_t,\overline{p}_t)$ depend linearly on *each* $g_t^{\mathtt{i},\mathtt{j}}$, $p_t^{\mathtt{l}}$, $\mathtt{i},\mathtt{j},\mathtt{l}\in\mathtt{I}_N$;
- mappings $H_p^{\mathtt{i}}[\overline{q}_t]$ and $H_g^{\mathtt{i},\mathtt{j}}[\overline{q}_t]$ depends on $q_t^{\mathtt{i}}$ in a nonlinear (in general) way.

One can also get then from (1.23) the following enhancement of (1.21) for $n = 2$:

$$k^{\mathtt{i},\mathtt{j}}_{\varepsilon,t}(x_1,x_2) = q^{\mathtt{i}}_t(\varepsilon x_1)q^{\mathtt{j}}_t(\varepsilon x_2) + \varepsilon^d g^{\mathtt{i},\mathtt{j}}_t(\varepsilon x_1,\varepsilon x_2) + \varepsilon^d\Big(q^{\mathtt{i}}_t(\varepsilon x_1)\,p^{\mathtt{j}}_t(\varepsilon x_2) + p^{\mathtt{i}}_t(\varepsilon x_1)\,q^{\mathtt{j}}_t(\varepsilon x_2)\Big) + o(\varepsilon^d). \tag{1.27}$$

Space-homogeneous case Consider the special case, where, initially, the density does not depend on space and the pair-correlation is translation invariant, namely:

$$\begin{aligned} k^{\mathtt{i}}_{\varepsilon,0}(x) &= k^{\mathtt{i}}_{\varepsilon,0} = q^{\mathtt{i}}_0 + \varepsilon^d p^{\mathtt{i}}_0 + o(\varepsilon^d),\\ k^{\mathtt{i},\mathtt{j}}_{\varepsilon,0}(x_1,x_2) &= k^{\mathtt{i},\mathtt{j}}_{\varepsilon,0}(x_1-x_2) = q^{\mathtt{i}}_0 q^{\mathtt{j}}_0 + \varepsilon^d g^{\mathtt{i},\mathtt{j}}_0(x_1-x_2) + o(\varepsilon^d). \end{aligned} \tag{1.28}$$

Then if the operator L_ε has the form (1.4) with $r_{\mathtt{R},\mathtt{C},\mathtt{P}}$ in (1.3) replaced by $r_{\varepsilon,\mathtt{R},\mathtt{C},\mathtt{P}}$ which is a combination of pair-interaction kernels as above, then, for all $t \geq 0$, $k^{\mathtt{i}}_{\varepsilon,t}(x)$ does not depend on x and $k^{\mathtt{i},\mathtt{j}}_{\varepsilon,t}(x_1,x_2)$ depends on $x_1 - x_2$:

$$\begin{aligned} k^{\mathtt{i}}_{\varepsilon,t}(x) &= k^{\mathtt{i}}_{\varepsilon,t} = q^{\mathtt{i}}_t + \varepsilon^d p^{\mathtt{i}}_t + o(\varepsilon^d),\\ k^{\mathtt{i},\mathtt{j}}_{\varepsilon,t}(x_1,x_2) &= k^{\mathtt{i},\mathtt{j}}_{\varepsilon,t}(x_1-x_2) = q^{\mathtt{i}}_t q^{\mathtt{j}}_t + \varepsilon^d g^{\mathtt{i},\mathtt{j}}_t(x_1-x_2) + o(\varepsilon^d), \end{aligned}$$

where $q^{\mathtt{i}}_t$, $\mathtt{i} \in \mathtt{I}_N$, satisfy the system (1.22) of ordinary differential equation, and (see [1, Supplementary Note 1, formula (241)]) the equation (1.25) can be rewritten in terms of the Fourier transform of functions $g^{\mathtt{i},\mathtt{j}}_t(x)$, defined by

$$\widetilde{g}^{\mathtt{i},\mathtt{j}}_t(\xi) := \int_{\mathbb{R}^d} g^{\mathtt{i},\mathtt{j}}_t(x) e^{-2\,i\,\pi\,x\cdot\xi}\,dx, \qquad \xi \in \mathbb{R}^d, \tag{1.29}$$

where $x \cdot \xi$ denotes the standard dot-product in $\mathbb{R}^d$ and $i^2 = -1$.

Namely, $\widetilde{g}^{\mathtt{i},\mathtt{j}}_t(\xi)$ satisfies the following differential equation, for each $\xi \in \mathbb{R}^d$,

$$\frac{d}{dt}\widetilde{g}^{\mathtt{i},\mathtt{j}}_t(\xi) = \mathscr{C}^{\mathtt{i},\mathtt{j}}[\overline{q}_t]\,\overline{\widetilde{g}}_t(\xi) + \mathscr{D}^{\mathtt{i},\mathtt{j}}[\overline{q}_t](\xi). \tag{1.30}$$

Here, similarly to above,

- $\overline{q}_t$ is the vector of all $q^{\mathtt{i}}_t$, $\mathtt{i} \in \mathtt{I}_N$, , which solve (1.22)
- $\overline{\widetilde{g}}_t$ is the vector of all $\widetilde{g}^{\mathtt{i},\mathtt{j}}_t(\xi)$, $\mathtt{i},\mathtt{j} \in \mathtt{I}_N$;
- $\mathscr{C}^{\mathtt{i},\mathtt{j}}[\overline{q}_t](\cdot)$ is a multilinear mapping, so that $\mathscr{C}^{\mathtt{i},\mathtt{j}}[\overline{q}_t](\overline{\widetilde{g}}_t)$ depends linearly on each $\widetilde{g}^{\mathtt{i},\mathtt{j}}_t$, $\mathtt{i},\mathtt{j} \in \mathtt{I}_N$; the result is a function of ξ;
- $\mathscr{D}^{\mathtt{i},\mathtt{j}}[\overline{q}_t]$ is a function of ξ;
- $\mathscr{C}^{\mathtt{i},\mathtt{j}}[\overline{q}_t]$ and $\mathscr{D}^{\mathtt{i},\mathtt{j}}[\overline{q}_t]$ depend on all $q^{\mathtt{i}}_t$, $\mathtt{i} \in \mathtt{I}_N$, in general, *nonlinearly.*

When $\mathtt{i},\mathtt{j}$ run over $\mathtt{I}_N$, the system of equations (1.30) can be read as a linear nonhomogeneous system of (ordinary) differential equation (considered independently for each value of ξ). Since all $q^{\mathtt{i}}_t$, $\mathtt{i} \in \mathtt{I}_N$, are known, one can solve (1.30) explicitly.

2 Spatiotemporal characteristics

2.1 Spatiotemporal correlations

By (1.13), spatial correlation function $k_t^{\mathtt{i}_1,\ldots,\mathtt{i}_n}(x_1,\ldots,x_n)$ characterizes the probability to find n agents of types $\mathtt{i}_1,\ldots,\mathtt{i}_n$ of the configuration γ_t in vicinities of positions $x_1,\ldots,x_n\in\mathbb{R}^d$, respectively. It is naturally also important to characterize the similar probability when agents appear in those vicinities at different moments of time.

We restrict ourselves to two moments of time only: $t\geq 0$ and $t+\Delta t$ for some $\Delta t\geq 0$. Moreover, we consider the second order spatiotemporal correlations only. Namely, we are interested to find, for each $\mathtt{i},\mathtt{j}\in\mathtt{I}_N$, a function $k_{t,\Delta t}^{\mathtt{i},\mathtt{j}}(x_1,x_2)\geq 0$, such that, for each symmetric $g_2(x_1,x_2)\geq 0$,

$$\mathbb{E}\Big[\sum_{\substack{x_1\in\gamma_t^{\mathtt{i}}\\ x_2\in\gamma_{t+\Delta t}^{\mathtt{j}}\\ x_1\neq x_2}} g_2(x_1,x_2)\Big]=\int_{\mathbb{R}^d}\int_{\mathbb{R}^d} g_2(x_1,x_2)k_{t,\Delta t}^{\mathtt{i},\mathtt{j}}(x_1,x_2)\,dx_1dx_2. \tag{2.1}$$

It it worth noting that, as we will see below,

$$k_{t,\Delta t}^{\mathtt{i},\mathtt{j}}(x_1,x_2)\neq k_{t,\Delta t}^{\mathtt{j},\mathtt{i}}(x_2,x_1). \tag{2.2}$$

To obtain $k_{t,\Delta t}^{\mathtt{i},\mathtt{j}}$, we proceed as follows. Recall, we consider dynamics of $\gamma_t=\gamma_t^{s_1}\cup\ldots\cup\gamma_t^{s_N}$, $t\geq 0$, $N\in\mathbb{N}$. For each $\mathtt{i}\in\mathtt{I}_N$, we consider auxiliary dynamics of three configurations $\gamma_{\Delta t}^{\mathtt{i}O},\gamma_{\Delta t}^{\mathtt{i}+},\gamma_{\Delta t}^{\mathtt{i}-}$, which will be created at the moment t and will have 'own local time' $\Delta t\geq 0$. Namely

- $\gamma_{\Delta t}^{\mathtt{i}O}$ contains all agents of the considered system which
  - were present in the system at time t having type $\mathtt{i}$;
  - didn't change their positions nor types within the time interval $[t,t+\Delta t]$, and hence they are still present in the system at time $t+\Delta t$ having type $\mathtt{i}$;
- $\gamma_{\Delta t}^{\mathtt{i}+}$ contains all agents of the considered system which
  - appeared in the system within the time interval $[t,t+\Delta t]$ having type $\mathtt{i}$;
  - didn't change their positions nor types after that, and hence they are still present at the system at time $t+\Delta t$ having type $\mathtt{i}$;
- $\gamma_{\Delta t}^{\mathtt{i}-}$ contains all agents of the considered system which
  - were present in the system at time t having type $\mathtt{i}$;
  - do not present in the system at time $t+\Delta t$: namely, each of such agent, within the time interval $[t,t+\Delta t]$, either disappeared from the system or changed its position and/or type.

We have hence

$$\begin{aligned} \gamma_0^{\mathtt{i}O} &:= \gamma_{\Delta t}^{\mathtt{i}O}\Big|_{\Delta t=0} = \gamma_t; \\ \gamma_0^{\mathtt{i}+} := \gamma_{\Delta t}^{\mathtt{i}+}\Big|_{\Delta t=0} = \varnothing; &\qquad \gamma_0^{\mathtt{i}-} := \gamma_{\Delta t}^{\mathtt{i}-}\Big|_{\Delta t=0} = \varnothing; \end{aligned} \tag{2.3}$$

and

$$\gamma_t = \gamma_{\Delta t}^{\mathtt{i}O} \cup \gamma_{\Delta t}^{\mathtt{i}-}, \qquad \gamma_{t+\Delta t} = \gamma_{\Delta t}^{\mathtt{i}O} \cup \gamma_{\Delta t}^{\mathtt{i}+}. \tag{2.4}$$

Therefore, our full auxiliary dynamics is

$$\overline{\gamma}_{\Delta t} = \big(\gamma_{\Delta t}^{s_1 O}, \gamma_{\Delta t}^{s_1+}, \gamma_{\Delta t}^{s_1-}, \ldots, \gamma_{\Delta t}^{s_N O}, \gamma_{\Delta t}^{s_N+}, \gamma_{\Delta t}^{s_N-}\big), \tag{2.5}$$

i.e. it contains agents of $3N$ types (recall that a type is just a label). We set

$$\begin{aligned} \mathtt{I}_N^O &:= \{s_1 O, \ldots, s_N O\}, & \mathtt{I}_N^+ &:= \{s_1+, \ldots, s_N+\}, \\ \mathtt{I}_N^- &:= \{s_1-, \ldots, s_N-\}, & \overline{\mathtt{I}}_N &:= \mathtt{I}_N^O \cup \mathtt{I}_N^+ \cup \mathtt{I}_N^-. \end{aligned} \tag{2.6}$$

Recall also that there are two notations to represent configurations, see (1.1), hence, we can also write

$$\overline{\gamma}_{\Delta t} = (\gamma_{\Delta t}^O, \gamma_{\Delta t}^+, \gamma_{\Delta t}^-), \tag{2.7}$$

where, for $A \in \{O, +, -\}$,

$$\gamma_{\Delta t}^A := (\gamma_{\Delta t}^{s_1 A}, \ldots, \gamma_{\Delta t}^{s_N A}) = \gamma_{\Delta t}^{s_1 A} \cup \ldots \cup \gamma_{\Delta t}^{s_N A}.$$

Next, by (2.4), we can rewrite the l.h.s. of (2.1) as follows

$$\begin{aligned} \mathbb{E}\Big[\sum_{\substack{x_1 \in \gamma_t^{\mathtt{i}} \\ x_2 \in \gamma_{t+\Delta t}^{\mathtt{j}} \\ x_1 \neq x_2}} g_2(x_1, x_2) \Big] &= \mathbb{E}\Big[\sum_{\substack{x_1 \in \gamma_{\Delta t}^{\mathtt{i}O} \cup \gamma_{\Delta t}^{\mathtt{i}-} \\ x_2 \in \gamma_{\Delta t}^{\mathtt{j}O} \cup \gamma_{\Delta t}^{\mathtt{j}+} \\ x_1 \neq x_2}} g_2(x_1, x_2) \Big] \\ = \mathbb{E}\Big[\sum_{\substack{x_1 \in \gamma_{\Delta t}^{\mathtt{i}O} \\ x_2 \in \gamma_{\Delta t}^{\mathtt{j}O} \\ x_1 \neq x_2}} g_2(x_1, x_2) \Big] &+ \mathbb{E}\Big[\sum_{\substack{x_1 \in \gamma_{\Delta t}^{\mathtt{i}O} \\ x_2 \in \gamma_{\Delta t}^{\mathtt{j}+}}} g_2(x_1, x_2) \Big] \\ + \mathbb{E}\Big[\sum_{\substack{x_1 \in \gamma_{\Delta t}^{\mathtt{i}-} \\ x_2 \in \gamma_{\Delta t}^{\mathtt{j}O}}} g_2(x_1, x_2) \Big] &+ \mathbb{E}\Big[\sum_{\substack{x_1 \in \gamma_{\Delta t}^{\mathtt{i}-} \\ x_2 \in \gamma_{\Delta t}^{\mathtt{j}+}}} g_2(x_1, x_2) \Big] \end{aligned}$$

and using (1.7) and Remark 1.3 for (2.5), one can continue

$$\begin{aligned} = \int_{\mathbb{R}^d} \int_{\mathbb{R}^d} g_2(x_1, x_2) \Big[& k_{\Delta t}^{\mathtt{i}O, \mathtt{j}O}(x_1, x_2) + k_{\Delta t}^{\mathtt{i}O, \mathtt{j}+}(x_1, x_2) \\ & + k_{\Delta t}^{\mathtt{i}-, \mathtt{j}O}(x_1, x_2) + k_{\Delta t}^{\mathtt{i}-, \mathtt{j}+}(x_1, x_2) \Big] \, dx_1 dx_2. \end{aligned}$$

Therefore, by (2.1),

$$k^{\mathtt{i},\mathtt{j}}_{t,\Delta t}(x_1,x_2) = k^{\mathtt{i}O,\mathtt{j}O}_{\Delta t}(x_1,x_2) + k^{\mathtt{i}O,\mathtt{j}+}_{\Delta t}(x_1,x_2) + k^{\mathtt{i}-,\mathtt{j}O}_{\Delta t}(x_1,x_2) + k^{\mathtt{i}-,\mathtt{j}+}_{\Delta t}(x_1,x_2). \tag{2.8}$$

Formula (2.8) expresses *second order spatiotemporal correlation function* $k^{\mathtt{i},\mathtt{j}}_{t,\Delta t}$ through second order spatial correlation functions $k^{\mathtt{i}A,\mathtt{j}B}_{\Delta t}$ for $A, B \in \{O, +, -\}$.

Remark 2.1. Note that, by Definition 1.2,

$$k^{\mathtt{i}A,\mathtt{j}B}_{\Delta t}(x_1,x_2) = k^{\mathtt{j}B,\mathtt{i}A}_{\Delta t}(x_2,x_1), \qquad A, B \in \{O,+,-\},$$

however, by (2.8), in general, (2.2) holds.

Note that,

$$\sum_{\substack{x_1\in\gamma^{\mathtt{i}}_t\\ x_2\in\gamma^{\mathtt{j}}_{t+\Delta t}}} g_2(x_1,x_2) = \sum_{\substack{x_1\in\gamma^{\mathtt{i}}_t\\ x_2\in\gamma^{\mathtt{j}}_{t+\Delta t}\\ x_1\neq x_2}} g_2(x_1,x_2) + \sum_{x\in\gamma^{\mathtt{i}}_t\cap\gamma^{\mathtt{j}}_{t+\Delta t}} g_2(x,x),$$

and

$$\gamma^{\mathtt{i}}_t \cap \gamma^{\mathtt{j}}_{t+\Delta t} = (\gamma^{\mathtt{i}O}_{\Delta t} \cup \gamma^{\mathtt{i}-}_{\Delta t}) \cap (\gamma^{\mathtt{j}O}_{\Delta t} \cup \gamma^{\mathtt{j}+}_{\Delta t}) = \begin{cases} \gamma^{\mathtt{i}O}_{\Delta t}, & \mathtt{i}=\mathtt{j},\\ \varnothing, & \text{otherwise}.\end{cases}$$

As a result, cf. (1.8),

$$\mathbb{E}\Big[\sum_{\substack{x_1\in\gamma^{\mathtt{i}}_t\\ x_2\in\gamma^{\mathtt{j}}_{t+\Delta t}}} g_2(x_1,x_2)\Big] = \mathbb{E}\Big[\sum_{\substack{x_1\in\gamma^{\mathtt{i}}_t\\ x_2\in\gamma^{\mathtt{j}}_{t+\Delta t}\\ x_1\neq x_2}} g_2(x_1,x_2)\Big] + 1\!\!1_{\mathtt{i}=\mathtt{j}}\,\mathbb{E}\Big[\sum_{x\in\gamma^{\mathtt{i}O}_{\Delta t}} g_2(x,x)\Big]$$

$$= \int_{\mathbb{R}^d}\int_{\mathbb{R}^d} g_2(x_1,x_2) k^{\mathtt{i},\mathtt{j}}_{t,\Delta t}(x_1,x_2)\,dx_1dx_2 + 1\!\!1_{\mathtt{i}=\mathtt{j}} \int_{\mathbb{R}^d} g_2(x,x) k^{\mathtt{i}O}_{\Delta t}(x)\,dx, \tag{2.9}$$

where $k^{\mathtt{i},\mathtt{j}}_{t,\Delta t}$ satisfies (2.8).

Substituting g_2 of the form (1.9) into (2.9), we get, cf. (1.10),

$$\mathbb{E}\Big[|\gamma^{\mathtt{i}}_t\cap\Lambda_1|\,|\gamma^{\mathtt{j}}_{t+\Delta t}\cap\Lambda_2|\Big] = \int_{\Lambda_1}\int_{\Lambda_2} k^{\mathtt{i},\mathtt{j}}_{t,\Delta t}(x_1,x_2)\,dx_1dx_2 + 1\!\!1_{\mathtt{i}=\mathtt{j}} \int_{\Lambda_1\cap\Lambda_2} k^{\mathtt{i}O}_{\Delta t}(x)\,dx. \tag{2.10}$$

By an analogy to (1.12), one can consider also *the centralized spatiotemporal second moment*:

$$\mathbb{E}\Big[\Big(|\gamma^{\mathtt{i}}_t\cap\Lambda_1| - \mathbb{E}\big[|\gamma^{\mathtt{i}}_t\cap\Lambda_1|\big]\Big)\Big(|\gamma^{\mathtt{j}}_{t+\Delta t}\cap\Lambda_2| - \mathbb{E}\big[|\gamma^{\mathtt{j}}_{t+\Delta t}\cap\Lambda_2|\big]\Big)\Big]$$

$$= \mathbb{E}\Big[|\gamma^{\mathtt{i}}_t\cap\Lambda_1|\,|\gamma^{\mathtt{j}}_{t+\Delta t}\cap\Lambda_2|\Big] - \mathbb{E}\Big[|\gamma^{\mathtt{i}}_t\cap\Lambda_1|\Big]\,\mathbb{E}\Big[|\gamma^{\mathtt{j}}_{t+\Delta t}\cap\Lambda_2|\Big]. \tag{2.11}$$

To calculate it, note that, for each $g_1(x) \geq 0$, we have, by (2.4):

$$\mathbb{E}\Big[\sum_{x\in\gamma_t^{\mathtt{i}}} g_1(x)\Big] = \mathbb{E}\Big[\sum_{x\in\gamma_{\Delta t}^{\mathtt{i}O}} g_1(x)\Big] + \mathbb{E}\Big[\sum_{x\in\gamma_{\Delta t}^{\mathtt{i}-}} g_1(x)\Big],$$
$$\mathbb{E}\Big[\sum_{x\in\gamma_{t+\Delta t}^{\mathtt{i}}} g_1(x)\Big] = \mathbb{E}\Big[\sum_{x\in\gamma_{\Delta t}^{\mathtt{i}O}} g_1(x)\Big] + \mathbb{E}\Big[\sum_{x\in\gamma_{\Delta t}^{\mathtt{i}+}} g_1(x)\Big],$$

and therefore,

$$\begin{aligned} k_t^{\mathtt{i}}(x) &= k_{\Delta t}^{\mathtt{i}O}(x) + k_{\Delta t}^{\mathtt{i}-}(x), \\ k_{t+\Delta t}^{\mathtt{i}}(x) &= k_{\Delta t}^{\mathtt{i}O}(x) + k_{\Delta t}^{\mathtt{i}+}(x). \end{aligned} \tag{2.12}$$

Hence,

$$\begin{aligned} \mathbb{E}\Big[|\gamma_t^{\mathtt{i}} \cap \Lambda_1|\Big] &= \int_\Lambda k_{\Delta t}^{\mathtt{i}O}(x)\,dx + \int_\Lambda k_{\Delta t}^{\mathtt{i}-}(x)\,dx, \\ \mathbb{E}\Big[|\gamma_{t+\Delta t}^{\mathtt{j}} \cap \Lambda_1|\Big] &= \int_\Lambda k_{\Delta t}^{\mathtt{j}O}(x)\,dx + \int_\Lambda k_{\Delta t}^{\mathtt{j}+}(x)\,dx. \end{aligned} \tag{2.13}$$

Substituting (2.10) and (2.13) into (2.11), we get

$$\begin{aligned} &\mathbb{E}\Big[\Big(|\gamma_t^{\mathtt{i}} \cap \Lambda_1| - \mathbb{E}\big[|\gamma_t^{\mathtt{i}} \cap \Lambda_1|\big]\Big)\Big(|\gamma_{t+\Delta t}^{\mathtt{j}} \cap \Lambda_2| - \mathbb{E}\big[|\gamma_{t+\Delta t}^{\mathtt{j}} \cap \Lambda_2|\big]\Big)\Big] \\ &\quad = \int_{\Lambda_1}\int_{\Lambda_2} u_{t,\Delta t}^{\mathtt{i},\mathtt{j}}(x_1,x_2)\,dx_1 dx_2 + 1\!\!1_{\mathtt{i}=\mathtt{j}} \int_{\Lambda_1\cap\Lambda_2} k_{\Delta t}^{\mathtt{i}O}(x)\,dx, \end{aligned} \tag{2.14}$$

where, by (2.8), (2.12)

$$u_{t,\Delta t}^{\mathtt{i},\mathtt{j}}(x_1,x_2) = k_{t,\Delta t}^{\mathtt{i},\mathtt{j}}(x_1,x_2) - k_t^{\mathtt{i}}(x_1)k_{t+\Delta t}^{\mathtt{j}}(x_2) \tag{2.15}$$
$$\begin{aligned} &= k_{t,\Delta t}^{\mathtt{i},\mathtt{j}}(x_1,x_2) - \big(k_{\Delta t}^{\mathtt{i}O}(x_1) + k_{\Delta t}^{\mathtt{i}-}(x_1)\big)\big(k_{\Delta t}^{\mathtt{j}O}(x_2) + k_{\Delta t}^{\mathtt{j}+}(x_2)\big) \\ &= k_{\Delta t}^{\mathtt{i}O,\mathtt{j}O}(x_1,x_2) + k_{\Delta t}^{\mathtt{i}O,\mathtt{j}+}(x_1,x_2) + k_{\Delta t}^{\mathtt{i}-,\mathtt{j}O}(x_1,x_2) \\ &\quad + k_{\Delta t}^{\mathtt{i}-,\mathtt{j}+}(x_1,x_2) - k_{\Delta t}^{\mathtt{i}O}(x_1)k_{\Delta t}^{\mathtt{j}O}(x_2) - k_{\Delta t}^{\mathtt{i}O}(x_1)k_{\Delta t}^{\mathtt{j}+}(x_2) \\ &\quad - k_{\Delta t}^{\mathtt{i}-}(x_1)k_{\Delta t}^{\mathtt{j}O}(x_2) - k_{\Delta t}^{\mathtt{i}-}(x_1)k_{\Delta t}^{\mathtt{j}+}(x_2). \end{aligned} \tag{2.16}$$

Combining (2.16) with (1.11), we get

$$\begin{aligned} u_{t,\Delta t}^{\mathtt{i},\mathtt{j}}(x_1,x_2) &= u_{\Delta t}^{\mathtt{i}O,\mathtt{j}O}(x_1,x_2) + u_{\Delta t}^{\mathtt{i}O,\mathtt{j}+}(x_1,x_2) \\ &\quad + u_{\Delta t}^{\mathtt{i}-,\mathtt{j}O}(x_1,x_2) + u_{\Delta t}^{\mathtt{i}-,\mathtt{j}+}(x_1,x_2). \end{aligned} \tag{2.17}$$

Similarly to the noted in Remark 2.1,

$$u_{t,\Delta t}^{\mathtt{i},\mathtt{j}}(x_1,x_2) \neq u_{t,\Delta t}^{\mathtt{j},\mathtt{i}}(x_2,x_1).$$

2.2 Dynamics of the auxiliary model

To study the dynamics of $\overline{\gamma}_{\Delta t}$, we need to consider a modification of (1.5):

$$\frac{d}{d\Delta t}\mathbb{E}\Big[F(\overline{\gamma}_{\Delta t})\Big] = \mathbb{E}\Big[(\overline{L}F)(\overline{\gamma}_{\Delta t})\Big], \tag{2.18}$$

with an appropriate modification $\overline{L}$ of L given by (1.4). Namely, we need that

- a reactant $x^{\mathtt{i}O}$ does not just disappear from $\gamma^O_{\Delta t}$, but changes its type becoming $x^{\mathtt{i}-} \in \gamma^-_{\Delta t}$;
- a reactant $x^{\mathtt{i}+}$ just disappears from $\gamma^+_{\Delta t}$ (since then this agent did not exist at time t and will not exist at time $t + \Delta t$);
- products may be of the type '+' only; a product $z^{\mathtt{i}+}$ just appears in $\gamma^{\mathtt{i}+}_{\Delta t}$;
- the event should happen with the same rate as for L, but applied to the union of O-catalysts and +-catalysts (as they all present at the system on the time interval $[t, t + \Delta t]$);
- agents of the type '−' do not perform own dynamics (hence they appear because of the transformation from O-reactants only).

Let $\mathtt{R}, \mathtt{C}, \mathtt{P}$ be fixed and $L_{\mathtt{R},\mathtt{C},\mathtt{P}}$ be given by (1.3). It means that there are still $\mathtt{R}$ reactants, some of them are O-reactants (denote their number by $\mathtt{r}$, so that $0 \leq \mathtt{r} \leq \mathtt{R}$), the rest are +-reactants, namely, there are $\mathtt{R} - \mathtt{r} \geq 0$ of +-reactants. Next, there are $\mathtt{P}$ products, all are +-products by the above. Finally, $\mathtt{C}$ catalysts should be chosen from $\gamma^O_{\Delta t} \cup \gamma^+_{\Delta t}$; let, similarly to reactants, there be $\mathtt{c}$ O-catalysts $(0 \leq \mathtt{c} \leq \mathtt{C})$ and hence $\mathtt{C} - \mathtt{c} \geq 0$ +-catalysts.

As a result, we will get

$$\begin{aligned}
&(\overline{L}_{\mathtt{R},\mathtt{C},\mathtt{P}}F)(\gamma^O, \gamma^+, \gamma^-)\\
&= \sum_{\mathtt{r}=0}^{\mathtt{R}} \sum_{\mathtt{c}=0}^{\mathtt{C}} \sum_{\{x_1^{\mathtt{i}_1 O},\dots,x_{\mathtt{r}}^{\mathtt{i}_{\mathtt{r}} O}\}\subset\gamma^O} \sum_{\{x_1^{\mathtt{i}_1 +},\dots,x_{\mathtt{R}-\mathtt{r}}^{\mathtt{i}_{\mathtt{R}-\mathtt{r}} +}\}\subset\gamma^+}\\
&\quad \sum_{\{y_1^{\mathtt{j}_1 O},\dots,y_{\mathtt{c}}^{\mathtt{j}_{\mathtt{c}} O}\}\subset\gamma^O\setminus\{x_1^{\mathtt{i}_1 O},\dots,x_{\mathtt{r}}^{\mathtt{i}_{\mathtt{r}} O}\}} \quad \sum_{\{y_1^{\mathtt{j}_1 +},\dots,y_{\mathtt{C}-\mathtt{c}}^{\mathtt{j}_{\mathtt{C}-\mathtt{c}} +}\}\subset\gamma^+\setminus\{x_1^{\mathtt{i}_1 +},\dots,x_{\mathtt{R}-\mathtt{r}}^{\mathtt{i}_{\mathtt{R}-\mathtt{r}} +}\}}\\
&\quad \times \int_{(\mathbb{R}^d)^{\mathtt{P}}} r\Big(x_1^{\mathtt{i}_1 O},\dots,x_{\mathtt{r}}^{\mathtt{i}_{\mathtt{r}} O}, x_1^{\mathtt{i}_1 +},\dots,x_{\mathtt{R}-\mathtt{r}}^{\mathtt{i}_{\mathtt{R}-\mathtt{r}} +},\\
&\qquad\qquad y_1^{\mathtt{j}_1 O},\dots,y_{\mathtt{c}}^{\mathtt{j}_{\mathtt{c}} O}, y_1^{\mathtt{j}_1 +},\dots,y_{\mathtt{C}-\mathtt{c}}^{\mathtt{j}_{\mathtt{C}-\mathtt{c}} +}, z_1^{\mathtt{l}_1 +},\dots,z_{\mathtt{P}}^{\mathtt{l}_{\mathtt{P}} +}\Big)\\
&\quad \times \bigg(F\Big(\gamma^O \setminus \{x_1^{\mathtt{i}_1 O},\dots,x_{\mathtt{r}}^{\mathtt{i}_{\mathtt{r}} O}\},\\
&\qquad\qquad \gamma^+ \setminus \{x_1^{\mathtt{i}_1 +},\dots,x_{\mathtt{R}-\mathtt{r}}^{\mathtt{i}_{\mathtt{R}-\mathtt{r}} +}\} \cup \{z_1^{\mathtt{l}_1 +},\dots,z_{\mathtt{P}}^{\mathtt{l}_{\mathtt{P}} +}\},\\
&\qquad\qquad\quad \gamma^- \cup \{x_1^{\mathtt{i}_1 -},\dots,x_{\mathtt{r}}^{\mathtt{i}_{\mathtt{r}} -}\}\Big) - F(\gamma)\bigg)\, dz_1 \dots dz_{\mathtt{P}}.
\end{aligned}$$

Naturally, we also set

$$\overline{L} = \sum_{\substack{\mathtt{R},\mathtt{C},\mathtt{P}\geq 0\\ \mathtt{R}+\mathtt{P}>0}} \overline{L}_{\mathtt{R},\mathtt{C},\mathtt{P}}.$$

Remark 2.2. It may be also convenient to use another style of writing. Namely, we will interpret now $\overline{\gamma}_{\Delta t}$ as the union:

$$\overline{\gamma}_{\Delta t} = \gamma^O_{\Delta t} \cup \gamma^+_{\Delta t} \cup \gamma^-_{\Delta t},$$

rather than as the the tuple (2.7). Then, for

$$\mathtt{i}_1, \ldots, \mathtt{i}_\mathtt{R}, \mathtt{j}_1, \ldots, \mathtt{j}_\mathtt{C}, \mathtt{l}_1, \ldots, \mathtt{l}_\mathtt{P} \in \overline{\mathtt{I}}_N,$$

we have

$$\begin{aligned}(\overline{L}_{\mathtt{R},\mathtt{C},\mathtt{P}}F)(\overline{\gamma}) = &\sum_{\{x_1^{\mathtt{i}_1},\ldots,x_\mathtt{R}^{\mathtt{i}_\mathtt{R}}\}\subset\overline{\gamma}} \quad \sum_{\{y_1^{\mathtt{j}_1},\ldots,y_\mathtt{C}^{\mathtt{j}_\mathtt{C}}\}\subset\overline{\gamma}\setminus\{x_1^{\mathtt{i}_1},\ldots,x_\mathtt{R}^{\mathtt{i}_\mathtt{R}}\}} \\ &\int_{(\mathbb{R}^d)^\mathtt{P}} \overline{r}(x_1^{\mathtt{i}_1}, \ldots, x_\mathtt{R}^{\mathtt{i}_\mathtt{R}}, y_1^{\mathtt{j}_1}, \ldots, y_\mathtt{C}^{\mathtt{j}_\mathtt{C}}, z_1^{\mathtt{l}_1}, \ldots, z_\mathtt{P}^{\mathtt{l}_\mathtt{P}}) \\ &\times \Big(F\big(\overline{\gamma} \setminus \{x_1^{\mathtt{i}_1}, \ldots, x_\mathtt{R}^{\mathtt{i}_\mathtt{R}}\} \cup \{z_1^{\mathtt{l}_1}, \ldots, z_\mathtt{P}^{\mathtt{l}_\mathtt{P}}\}\big) - F\big(\overline{\gamma}\big)\Big)\, dz_1 \ldots dz_\mathtt{P},\end{aligned}$$

where

$$\begin{aligned}&\overline{r}(x_1^{\mathtt{i}_1}, \ldots, x_\mathtt{R}^{\mathtt{i}_\mathtt{R}}, y_1^{\mathtt{j}_1}, \ldots, y_\mathtt{C}^{\mathtt{j}_\mathtt{C}}, z_1^{\mathtt{l}_1}, \ldots, z_\mathtt{P}^{\mathtt{l}_\mathtt{P}}) \\ &= \prod_{m=1}^{\mathtt{R}} 1\!\!1_{\mathtt{i}_m\in\mathtt{I}_N^O\cup\mathtt{I}_N^+} \prod_{m=1}^{\mathtt{C}} 1\!\!1_{\mathtt{j}_m\in\mathtt{I}_N^O\cup\mathtt{I}_N^+} \prod_{m=1}^{\mathtt{P}} 1\!\!1_{\mathtt{l}_m\in\mathtt{I}_N^+\cup\mathtt{I}_N^-} \\ &\times 1\!\!1_{\text{for each } \mathtt{i}\in\mathtt{I}_N^O \text{ there is a unique } \mathtt{l}\in\mathtt{I}_N^-}\ \delta(x^\mathtt{i} - z^\mathtt{l}) \\ &\times r(x_1^{\mathtt{i}_1}, \ldots, x_\mathtt{R}^{\mathtt{i}_\mathtt{R}}, y_1^{\mathtt{j}_1}, \ldots, y_\mathtt{C}^{\mathtt{j}_\mathtt{C}}, z_1^{\mathtt{l}_1}, \ldots, z_\mathtt{P}^{\mathtt{l}_\mathtt{P}}).\end{aligned}$$

2.3 Beyond mean-field expansion for spatiotemporal dynamics

Let L_ε be given by (1.4) with $r_{\mathtt{R},\mathtt{C},\mathtt{P}}$ in (1.3) replaced by (1.19) as it is described in Subsection 1.3. Let, initially, $\gamma_{\varepsilon,0}$ be distributed so that the corresponding correlation functions has the form (1.20) for certain fixed collection of functions $q_0^\mathtt{i}$, $\mathtt{i} \in \mathtt{I}_N$. Let $\gamma_{\varepsilon,t}$ and $\gamma_{\varepsilon,t+\Delta t}$ be the corresponding random configurations at times t and $t + \Delta t$, respectively.

We consider the corresponding auxiliary dynamics

$$\overline{\gamma}_{\varepsilon,\Delta t} = (\gamma^O_{\varepsilon,\Delta t}, \gamma^+_{\varepsilon,\Delta t}, \gamma^-_{\varepsilon,\Delta t}),$$

distributed according to the generator $\overline{L}_\varepsilon$ obtained from L_ε by an analogy to that done in Subsection 2.2, in particular, by (2.3),

$$\gamma^O_{\varepsilon,0} = \gamma_{\varepsilon,t}, \qquad \gamma^+_{\varepsilon,0} = \gamma^-_{\varepsilon,0} = \varnothing. \tag{2.19}$$

Let $k_{\varepsilon,t}^{\widehat{\mathtt{i}}_1,\ldots,\widehat{\mathtt{i}}_n}(x_1,\ldots,x_n)$, $\widehat{\mathtt{i}}_1,\ldots,\widehat{\mathtt{i}}_n \in \overline{\mathtt{I}}_N$, be the corresponding system of correlation functions.

By (2.19) and (1.21), we have:

- for each $\mathtt{i}_1,\ldots,\mathtt{i}_n \in \mathtt{I}_N$,
$$k_{\varepsilon,0}^{\mathtt{i}_1 O,\ldots,\mathtt{i}_n O}(x_1,\ldots,x_n) = k_{\varepsilon,t}^{\mathtt{i}_1,\ldots,\mathtt{i}_n}(x_1,\ldots,x_n) = q_t^{\mathtt{i}_1}(\varepsilon x_1)\ldots q_t^{\mathtt{i}_n}(\varepsilon x_1) + o(1);$$
- if $\widehat{\mathtt{i}}_1,\ldots,\widehat{\mathtt{i}}_n \in \overline{\mathtt{I}}_N$, cf. (2.6), are such that $\widehat{\mathtt{i}}_m \in \mathtt{I}_N^+ \cup \mathtt{I}_N^-$ for at least one $1\leq m\leq n$, then
$$k_{\varepsilon,0}^{\widehat{\mathtt{i}}_1,\ldots,\widehat{\mathtt{i}}_n}(x_1,\ldots,x_n) = 0.$$

Hence, one can define, for each $\mathtt{i}\in\mathtt{I}_N$,

$$q_0^{\mathtt{i}O}(x) := q_t^{\mathtt{i}}(x), \qquad q_0^{\mathtt{i}+}(x) := q_0^{\mathtt{i}-}(x) := 0, \tag{2.20}$$

and then, for each $\widehat{\mathtt{i}}_1,\ldots,\widehat{\mathtt{i}}_n \in \overline{\mathtt{I}}_N$, we will get that

$$k_{\varepsilon,0}^{\widehat{\mathtt{i}}_1,\ldots,\widehat{\mathtt{i}}_n}(x_1,\ldots,x_n) = q_0^{\widehat{\mathtt{i}}_1}(\varepsilon x_1)\ldots q_0^{\widehat{\mathtt{i}}_n}(\varepsilon x_1) + o(1),$$

that is an analogue to (1.20) to have the needed settings for the auxiliary dynamics.

Applying (1.24) for the auxiliary dynamics, we get:

$$k_{\varepsilon,\Delta t}^{\mathtt{i}A}(x) = q_{\Delta t}^{\mathtt{i}A}(\varepsilon x) + \varepsilon^d p_{\Delta t}^{\mathtt{i}A}(\varepsilon x) + o(1), \qquad A\in\{O,+,-\}. \tag{2.21}$$

Taking $\Delta t = 0$ in (2.21) and using that, by (1.24),

$$\begin{aligned} k_{\varepsilon,0}^{\mathtt{i}O}(x) &= k_{\varepsilon,t}^{\mathtt{i}}(x) = q_t^{\mathtt{i}}(\varepsilon x) + \varepsilon^d p_t^{\mathtt{i}}(\varepsilon x) + o(1),\\ k_{\varepsilon,0}^{\mathtt{i}+}(x) &= k_{\varepsilon,0}^{\mathtt{i}-}(x) = 0, \end{aligned}$$

we conclude, cf. (2.20),

$$p_0^{\mathtt{i}O}(x) = p_t^{\mathtt{i}}(x), \qquad p_0^{\mathtt{i}+}(x) = p_0^{\mathtt{i}-}(x) = 0.$$

Rewriting (2.12), one gets then

$$\begin{aligned} k_{\varepsilon,t}^{\mathtt{i}}(x) &= k_{\varepsilon,\Delta t}^{\mathtt{i}O}(x) + k_{\varepsilon,\Delta t}^{\mathtt{i}-}(x),\\ k_{\varepsilon,t+\Delta t}^{\mathtt{i}}(x) &= k_{\varepsilon,\Delta t}^{\mathtt{i}O}(x) + k_{\varepsilon,\Delta t}^{\mathtt{i}+}(x). \end{aligned} \tag{2.22}$$

Then, applying expansions (1.24) and (2.21) to left and right sides of (2.22), respectively, and equating the corresponding coefficients, one gets

$$q_t^{\mathtt{i}}(x) = q_{\Delta t}^{\mathtt{i}O}(x) + q_{\Delta t}^{\mathtt{i}-}(x), \qquad p_t^{\mathtt{i}}(x) = p_{\Delta t}^{\mathtt{i}O}(x) + p_{\Delta t}^{\mathtt{i}-}(x), \tag{2.23}$$

$$q_{t+\Delta t}^{\mathtt{i}}(x) = q_{\Delta t}^{\mathtt{i}O}(x) + q_{\Delta t}^{\mathtt{i}+}(x), \qquad p_{t+\Delta t}^{\mathtt{i}}(x) = p_{\Delta t}^{\mathtt{i}O}(x) + p_{\Delta t}^{\mathtt{i}+}(x). \tag{2.24}$$

Consider now functions $k^{\mathtt{i},\mathtt{j}}_{\varepsilon,t,\Delta t}$, $\mathtt{i},\mathtt{j}\in\mathtt{I}_N$, defined by an analogy to (2.1), namely, for all symmetric $g_2(x_1,x_2)\ge 0$,

$$\mathbb{E}\Big[\sum_{\substack{x_1\in\gamma^{\mathtt{i}}_{\varepsilon,t}\\ x_2\in\gamma^{\mathtt{j}}_{\varepsilon,t+\Delta t}\\ x_1\neq x_2}} g_2(x_1,x_2)\Big]=\int_{\mathbb{R}^d}\int_{\mathbb{R}^d} g_2(x_1,x_2)k^{\mathtt{i},\mathtt{j}}_{\varepsilon,t,\Delta t}(x_1,x_2)\,dx_1dx_2.$$

We have then, by (2.8),

$$\begin{aligned}k^{\mathtt{i},\mathtt{j}}_{\varepsilon,t,\Delta t}(x_1,x_2)&=k^{\mathtt{i}O,\mathtt{j}O}_{\varepsilon,\Delta t}(x_1,x_2)+k^{\mathtt{i}O,\mathtt{j}+}_{\varepsilon,\Delta t}(x_1,x_2)\\&\quad+k^{\mathtt{i}-,\mathtt{j}O}_{\varepsilon,\Delta t}(x_1,x_2)+k^{\mathtt{i}-,\mathtt{j}+}_{\varepsilon,\Delta t}(x_1,x_2).\end{aligned}$$

By (2.14) and (2.17), we have then

$$\begin{aligned}&\mathbb{E}\Big[\Big(|\gamma^{\mathtt{i}}_{\varepsilon,t}\cap\Lambda_1|-\mathbb{E}\big[|\gamma^{\mathtt{i}}_{\varepsilon,t}\cap\Lambda_1|\big]\Big)\Big(|\gamma^{\mathtt{j}}_{\varepsilon,t+\Delta t}\cap\Lambda_2|-\mathbb{E}\big[|\gamma^{\mathtt{j}}_{\varepsilon,t+\Delta t}\cap\Lambda_2|\big]\Big)\Big]\\&\qquad=\int_{\Lambda_1}\int_{\Lambda_2}u^{\mathtt{i},\mathtt{j}}_{\varepsilon,t,\Delta t}(x_1,x_2)\,dx_1dx_2+1\!\!1_{\mathtt{i}=\mathtt{j}}\int_{\Lambda_1\cap\Lambda_2}k^{\mathtt{i}O}_{\varepsilon,\Delta t}(x)\,dx,\end{aligned}\tag{2.25}$$

where

$$\begin{aligned}u^{\mathtt{i},\mathtt{j}}_{\varepsilon,t,\Delta t}(x_1,x_2)&=u^{\mathtt{i}O,\mathtt{j}O}_{\varepsilon,\Delta t}(x_1,x_2)+u^{\mathtt{i}O,\mathtt{j}+}_{\varepsilon,\Delta t}(x_1,x_2)\\&\quad+u^{\mathtt{i}-,\mathtt{j}O}_{\varepsilon,\Delta t}(x_1,x_2)+u^{\mathtt{i}-,\mathtt{j}+}_{\varepsilon,\Delta t}(x_1,x_2),\end{aligned}\tag{2.26}$$

and, cf. (1.11), for $A,B\in\{O,+,-\}$,

$$u^{\mathtt{i}A,\mathtt{j}B}_{\varepsilon,\Delta t}(x_1,x_2)=k^{\mathtt{i}A,\mathtt{j}B}_{\varepsilon,\Delta t}(x_1,x_2)-k^{\mathtt{i}A}_{\varepsilon,\Delta t}(x_1)k^{\mathtt{j}B}_{\varepsilon,\Delta t}(x_2).\tag{2.27}$$

Applying (1.24) to the auxiliary dynamics, one gets, for each $A,B\in\{O,+,-\}$,

$$u^{\mathtt{i}A,\mathtt{j}B}_{\varepsilon,\Delta t}(x_1,x_2)=\varepsilon^d g^{\mathtt{i}A,\mathtt{j}B}_{\Delta t}(\varepsilon x_1,\varepsilon x_2)+o(1).\tag{2.28}$$

Taking $\Delta t=0$ in (2.28) we have, by (2.27), (1.24), (2.4),

$$\begin{aligned}u^{\mathtt{i}O,\mathtt{j}O}_{\varepsilon,0}(x_1,x_2)&=u^{\mathtt{i},\mathtt{j}}_{\varepsilon,t}(x_1,x_2)=\varepsilon^d g^{\mathtt{i},\mathtt{j}}_t(\varepsilon x_1,\varepsilon x_2)+o(1),\\u^{\mathtt{i}O,\mathtt{j}+}_{\varepsilon,0}(x_1,x_2)&=u^{\mathtt{i}O,\mathtt{j}-}_{\varepsilon,0}(x_1,x_2)=u^{\mathtt{i}+,\mathtt{j}-}_{\varepsilon,0}(x_1,x_2)=0.\end{aligned}$$

Hence,

$$\begin{aligned}&g^{\mathtt{i}O,\mathtt{j}O}_0(x_1,x_2)=g^{\mathtt{i},\mathtt{j}}_t(x_1,x_2),\\&g^{\mathtt{i}O,\mathtt{j}+}_0(x_1,x_2)=g^{\mathtt{i}O,\mathtt{j}-}_0(x_1,x_2)=g^{\mathtt{i}+,\mathtt{j}-}_0(x_1,x_2)=0.\end{aligned}$$

Combining (2.28) with (2.26), we get

$$u^{\mathtt{i},\mathtt{j}}_{\varepsilon,t,\Delta t}(x_1,x_2)=\varepsilon^d\,g^{\mathtt{i},\mathtt{j}}_{t,\Delta t}(\varepsilon x_1,\varepsilon x_2)+o(1),\tag{2.29}$$

where

$$g^{\mathtt{i},\mathtt{j}}_{t,\Delta t}(x_1,x_2) = g^{\mathtt{i}O,\mathtt{j}O}_{\Delta t}(x_1,x_2) + g^{\mathtt{i}O,\mathtt{j}+}_{\Delta t}(x_1,x_2) + g^{\mathtt{i}-,\mathtt{j}O}_{\Delta t}(x_1,x_2) + g^{\mathtt{i}-,\mathtt{j}+}_{\Delta t}(x_1,x_2). \tag{2.30}$$

Also, by an analogue of (2.15), we have

$$k^{\mathtt{i},\mathtt{j}}_{\varepsilon,t,\Delta t}(x_1,x_2) = u^{\mathtt{i},\mathtt{j}}_{\varepsilon,t,\Delta t}(x_1,x_2) + k^{\mathtt{i}}_{\varepsilon,t}(x_1)k^{\mathtt{j}}_{\varepsilon,t+\Delta t}(x_2).$$

Then, using expansions (2.29) and (2.22), we get the following analogue of (1.27):

$$\begin{aligned} k^{\mathtt{i},\mathtt{j}}_{\varepsilon,t,\Delta t}(x_1,x_2) = {} & q^{\mathtt{i}}_t(\varepsilon x_1)q^{\mathtt{j}}_{t+\Delta t}(\varepsilon x_2) + \varepsilon^d g^{\mathtt{i},\mathtt{j}}_{t,\Delta t}(\varepsilon x_1,\varepsilon x_2) \\ & + \varepsilon^d\Big(q^{\mathtt{i}}_t(\varepsilon x_1)\, p^{\mathtt{j}}_{t+\Delta t}(\varepsilon x_2) + p^{\mathtt{i}}_t(\varepsilon x_1)\, q^{\mathtt{j}}_{t+\Delta t}(\varepsilon x_2)\Big) \\ & + o(\varepsilon^d), \end{aligned}$$

where the right hand side can be expressed in terms of $q^{\hat{\mathtt{i}}}_{\Delta t}, q^{\hat{\mathtt{j}}}_{\Delta t}, p^{\hat{\mathtt{i}}}_{\Delta t}, p^{\hat{\mathtt{j}}}_{\Delta t}, g^{\hat{\mathtt{i}},\hat{\mathtt{j}}}_{\Delta t}$, $\hat{\mathtt{i}},\hat{\mathtt{j}} \in \bar{\mathtt{I}}_N$, by using (2.23), (2.24), (2.30).

Recall that, cf. Remark 2.1, for $\mathtt{i},\mathtt{j} \in \mathtt{I}_N$,

$$g^{\mathtt{i}A,\mathtt{j}B}_{\Delta t}(x_1,x_2) = g^{\mathtt{j}B,\mathtt{i}A}_{\Delta t}(x_2,x_1), \qquad A,B \in \{O,+,-\},$$

however,

$$g^{\mathtt{i},\mathtt{j}}_{t,\Delta t}(x_1,x_2) \neq g^{\mathtt{j},\mathtt{i}}_{t,\Delta t}(x_2,x_1).$$

Space-homogeneous case Consider again the special case where (1.28) holds. By (2.3), the auxiliary (and scaled by ε) dynamics will inherit that property as well: for all $\hat{\mathtt{i}},\hat{\mathtt{j}} \in \bar{\mathtt{I}}_N$, $k^{\hat{\mathtt{i}}}_{\varepsilon,\Delta t}$ will not depend on a space coordinate, and $k^{\hat{\mathtt{i}},\hat{\mathtt{j}}}_{\varepsilon,\Delta t}(x)$ (and hence $u^{\hat{\mathtt{i}},\hat{\mathtt{j}}}_{\varepsilon,\Delta t}(x)$, $g^{\hat{\mathtt{i}},\hat{\mathtt{j}}}_{\Delta t}(x)$) will depend on one space coordinate only. Rewriting the formulas above, one gets

$$\begin{aligned} k^{\mathtt{i}}_{\varepsilon,t} &= q^{\mathtt{i}O}_{\Delta t} + q^{\mathtt{i}-}_{\Delta t} + \varepsilon^d\big(p^{\mathtt{i}O}_{\Delta t} + p^{\mathtt{i}-}_{\Delta t}\big) + o(\varepsilon^d); \\ k^{\mathtt{i}}_{\varepsilon,t+\Delta t} &= q^{\mathtt{i}O}_{\Delta t} + q^{\mathtt{i}+}_{\Delta t} + \varepsilon^d\big(p^{\mathtt{i}O}_{\Delta t} + p^{\mathtt{i}+}_{\Delta t}\big) + o(\varepsilon^d); \\ u^{\mathtt{i},\mathtt{j}}_{\varepsilon,t,\Delta t}(x) &= \varepsilon^d g^{\mathtt{i},\mathtt{j}}_{t,\Delta t}(\varepsilon x) + o(\varepsilon^d), \end{aligned} \tag{2.31}$$

where

$$\tilde{g}^{\mathtt{i},\mathtt{j}}_{t,\Delta t}(\xi) = \tilde{g}^{\mathtt{i}O,\mathtt{j}O}_{\Delta t}(\xi) + \tilde{g}^{\mathtt{i}O,\mathtt{j}+}_{\Delta t}(\xi) + \tilde{g}^{\mathtt{i}-,\mathtt{j}O}_{\Delta t}(\xi) + \tilde{g}^{\mathtt{i}-,\mathtt{j}+}_{\Delta t}(\xi), \tag{2.32}$$

and

$$\begin{aligned} q^{\mathtt{i}}_t &= q^{\mathtt{i}O}_{\Delta t} + q^{\mathtt{i}-}_{\Delta t}; & p^{\mathtt{i}}_t &= p^{\mathtt{i}O}_{\Delta t} + p^{\mathtt{i}-}_{\Delta t}; \\ q^{\mathtt{i}}_{t+\Delta t} &= q^{\mathtt{i}O}_{\Delta t} + q^{\mathtt{i}+}_{\Delta t}; & p^{\mathtt{i}}_{t+\Delta t} &= p^{\mathtt{i}O}_{\Delta t} + p^{\mathtt{i}+}_{\Delta t}, \end{aligned} \tag{2.33}$$

where, initially,

$$
\begin{gathered}
q_0^{\mathtt{i}O} = q_t^{\mathtt{i}}; \qquad p_0^{\mathtt{i}O} = p_t^{\mathtt{i}}; \\
q_0^{\mathtt{i}+} = q_0^{\mathtt{i}-} = p_0^{\mathtt{i}+} = p_0^{\mathtt{i}-} = 0;
\end{gathered} \tag{2.34}
$$

$$
\begin{gathered}
\widetilde{g}_0^{\mathtt{i}O,\mathtt{j}O}(\xi) = \widetilde{g}_t^{\mathtt{i},\mathtt{j}}(\xi), \\
\widetilde{g}_0^{\mathtt{i}O,\mathtt{j}+}(\xi) = \widetilde{g}_0^{\mathtt{i}O,\mathtt{j}-}(\xi) = \widetilde{g}_0^{\mathtt{i}+,\mathtt{j}-}(\xi) = 0;
\end{gathered} \tag{2.35}
$$

By using (1.30) with $\mathtt{i},\mathtt{j} \in \mathtt{I}_N$ replaced by $\widehat{\mathtt{i}},\widehat{\mathtt{j}} \in \overline{\mathtt{I}}_N$, we get differential equations for all $\widetilde{g}_{\Delta t}^{\widehat{\mathtt{i}},\widehat{\mathtt{j}}}(\xi) = \widetilde{g}_{\Delta t}^{\mathtt{i}A,\mathtt{j}B}(\xi)$, $A, B \in \{O, +, -\}$, with coefficients dependent on $q_t^{\widehat{\mathtt{i}}}$. Solving the obtained system of differential equations, one can find $\widetilde{g}_{t,\Delta t}^{\mathtt{i},\mathtt{j}}(\xi)$ by (2.32). However, we are interested to simplify the computations by finding a differential equation on $\widetilde{g}_{t,\Delta t}^{\mathtt{i},\mathtt{j}}(\xi)$.

We are going to formulate now a conjecture which can be verified for various models (in particular, for the considered below). We are going to prove it in a forthcoming paper.

To formulate the conjecture, we consider auxiliary functions on $\mathbb{R}^d$:

$$
h_t^{\mathtt{i},\mathtt{j}}(\xi) := \widetilde{g}_t^{\mathtt{i},\mathtt{j}}(\xi) + 1\!\!1_{\mathtt{i}=\mathtt{j}} q_t^{\mathtt{i}}, \qquad \mathtt{i},\mathtt{j} \in \mathtt{I}_N. \tag{2.36}
$$

By (1.30) and (1.22), we get that the vector $\overline{h}_t = (h_t^{\mathtt{i},\mathtt{j}})_{\mathtt{i},\mathtt{j}\in\mathtt{I}_N}$ satisfies a *nonhomogeneous* system of linear differential equations:

$$
\frac{d}{dt} h_t^{\mathtt{i},\mathtt{j}}(\xi) = \mathcal{A}^{\mathtt{i},\mathtt{j}}[\overline{q}_t](\overline{h}_t)(\xi) + \mathcal{B}^{\mathtt{i},\mathtt{j}}[\overline{q}_t](\xi), \tag{2.37}
$$

where, similarly to above, $\mathcal{A}^{\mathtt{i},\mathtt{j}}[\overline{q}_t](\cdot)$ is a multilinear mapping, calculated here at the vector $\overline{h}_t$, and $\mathcal{B}^{\mathtt{i},\mathtt{j}}[\overline{q}_t]$ is a function; both depend on $q_t^{\mathtt{i}}$, $\mathtt{i} \in \mathtt{I}_N$, nonlinearly (in general).

Conjecture. Consider another auxiliary functions on $\mathbb{R}^d$:

$$
h_{t,\Delta t}^{\mathtt{i},\mathtt{j}}(\xi) := \widetilde{g}_{t,\Delta t}^{\mathtt{i},\mathtt{j}}(\xi) + 1\!\!1_{\mathtt{i}=\mathtt{j}} q_{\Delta t}^{\mathtt{i}O}, \qquad \mathtt{i},\mathtt{j} \in \mathtt{I}_N; \tag{2.38}
$$

recall that $q_{\Delta t}^{\mathtt{i}O}$ depends on t. Then the vector $\overline{h}_t = (h_{t,\Delta t}^{\mathtt{i},\mathtt{j}})_{\mathtt{i},\mathtt{j}\in\mathtt{I}_N}$ satisfies a *homogeneous* system of linear differential equations:

$$
\frac{d}{d\Delta t} h_{t,\Delta t}^{\mathtt{i},\mathtt{j}}(\xi) = \mathcal{A}_{\mathrm{mod}}^{\mathtt{i},\mathtt{j}}[\overline{q}_{t+\Delta t}](\overline{h}_{t,\Delta t})(\xi). \tag{2.39}
$$

The system of linear equations (2.39), can be solved in matrix form (or, rather, tensor form, as vector $\overline{h_{t,\Delta t}}$ is two-dimensional). Note that the initial condition to (2.39), when $\Delta t = 0$, can be obtained, by (2.38), (2.33), (2.35), (2.32), as follows:

$$
h_{t,0}^{\mathtt{i},\mathtt{j}}(\xi) = \widetilde{g}_{t,0}^{\mathtt{i},\mathtt{j}}(\xi) + 1\!\!1_{\mathtt{i}=\mathtt{j}} q_0^{\mathtt{i}O} = \widetilde{g}_t^{\mathtt{i},\mathtt{j}}(\xi) + 1\!\!1_{\mathtt{i}=\mathtt{j}} q_t^{\mathtt{i}} = h_t^{\mathtt{i},\mathtt{j}}(\xi). \tag{2.40}
$$

Next, if $\mathtt{i} \neq \mathtt{j}$, one has to find $q_{\Delta t}^{\mathtt{i}O}$ (that can be often done explicitly), and get $\widetilde{g}_{t,\Delta t}^{\mathtt{i},\mathtt{j}}(\xi)$ from (2.38). Finally, one has to take the inverse Fourier transform, to obtain $g_{t,\Delta t}^{\mathtt{i},\mathtt{j}}(x)$; the latter, of course, can be done only numerically. As a result, one gets an approximate value of $u_{\varepsilon,t,\Delta t}^{\mathtt{i},\mathtt{j}}(x)$ from (2.31).

3 Case study 1: Spatial and stochastic logistic model

3.1 Spatial characteristics

We consider agents of one type, i.e. $N = 1$. Let L be given through some of three operators, cf. 1.4:

$$L = L_1 + L_2 + L_3,$$

where

$$(L_1F)(\gamma) = \sum_{x\in\gamma} \int_{\mathbb{R}^d} a^+(x-y)\Big(F\big(\gamma\cup\{y\}\big) - F\big(\gamma\big)\Big)dy;$$

$$(L_2F)(\gamma) = m\sum_{x\in\gamma}\Big(F\big(\gamma\setminus\{x\}\big) - F\big(\gamma\big)\Big);$$

$$(L_3F)(\gamma) = \sum_{x\in\gamma}\sum_{y\in\gamma\setminus\{x\}} a^-(x-y)\Big(F\big(\gamma\setminus\{x\}\big) - F\big(\gamma\big)\Big).$$

Here $m > 0$ is a constant, and $a^\pm(x) \geq 0$ are kernels such that

$$\int_{\mathbb{R}^d} a^\pm(x)\,dx + \sup_{x\in\mathbb{R}^d} a^\pm(x) + \int_{\mathbb{R}^d} |\widetilde{a}^\pm(\xi)|\,d\xi < \infty. \tag{3.1}$$

We denote also

$$A^\pm := \int_{\mathbb{R}^d} a^\pm(x)\,dx. \tag{3.2}$$

We will always assume that $A^\pm > 0$, i.e. it is not the case that $a^\pm(x) = 0$ for almost all (a.a. henceforth) $x \in \mathbb{R}^d$.

Operator L_1 describes that any catalyst at $x \in \gamma$ may create a product at $y \in \mathbb{R}^d$ (send an off-spring to y) according to the dispersion kernel a^+; L_2 describes that any reactant at $x \in \gamma$ may disappear with an density independent mortality m; L_3 describes that any reactant at $x \in \gamma$ may also disappear because of competition with catalysts at $y \in \gamma \setminus \{x\}$ given through the competition kernel a^-.

Following the scheme above, we consider L_ε with $a^\pm(x-y)$ above replaced by $\varepsilon^d a^\pm(\varepsilon x - \varepsilon y)$; next, we consider the dynamics of $\gamma_{\varepsilon,t}$ defined by (1.5) with L replaced by L_ε. Let, cf. Remark 1.7, $k^{(1)}_{\varepsilon,t}(x_1)$ and $k^{(2)}_{\varepsilon,t}(x_1,x_2)$ be the corresponding first- and second-order correlation functions, and let $u^{(1)}_{\varepsilon,t}(x_1) = k^{(1)}_{\varepsilon,t}(x_1)$ and

$$u^{(2)}_{\varepsilon,t}(x_1,x_2) = k^{(2)}_{\varepsilon,t}(x_1,x_2) - k^{(1)}_{\varepsilon,t}(x_1)k^{(1)}_{\varepsilon,t}(x_2)$$

be the corresponding first- and second-order cumulants. Consider the space homogeneous case. Then, by e.g. [5],

$$k^{(1)}_{\varepsilon,t} = q_t + \varepsilon^d p_t + o(\varepsilon^d),$$

$$u^{(2)}_{\varepsilon,t}(x) = \varepsilon^d g_t(\varepsilon x) + o(\varepsilon^d).$$

Function q_t, cf. (1.22), satisfies the mean-field equation

$$\frac{d}{dt} q_t = A^+ q_t - m q_t - A^- q_t^2, \tag{3.3}$$

which can be solved explicitly:

$$q_t = \frac{q_* q_0}{q_0 + (q_* - q_0) e^{-(A^+ - m)t}}. \tag{3.4}$$

If we assume, additionally, that

$$\int_{\mathbb{R}^d} |g_0(x)| \, dx + \sup_{x \in \mathbb{R}^d} |g_0(x)| + \int_{\mathbb{R}^d} |\widetilde{g}_0(\xi)| \, dx < \infty, \tag{3.5}$$

we obtain, see [3], an equation for the Fourier transform of $g_t(x)$, namely

$$\frac{d}{dt} \widetilde{g}_t(\xi) = 2\big(\widetilde{J}_t(\xi) - A^- q_t - m\big)\widetilde{g}_t(\xi) + 2q_t \widetilde{J}_t(\xi), \tag{3.6}$$

where $J_t(x) := a^+(x) - q_t a^-(x)$, $x \in \mathbb{R}^d$, so that

$$\widetilde{J}_t(\xi) = \widetilde{a}^+(\xi) - q_t \widetilde{a}^-(\xi), \qquad \xi \in \mathbb{R}^d. \tag{3.7}$$

Equation (3.3) has two stationary solutions $q_t \equiv 0$ and $q_t \equiv q_*$, where

$$q_* := \frac{A^+ - m}{A^-}. \tag{3.8}$$

We will always assume that

$$A^+ > m, \tag{$\mathbf{SL_1}$}$$

i.e. that $q_* > 0$; otherwise, by (3.4), $\lim\limits_{t \to \infty} q_t = 0$, i.e. the population would extinct.

Under ($\mathbf{SL_1}$), we have

$$\lim_{t \to \infty} q_t = q_*. \tag{3.9}$$

Note also that

$$0 \le q_0 \le q_* \tag{3.10}$$

implies

$$0 \le q_t \le q_*, \qquad t \ge 0. \tag{3.11}$$

By (3.1), $\widetilde{J}_t$ is integrable; as a result, (3.5) holds with $\widetilde{g}_0$ replaced by $\widetilde{g}_t$. In particular, cf. (1.29),

$$g_t(x) = \int_{\mathbb{R}^d} \widetilde{g}_t(\xi) e^{2i\pi x \cdot \xi} \, d\xi, \qquad x \in \mathbb{R}^d.$$

According to (2.36), we define also

$$h_t(\xi) = \widetilde{g}_t(\xi) + q_t, \qquad \xi \in \mathbb{R}^d. \tag{3.12}$$

By (3.6) and (3.3),

$$\begin{aligned}\frac{d}{dt} h_t(\xi) &= 2\big(\widetilde{J}_t(\xi) - A^- q_t - m\big) h_t(\xi) + A^+ q_t + m q_t + A^- q_t^2 \\ &= (\mathcal{A}[q_t] h_t)(\xi) + \mathcal{B}[q_t](\xi),\end{aligned}$$

cf. (2.37), where $\mathcal{A}[q_t]$ is just a multiplication operator given by, cf. (3.7),

$$(\mathcal{A}[q_t]f)(\xi) = 2\Big(\widetilde{a}^+(\xi) - m - q_t\big(\widetilde{a}^-(\xi) + A^-\big)\Big) f(\xi) \tag{3.13}$$

for a function $f : \mathbb{R}^d \to \mathbb{R}$.

We will consider below the stationary regime, when $t \to \infty$. We define, for a.a. $x \in \mathbb{R}^d$, $J_*(x) := \lim_{t\to\infty} J_t(x)$. Then, by (3.9),

$$J_*(x) = a^+(x) - q_* a^-(x), \qquad \widetilde{J}_*(\xi) = \widetilde{a}^+(\xi) - q_* \widetilde{a}^-(\xi). \tag{3.14}$$

Note that

$$\int_{\mathbb{R}^d} J_*(x)\, dx = m. \tag{3.15}$$

We will assume, additionally to $(\mathbf{SL_1})$, that there exists $\alpha > 0$, such that

$$A^+ - \widetilde{J}_*(\xi) \geq \alpha, \qquad \xi \in \mathbb{R}^d. \tag{$\mathbf{SL_2}$}$$

Since, for an integrable function $f \geq 0$,

$$|\widetilde{f}(\xi)| \leq \int_{\mathbb{R}^d} |f(x)|\, dx = \int_{\mathbb{R}^d} f(x)\, dx, \tag{3.16}$$

one has, by (3.15) and $(\mathbf{SL_1})$, the following sufficient condition for $(\mathbf{SL_2})$:

$$J_*(x) = a^+(x) - q_* a^-(x) \geq 0, \qquad x \in \mathbb{R}^d. \tag{3.17}$$

Indeed, then $A^+ - \widetilde{J}_*(\xi) \geq A^+ - m =: \alpha > 0$, because of $(\mathbf{SL_1})$.

It was shown in [3], that if $(\mathbf{SL_1})$, $(\mathbf{SL_2})$, (3.10) and (3.5) hold, then there exists

$$\widetilde{g}_*(\xi) := \lim_{t\to\infty} \widetilde{g}_t(\xi) = \frac{q_* \widetilde{J}_*(\xi)}{A^+ - \widetilde{J}_*(\xi)}, \qquad \xi \in \mathbb{R}^d. \tag{3.18}$$

Surely $\widetilde{g}_*(\xi)$ is just the stationary solution to (3.6), i.e. it satisfies (3.6) with the left hand side replaced by 0. It was also shown in [3] that the inverse Fourier transform $g_*(x)$ of $g_*(\xi)$ is just the pointwise (and even uniform) limit of $g_t(x)$ as $t \to \infty$.

Example 3.1. The condition (3.17) holds, in particular, for

$$a^\pm(x) = A^\pm c(x) \geq 0 \qquad \text{with} \qquad \int_{\mathbb{R}^d} c(x)\, dx = 1, \tag{3.19}$$

as then $J_*(x) = mc(x) \geq 0$.

3.2 Spatiotemporal characteristics

We consider the auxiliary dynamics of $\overline{\gamma}_t = (\gamma_t^O, \gamma_t^+, \gamma_t^-)$ described by the generator

$$\overline{L} = \overline{L}_1 + \overline{L}_2 + \overline{L}_3,$$

where $\overline{L}_1, \overline{L}_2, \overline{L}_3$ are defined according to the rules postulated in Subsection 2.2.

Namely, for $\overline{\gamma} = (\gamma^O, \gamma^+, \gamma^-)$, we have

$$(\overline{L}_1 F)(\overline{\gamma}) = \sum_{x\in\gamma^O\cup\gamma^+} \int_{\mathbb{R}^d} a^+(x-y)\Bigl(F\bigl(\gamma^O, \gamma^+ \cup \{y\}, \gamma^-\bigr) - F\bigl(\overline{\gamma}\bigr)\Bigr)dy,$$

i.e. both O-reactants and $+$-reactants may produce $+$-products. Next, in the counterparts of L_2 and L_3, O-reactants become $-$-products, whereas $+$-reactants just disappear. Therefore,

$$\begin{aligned}(\overline{L}_2 F)(\overline{\gamma}) = m \sum_{x\in\gamma^O} \Bigl(F\bigl(\gamma^O \setminus \{x\}, \gamma^+, \gamma^- \cup \{x\}\bigr) - F\bigl(\overline{\gamma}\bigr)\Bigr) \\ + m \sum_{x\in\gamma^+} \Bigl(F\bigl(\gamma^O, \gamma^+ \setminus \{x\}, \gamma^-\bigr) - F\bigl(\overline{\gamma}\bigr)\Bigr),\end{aligned}$$

and since there are both O- and $+$-catalysts, we have

$$\begin{aligned}(\overline{L}_3 F)(\overline{\gamma}) = \sum_{x\in\gamma^O} \sum_{y\in(\gamma^O\setminus\{x\})\cup\gamma^+} a^-(x-y)\Bigl(F\bigl(\gamma^O \setminus \{x\}, \gamma^+, \gamma^- \cup \{x\}\bigr) - F\bigl(\overline{\gamma}\bigr)\Bigr) \\ + \sum_{x\in\gamma^+} \sum_{y\in\gamma^O\cup(\gamma^+\setminus\{x\})} a^-(x-y)\Bigl(F\bigl(\gamma^O, \gamma^+ \setminus \{x\}, \gamma^-\bigr) - F\bigl(\overline{\gamma}\bigr)\Bigr).\end{aligned}$$

Following the general scheme, we consider $\overline{L}_\varepsilon$ with $a^\pm(x-y)$ above replaced by $\varepsilon^d a^\pm(\varepsilon x - \varepsilon y)$; next, we consider the dynamics of $\overline{\gamma}_{\varepsilon,t}$ defined by (2.18) with $\overline{L}$ replaced by $\overline{L}_\varepsilon$. Let $q^A_{\Delta t}$, $g^{A,B}_{\Delta t}$, $A, B \in \{O, +, -\}$ be the corresponding functions from the beyond mean-field expansion.

Consider, cf. (2.32),

$$\widetilde{g}_{t,\Delta t}(\xi) := \widetilde{g}^{O,O}_{\Delta t}(\xi) + \widetilde{g}^{O,+}_{\Delta t}(\xi) + \widetilde{g}^{-,O}_{\Delta t}(\xi) + \widetilde{g}^{-,+}_{\Delta t}(\xi) \tag{3.20}$$

and also, cf. (2.38),

$$h_{t,\Delta t}(\xi) := \widetilde{g}_{t,\Delta t}(\xi) + q^O_{\Delta t}. \tag{3.21}$$

Then, it can be shown that (see Subsection 3.3 below for details) that

$$\frac{d}{d\Delta t} h_{t,\Delta t}(\xi) = \Bigl(\widetilde{a}^+(\xi) - m - \bigl(q^O_t + q^+_t\bigr)\bigl(\widetilde{a}^-(\xi) + A^-\bigr)\Bigr) h_{t,\Delta t}(\xi). \tag{3.22}$$

Since, by (2.33),

$$q_{t+\Delta t} = q^O_t + q^+_t, \tag{3.23}$$

we get from (3.22) and (3.13), that

$$\frac{d}{d\Delta t}h_{t,\Delta t}(\xi) = \Big(\widetilde{a}^{+}(\xi) - m - q_{t+\Delta t}\big(\widetilde{a}^{-}(\xi) + A^{-}\big)\Big)h_{t,\Delta t}(\xi) \qquad (3.24)$$
$$= \frac{1}{2}(\mathcal{A}[q_{t+\Delta t}]h_{t,\Delta t})(\xi),$$

where $\mathcal{A}$ is defined by (3.13). Therefore, the conjecture is satisfied.

One can now solve, for each $\xi \in \mathbb{R}^d$, a linear ordinary differential equation (3.24) with the initial condition

$$\frac{d}{d\Delta t}h_{t,\Delta t}(\xi)\Big|_{\Delta t=0} = h_t(\xi),$$

where $h_t(\xi)$ is given by (3.12). Then, one can get $\widetilde{g}_{t,\Delta t}(\xi)$ from (3.21); to this end, one needs $q^O_{\Delta t}$. The latter function satisfies the following differential equation (again, see Subsection 3.3 for details):

$$\frac{d}{d\Delta t}q^O_{\Delta t} = -mq^O_{\Delta t} - A^{-}(q^O_{\Delta t})^2 - A^{-}q^O_{\Delta t}q^{+}_{\Delta t} \qquad (3.25)$$
$$= -(m + A^{-}q_{t+\Delta t})q^O_{\Delta t},$$

where we used (3.23). As a result, we will get the following statement.

Theorem 3.2. *Let* (3.1), $(\mathbf{SL_1})$, (3.5) *hold. Then, for any* $t, \Delta t \geq 0$, $\xi \in \mathbb{R}^d$,

$$\widetilde{g}_{t,\Delta t}(\xi) = e^{(\widetilde{J}_*(\xi)-A^{+})\Delta t}\Big(\frac{q_{t+\Delta t}}{q_t}\Big)^{\frac{\widetilde{a}^{-}(\xi)+A^{-}}{A^{-}}}\big(\widetilde{g}_t(\xi) + q_t\big) - e^{-A^{+}\Delta t}q_{t+\Delta t} \qquad (3.26)$$

where q_t *and* $q_{t+\Delta t}$ *can be obtained from* (3.4). *If, additionally,* (3.10) *and* $(\mathbf{SL_2})$ *hold, then* $\widetilde{g}_{t,\Delta t}(\xi)$ *is an integrable function, and one can apply the inverse Fourier transform to it, to get* $g_{t,\Delta t}(x)$ *for a.a.* x. *Then, for all* $t \geq 0$ *and a.a.* $x \in \mathbb{R}^d$,

$$\lim_{\Delta t\to\infty} g_{t,\Delta t}(x) = 0. \qquad (3.27)$$

Moreover, for all $\Delta t \geq 0$, $\xi \in \mathbb{R}^d$, *there exists*

$$\widetilde{g}_{\infty,\Delta t}(\xi) := \lim_{t\to\infty}\widetilde{g}_{t,\Delta t}(\xi) = \frac{A^{+}q_*}{A^{+} - \widetilde{J}_*(\xi)}e^{(\widetilde{J}_*(\xi)-A^{+})\Delta t} - q_*e^{-A^{+}\Delta t}, \qquad (3.28)$$

and $\widetilde{g}_{\infty,\Delta t}(\xi)$ *is an integrable function. Let* $g_{\infty,\Delta t}(x)$ *be its inverse Fourier transform. If, additionally,* $g_0(x)$ *and* $\widetilde{g}_0(\xi)$ *are both integrable, then, for all* $\Delta t \geq 0$, *the following limit holds uniformly in a.a.* $x \in \mathbb{R}^d$:

$$g_{\infty,\Delta t}(x) = \lim_{t\to\infty} g_{t,\Delta t}(x). \qquad (3.29)$$

3.3 Derivation of equations

In this Subsection, we are going to derive equations (3.22) and (3.25).

We will partially use the Model Constructor toolbox presented in [1]. Firstly, we express $\overline{L}_1, \overline{L}_2, \overline{L}_3$ given above as sums of *model components* in the terminology of [1, Supplementary Note 2]:

$$\overline{L}_1 = L_{11} + L_{12}, \qquad \overline{L}_2 = L_{21} + L_{22}, \qquad \overline{L}_3 = L_{31} + L_{22} + L_{33} + L_{34}.$$

Here L_{11} represents the `Birth` component:

$$(L_{11}F)(\overline{\gamma}) = \sum_{x\in\gamma^+} \int_{\mathbb{R}^d} a^+(x-y)\Big(F\big(\gamma^O, \gamma^+ \cup \{y\}, \gamma^-\big) - F\big(\overline{\gamma}\big)\Big)dy;$$

L_{12} represents the `BirthToAnotherType` component:

$$(L_{12}F)(\overline{\gamma}) = \sum_{x\in\gamma^O} \int_{\mathbb{R}^d} a^+(x-y)\Big(F\big(\gamma^O, \gamma^+ \cup \{y\}, \gamma^-\big) - F\big(\overline{\gamma}\big)\Big)dy;$$

L_{21} represents the `DensityIndependentDeath` component:

$$(L_{21}F)(\overline{\gamma}) = m \sum_{x\in\gamma^+} \Big(F\big(\gamma^O, \gamma^+ \setminus \{x\}, \gamma^-\big) - F\big(\overline{\gamma}\big)\Big);$$

L_{22} represents the `ChangeInType` component:

$$(L_{22}F)(\overline{\gamma}) = m \sum_{x\in\gamma^O} \Big(F\big(\gamma^O \setminus \{x\}, \gamma^+, \gamma^- \cup \{x\}\big) - F\big(\overline{\gamma}\big)\Big);$$

L_{31} represents `DeathByCompetition`:

$$(L_{31}F)(\overline{\gamma}) = \sum_{x\in\gamma^+} \sum_{y\in\gamma^+\setminus\{x\}} a^-(x-y)\Big(F\big(\gamma^O, \gamma^+ \setminus \{x\}, \gamma^-\big) - F\big(\overline{\gamma}\big)\Big);$$

L_{32} represents `DeathByExternalFactor`:

$$(L_{32}F)(\overline{\gamma}) = \sum_{x\in\gamma^+} \sum_{y\in\gamma^O} a^-(x-y)\Big(F\big(\gamma^O, \gamma^+ \setminus \{x\}, \gamma^-\big) - F\big(\overline{\gamma}\big)\Big);$$

L_{33} and L_{34} require a new component called `ChangeInTypeByFacilitation` (which is defined below):

$$(L_{33}F)(\overline{\gamma}) = \sum_{x\in\gamma^O} \sum_{y\in\gamma^O\setminus\{x\}} a^-(x-y)\Big(F\big(\gamma^O \setminus \{x\}, \gamma^+, \gamma^- \cup \{x\}\big) - F\big(\overline{\gamma}\big)\Big)$$

and

$$(L_{34}F)(\overline{\gamma}) = \sum_{x\in\gamma^O} \sum_{y\in\gamma^+} a^-(x-y)\Big(F\big(\gamma^O \setminus \{x\}, \gamma^+, \gamma^- \cup \{x\}\big) - F\big(\overline{\gamma}\big)\Big).$$

The Model Constructor is written on Wolfram Language and requires Wolfram Mathematica® v10 or later. The Model Constructor packages are available at [2] and should be installed before running the following code.

Firstly, we load libraries and set-up internal variables:

```
In[1]:= Get["SSPPlibraryOfProcesses`"]   (*Load libraries*)
        Get["SSPPanalyticalExpressions`"]
        qpgVariables={q,p,g};  (*Set-up variables*)
        kVariable=ξ;
```

Next, we define the ChangeInTypeByFacilitation model component needed for L_{33} and L_{34} above. It describes the event when an agent at a position x_1 changes own type from s_2 to s_1. The event happened because of interaction of the agent with each of other agents of a type s_3 placed at a position x_2. The interaction is defined through a kernel $a(x_1 - x_2)$. In particular, s_3 may be equal to s_1 as it is needed for L_{33}.

```
In[5]:= ChangeInTypeByFacilitation[s1_,s2_,s3_,a_,Af_,Coeffic_]:=
            Module[{Products={{s1,x1}},
               Reactants={{s2,x1}}, Catalysts={{s3,x2}},
               listAll,function,Interactions,name},
               listAll={Products,Reactants,Catalysts};
               function[x1_,x2_]:=a[x1-x2];
               Interactions={{a,Af,x1,x2}};
               name="ChangeInTypeByFacilitation";
               {listAll,function,Interactions,name,Coeffic}];
```

Note that −-agents do not have own dynamics, and appear only when O-agents are transformed to them. We, however, require the characteristics of −-agents, hence, we introduce a trivial model component where agents die with the rate 0, i.e. effectively nothing happens:

```
In[6]:= (*For agents without own dynamics*)
        Relax[type_]:=DensityIndependentDeath[type,1,0];
```

We define now the AuxiliaryProcess which includes all model components corresponding to operators L_{ij} above. Here the agent types $1, 2, 3$ correspond to $O, +, -$, respectively.

```
In[7]:= (*Define auxiliary process*)
        AuxiliaryProcess={
               Birth[2,a⁺,ã⁺,1],(*L11*)
               BirthToAnotherType[2,1,a⁺,ã⁺,1], (*L12*)
               DensityIndependentDeath[2,m,1], (*L21*)
               ChangeInType[3,1,m,1], (*L22*)
               DeathByCompetition[2,a⁻,ã⁻,1], (*L31*)
               DeathByExternalFactor[2,1,a⁻,ã⁻,1], (*L32*)
               ChangeInTypeByFacilitation[3,1,1,a⁻,ã⁻,1], (*L33*)
               ChangeInTypeByFacilitation[3,1,2,a⁻,ã⁻,1], (*L34*)
               Relax[3] (*No dynamics of '-' agents*)
               };
```

We sum up now the right hand sides of the differential equations for the needed $\widetilde{g}_{\Delta t}^{A,B}(\xi)$, $A, B \in \{O, +, -\}$, and $q_{\Delta t}^{O}$, cf. (3.20), (3.21). In the notations of the Model Constructor:

$$q_{\Delta t}^{O} = q[1], \qquad \widetilde{g}_{\Delta t}^{O,O}(\xi) = g[1,1,\xi], \qquad \widetilde{g}_{\Delta t}^{O,+}(\xi) = g[1,2,\xi],$$
$$\widetilde{g}_{\Delta t}^{-,O}(\xi) = g[3,1,\xi] = g[1,3,\xi], \qquad \widetilde{g}_{\Delta t}^{-,+}(\xi) = g[3,2,\xi] = g[3,3,\xi].$$

We use the following code:

In[18]:=
```
(*Get the whole equation for h*)
hEqn=HGfALL[qpgVariables,AuxiliaryProcess,1,1,ξ]
        +HGfALL[qpgVariables,AuxiliaryProcess,1,2,ξ]
        +HGfALL[qpgVariables,AuxiliaryProcess,1,3,ξ]
        +HGfALL[qpgVariables,AuxiliaryProcess,2,3,ξ]
        +HQfALL[qpgVariables,AuxiliaryProcess,1];
```

We are going to verify now (3.22); to this end, we define the expression for $h := h_{t,\Delta t}(\xi)$, cf. (3.20), (3.21):

In[19]:=
```
(*Define h*)
h=g[1,1,ξ]+g[1,2,ξ]+g[1,3,ξ]+g[2,3,ξ]+q[1];
```

Finally, we equate Ch with the obtained sum of the right hand sides of the equation, and find C, that is nothing but $\frac{1}{2}\mathcal{A}$ in (3.24):

In[20]:=
```
(*Find the coefficient*)
Reduce[C h==hEqn,C]
```

Out[20]=
```
C==-m-q[1] ã⁻[0]-q[2] ã⁻[0]-q[1] ã⁻[ξ]-q[2] ã⁻[ξ]+ã⁺[ξ]
|| g[1,1,ξ]==-g[1,2,ξ]-g[1,3,ξ]-g[2,3,ξ]-q[1]
```

Here $q[2] = q_{\Delta t}^{+}$ and also, by the very definition (1.29) of the Fourier transform:

$$\widetilde{a}^{-}(0) = \int_{\mathbb{R}^d} a^{-}(x)\, dx = A^{-}.$$

Therefore, the found expression for C coincides with the factor before $h_{t,\Delta t}(\xi)$ in the right hand side of (3.22). The second found alternative just means that $h = 0$, i.e. that $h_{t,\Delta t}(\xi) \equiv 0$ also solves (3.22), that is trivial. Hence, (3.22) is fulfilled.

To get (3.25), we just consider the right hand side of the equation for $q[1]$:

In[21]:=
```
HQfALL[qpgVariables,AuxiliaryProcess,1]
```

Out[21]=
```
-m q[1]-q[1]² ã⁻[0]-q[1] q[2] ã⁻[0]
```

3.4 Numerics for the stationary regime on plane

We consider the 2-dimensional case: $d = 2$, and radially symmetric kernels with equal Gaussian shapes:

$$a^{\pm}(x) = A^{\pm}\beta(|x|), \quad x \in \mathbb{R}^2; \qquad \beta(r) = \frac{1}{2\pi} e^{-\frac{r^2}{2}}, \quad r \geq 0. \tag{3.30}$$

Note that, indeed, $\int_{\mathbb{R}^2} \beta(|x|)\, dx = 1$, and also, see Example 3.1, the assumption (3.17) holds, hence ($\mathbf{SL_2}$) holds. We will need the following lemma.

Lemma 3.3. *Denote $c(x) = \beta(|x|)$, $x \in \mathbb{R}^d$, where β is given by (3.30). Then*

$$\widetilde{c}(\xi) = e^{-2\pi^2|\xi|^2}, \qquad \xi \in \mathbb{R}^2. \tag{3.31}$$

Moreover, if f is a function such that the function $\widetilde{g}(\xi) := f(\widetilde{c}(\xi))$, $\xi \in \mathbb{R}^2$ is integrable, then the inverse Fourier transform $g(x)$of $\widetilde{g}(\xi)$ can be found by the formula

$$g(x) = \frac{1}{2\pi}\int_0^\infty f\bigl(e^{-\frac{s^2}{2}}\bigr)\, s \mathtt{J}_0\bigl(s|x|\bigr)\, ds, \qquad x \in \mathbb{R}^2, \tag{3.32}$$

where $\mathtt{J}_0$ is the Bessel function of the first kind.

The simulations described in the main text were done with

$$A^+ = 2, \qquad A^- = 1, \qquad m = 1.$$

Then, by (3.8), $q_* = 1$, and, by (3.28),

$$\widetilde{g}_{\infty,\Delta t}(\xi) := \lim_{t\to\infty} \widetilde{g}_{t,\Delta t}(\xi) = \widetilde{g}_{\infty,\Delta t}(\xi) = \frac{2}{2 - e^{-2\pi^2|\xi|^2}} e^{(e^{-2\pi^2|\xi|^2}-2)\Delta t} - e^{-2\Delta t}.$$

Therefore, by (3.32),

$$g_{\infty,\Delta t}(x) = \frac{1}{2\pi}\int_0^\infty \left(\frac{2}{2 - e^{-\frac{s^2}{2}}} e^{(e^{-\frac{s^2}{2}}-2)\Delta t} - e^{-2\Delta t}\right) s J_0\bigl(s|x|\bigr)\, ds =: g(\Delta t, |x|).$$

The latter integral can be calculated numerically. We use the following Wolfram Mathematica code (where `dt` $= \Delta t$ and `r` $= |x|$):

```
In[22]:=  g[dt_?NumericQ,r_?NumericQ]:=g[dt,r]
              =1/(2π) NIntegrate[(2/(2-Exp[-s^2/2]) Exp[(Exp[-s^2/2]-2)dt]
                   -Exp[-2dt]) s BesselJ[0,s r],{s,0,∞},
              Method →{LocalAdaptive,SymbolicProcessing→0},
              PrecisionGoal→7];
```

The simulations were done with $\varepsilon = \frac{1}{2}$. The covariance between numbers of agents in two areas satisfies (2.25); note that we are actually interested in the covariance between 'small' areas (a local characteristic), so we may assume that

they are disjoint, hence, the second summand in the right hand side of (2.25) is redundant. Next, the value of $u_{\varepsilon,t,\Delta t}(x)$ can be approximated by the formula (2.31), with $d = 2$ and $\varepsilon = \frac{1}{2}$, in our case. Therefore, we are interested in

$$\lim_{t\to\infty} u_{\varepsilon,t,\Delta t}(x) \approx \varepsilon^2 \lim_{t\to\infty} g_{t,\Delta t}(\varepsilon x) = \frac{1}{4} g_{\infty,\Delta t}\left(\frac{x}{2}\right) = \frac{1}{4} g\left(\Delta t, \frac{|x|}{2}\right).$$

We plot now graphs for $\frac{1}{4} g\left(\Delta t, \frac{r}{2}\right)$ with $\Delta t \in \{0, 1, 2\}$, $r \in [0, 10]$:

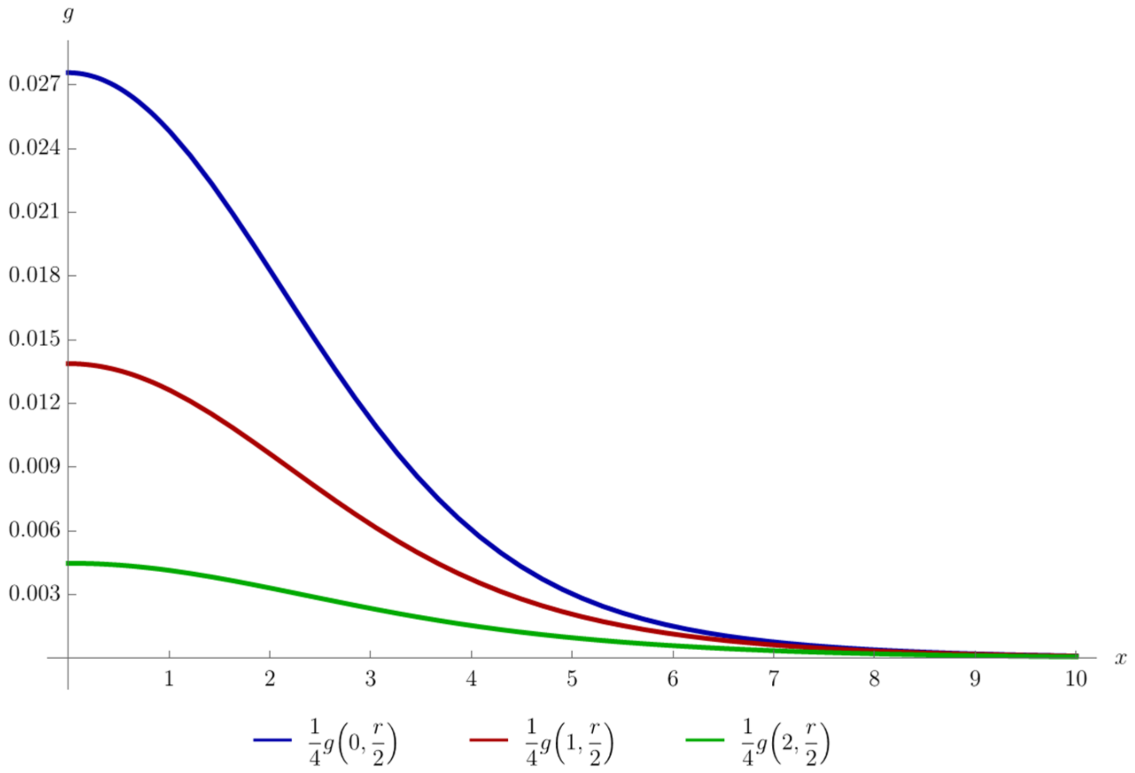

Figure 1: Graphs for $A^+ = 2, A^- = m = 1$

4 Case study 2: Host-parasite model

4.1 Spatial characteristics

We consider now agents of two types, called *hosts* and *parasites*:

$$N = 2, \qquad \mathtt{I}_2 = \{H, P\}.$$

Let L be given through some of three operators, cf. 1.4:

$$L = L_1 + L_2 + L_3 + L_4.$$

Here L_1 describes an independent birth process of hosts: any H-catalyst sends an off-spring which is an H-product, according to a dispersion kernel $a^+ \geq 0$:

$$(L_1F)(\gamma^H, \gamma^P) = \sum_{x\in\gamma^H} \int_{\mathbb{R}^d} a^+(x-y)\bigl(F(\gamma^H \cup y, \gamma^P) - F(\gamma^H, \gamma^P)\bigr)\,dy;$$

next, hosts may die because of the competition with other hosts (for resources), according to a competition kernel $a^- \geq 0$:

$$(L_2F)(\gamma^H, \gamma^P) = \sum_{x\in\gamma^H} \sum_{y\in\gamma^H\setminus\{x\}} a^-(x-y)\bigl(F(\gamma^H \setminus \{x\}, \gamma^P) - F(\gamma^H, \gamma^P)\bigr);$$

next, parasites may die with a constant mortality rate $m > 0$:

$$(L_3F)(\gamma^H, \gamma^P) = m \sum_{x\in\gamma^P} \bigl(F(\gamma^H, \gamma^P \setminus x) - F(\gamma^H, \gamma^P)\bigr);$$

finally, any host may be transformed to a parasite (keeping the position) because of interaction with the existing parasites, according to a kernel $b \geq 0$:

$$(L_4F)(\gamma^H, \gamma^P) = \sum_{x\in\gamma^H} \sum_{y\in\gamma^P} b(x-y)\bigl(F(\gamma^H \setminus x, \gamma^P \cup x) - F(\gamma^H, \gamma^P).$$

We will assume that (3.1) holds for both $a^\pm$ and for b, we define $A^\pm$ through (3.2), and set, similarly,

$$B = \int_{\mathbb{R}^d} b(x)\,dx.$$

We consider L_ε by replacing $a^\pm(x-y)$ and $b(x-y)$ by $\varepsilon^d a^\pm(\varepsilon x - \varepsilon y)$ and $\varepsilon^d b(\varepsilon x - \varepsilon y)$, respectively. We consider the space homogeneous case. Then, by the general scheme described in Section 1,

$$\begin{gathered}
k^H_{\varepsilon,t} = q^H_t + \varepsilon^d p^H_t + o(\varepsilon^d), \qquad k^P_{\varepsilon,t} = q^P_t + \varepsilon^d p^P_t + o(\varepsilon^d), \\
u^{HH}_{\varepsilon,t}(x) = \varepsilon^d g^{HH}_t(\varepsilon x) + o(\varepsilon^d), \qquad u^{PP}_{\varepsilon,t}(x) = \varepsilon^d g^{PP}_t(\varepsilon x) + o(\varepsilon^d), \\
u^{HP}_{\varepsilon,t}(x) = u^{PH}_{\varepsilon,t}(x) = \varepsilon^d g^{HP}_t(\varepsilon x) + o(\varepsilon^d).
\end{gathered} \tag{4.1}$$

We define also, cf. (2.36),

$$\begin{gathered} h_t^{HH}(\xi) := \widetilde{g}_t^{HH}(\xi) + q_t^H, \qquad h_t^{PP}(\xi) := \widetilde{g}_t^{PP}(\xi) + q_t^P, \\ h_t^{HP}(\xi) = h_t^{PH}(\xi) := \widetilde{g}_t^{HP}(\xi). \end{gathered} \tag{4.2}$$

Differential equations for q_t^A and $\widetilde{h}_t^{AB}(\xi) = \widetilde{g}_t^{BA}(\xi)$, $A, B \in \{H, P\}$ are derived in Subsection 4.3 below. We will show that

$$\begin{aligned} \frac{d}{dt} q_t^H &= q_t^H \bigl(A^+ - A^- q_t^H - B q_t^P\bigr), \\ \frac{d}{dt} q_t^P &= q_t^P \bigl(B q_t^H - m\bigr). \end{aligned} \tag{4.3}$$

Next, we define

$$\begin{gathered} \mathsf{a}_t(\xi) := \widetilde{a}^+(\xi) - \bigl(A^- + \widetilde{a}^-(\xi)\bigr) q_t^H - B q_t^P, \\ \mathsf{b}_t(\xi) := -q_t^H \widetilde{b}(\xi), \qquad \mathsf{c}_t := B q_t^P, \qquad \mathsf{d}_t(\xi) := q_t^H \widetilde{b}(\xi) - m, \end{gathered} \tag{4.4}$$

and consider the matrix

$$\mathcal{A}_t(\xi) := \begin{pmatrix} \mathsf{a}_t(\xi) & \mathsf{b}_t(\xi) & 0 & 0 \\ \mathsf{c}_t & \mathsf{d}_t(\xi) & 0 & 0 \\ 0 & 0 & \mathsf{a}_t(\xi) & \mathsf{b}_t(\xi) \\ 0 & 0 & \mathsf{c}_t & \mathsf{d}_t(\xi) \end{pmatrix} \tag{4.5}$$

The second and third rows will correspond to $h_t^{HP}(\xi)$ and $h_t^{PH}(\xi)$ which are equal. Hence, we consider also the matrix with swapped second and third rows

$$\mathcal{A}'_t(\xi) := \begin{pmatrix} \mathsf{a}_t(\xi) & \mathsf{b}_t(\xi) & 0 & 0 \\ 0 & 0 & \mathsf{a}_t(\xi) & \mathsf{b}_t(\xi) \\ \mathsf{c}_t & \mathsf{d}_t(\xi) & 0 & 0 \\ 0 & 0 & \mathsf{c}_t & \mathsf{d}_t(\xi) \end{pmatrix}. \tag{4.6}$$

Finally, we define the vector-function

$$\mathcal{B}_t = \Bigl(A^+ q_t^H + A^- \left(q_t^H\right)^2, 0, 0, m q_t^P\Bigr)^T + B q_t^H q_t^P \Bigl(1, -1, -1, 1\Bigr)^T; \tag{4.7}$$

henceforth, the superscript T denotes the transpose vector. Note that

$$\mathcal{A}_t = \mathcal{A}[q_t^H, q_t^P], \qquad \mathcal{A}'_t = \mathcal{A}'[q_t^H, q_t^P], \qquad \mathcal{B}_t = \mathcal{B}[q_t^H, q_t^P]. \tag{4.8}$$

Then, we will show in Subsection 4.3 that, for the vector

$$\overline{h}_t(\xi) := \Bigl(h_t^{HH}(\xi), h_t^{HP}(\xi), h_t^{HP}(\xi), h_t^{PP}(\xi)\Bigr)^T, \tag{4.9}$$

we have, cf. (2.37),

$$\frac{d}{dt} \overline{h}_t(\xi) = \Bigl(\mathcal{A}_t(\xi) + \mathcal{A}'_t(\xi)\Bigr) \overline{h}_t(\xi) + \mathcal{B}_t(\xi). \tag{4.10}$$

We consider now the stationary regime when $t \to \infty$. The only pair of non-zero stationary solutions of (4.3) is

$$q_*^H := \frac{m}{B}, \qquad q_*^P := \frac{BA^+ - mA^-}{B^2}. \tag{4.11}$$

Therefore, the condition

$$A^+ > \frac{m}{B} A^- \tag{$\mathbf{HP_1}$}$$

ensures that (q_*^H, q_*^P) is a the only pair of *positive* stationary solutions to (4.3).

Proposition 4.1. *Let* ($\mathbf{HP_1}$) *hold. Then, for any* $q_0^H > 0$, $q_0^P > 0$,

$$\lim_{t\to\infty} q_t^H = q_*^H, \qquad \lim_{t\to\infty} q_t^P = q_*^P. \tag{4.12}$$

More precisely, if

$$\frac{m}{B}A^- < A^+ \le \frac{m}{B}A^- + \frac{m}{4B^2}(A^-)^2,$$

then both convergences, q_t^H *to* q_*^H *and* q_t^P *to* q_*^P, *are monotone, whereas if*

$$A^+ > \frac{m}{B}A^- + \frac{m}{4B^2}(A^-)^2, \tag{4.13}$$

then q_t^H *and* q_t^P *oscillate around* q_*^H *and* q_*^P, *respectively, with a decreasing amplitude (damping oscillation).*

We define also the following analogue of (3.14): for $x, \xi \in \mathbb{R}^d$, we set

$$J_*^H(x) := a^+(x) - q_*^H a^-(x), \qquad \widetilde{J}_*^H(x) = \widetilde{a}^+(\xi) - q_*^H \widetilde{a}^-(\xi). \tag{4.14}$$

We will assume that there exist $\alpha > 0$, such that

$$A^+ - \widetilde{J}_*^H(\xi) \ge \alpha, \qquad \xi \in \mathbb{R}^d, \tag{$\mathbf{HP_2}$}$$

$$\bigl(A^+ - \widetilde{J}_*^H(\xi)\bigr)\bigl(B - \widetilde{b}(\xi)\bigr) + Bq_*^P \widetilde{b}(\xi) \ge \alpha, \qquad \xi \in \mathbb{R}^d. \tag{$\mathbf{HP_3}$}$$

The following proposition provides simple sufficient conditions for ($\mathbf{HP_1}$)–($\mathbf{HP_3}$).

Proposition 4.2. *Suppose that*

$$J_*^H(x) = a^+(x) - q_*^H a^-(x) \ge 0, \qquad x \in \mathbb{R}^d; \tag{4.15}$$

$$\widetilde{b}(\xi) \ge 0, \qquad \xi \in \mathbb{R}^d. \tag{4.16}$$

Then ($\mathbf{HP_1}$)–($\mathbf{HP_3}$) *hold.*

Example 4.3. An example when (4.15) holds is the case (3.19) of an equal shape c of kernels $a^+ = A^+c$ and $a^- = A^-c$, provided that ($\mathbf{HP_1}$) holds. Indeed, then

$$J_*^H(x) = \bigl(A^+ - \frac{m}{B}A^-\bigr)c(x) \ge 0.$$

In the Appendix below, we consider also how the condition (4.16) can be relaxed to still get ($\mathbf{HP_3}$).

We denote the limits as $t \to \infty$ of the functions $\mathtt{a}_t(\xi)$, $\mathtt{b}_t(\xi)$, $\mathtt{c}_t$, $\mathtt{d}_t(\xi)$, defined in (4.4), by $\mathtt{a}_*(\xi)$, $\mathtt{b}_*(\xi)$, $\mathtt{c}_*$, $\mathtt{d}_*(\xi)$, respectively. Then, by (4.12), ($\mathbf{HP_2}$), (3.16),

$$\begin{aligned} \mathtt{a}_*(\xi) &= \widetilde{J}^H_*(\xi) - A^+ < 0, & \mathtt{b}_*(\xi) &= -q^H_* \widetilde{b}(\xi), \\ \mathtt{c}_* &= B q^P_*, & \mathtt{d}_*(\xi) &= q^H_* \bigl(\widetilde{b}(\xi) - B\bigr) \leq 0. \end{aligned} \tag{4.17}$$

Theorem 4.4. *Let* ($\mathbf{HP_1}$)*–*($\mathbf{HP_3}$) *hold. Suppose that* $g^{AB}_0(x), \widetilde{g}^{AB}_0(\xi)$ *are bounded and integrable for* $A, B \in \{H, P\}$*. Then* $\widetilde{g}^{AB}_t(\xi)$ *converge (uniformly in* ξ*) as* $t \to \infty$ *to integrable functions* $\widetilde{g}^{AB}_*(\xi)$ *given by*

$$\Bigl(\widetilde{g}^{HH}_*(\xi), \widetilde{g}^{HP}_*(\xi), \widetilde{g}^{PP}_*(\xi)\Bigr)^T = q^H_* \Bigl(\mathcal{C}_*(\xi)\Bigr)^{-1} \Bigl(-2\widetilde{J}^H_*(\xi), q^P_*\widetilde{b}(\xi), -2q^P_*\widetilde{b}(\xi)\Bigr)^T,$$

where $\mathcal{C}_*(\xi)$ *is the following invertible matrix:*

$$\mathcal{C}_*(\xi) = \begin{pmatrix} 2\mathtt{a}_*(\xi) & 2\mathtt{b}_*(\xi) & 0 \\ \mathtt{c}_* & \mathtt{a}_*(\xi) + \mathtt{d}_*(\xi) & \mathtt{b}_*(\xi) \\ 0 & 2\mathtt{c}_* & 2\mathtt{d}_*(\xi) \end{pmatrix}. \tag{4.18}$$

Moreover, for $A, B \in \{H, P\}$*,* $g^{AB}_t(x)$ *converges as* $t \to \infty$ *to the inverse Fourier transform* $g^{AB}_*(x)$ *of* $\widetilde{g}^{AB}_*(\xi)$ *uniformly in a.a.* $x \in \mathbb{R}^d$*.*

We consider matrices $\mathcal{A}_*(\xi)$ and $\mathcal{A}'_*(\xi)$ such that their entries are just the limits as $t \to \infty$ of those in (4.5)–(4.6), respectively. We set also

$$\mathcal{B}_* := \lim_{t\to\infty} \mathcal{B}_t = \bigl(2A^+ q^H_*, -mq^P_*, -mq^P_*, 2mq^P_*\bigr)^T. \tag{4.19}$$

We define

$$h^{HH}_*(\xi) := \widetilde{g}^{HH}_*(\xi) + q^H_*, \qquad h^{PP}_*(\xi) := \widetilde{g}^{PP}_*(\xi) + q^P_*,$$
$$h^{HP}_*(\xi) := h^{PH}_*(\xi) := \widetilde{g}^{HP}_*(\xi) = \widetilde{g}^{PH}_*(\xi),$$

and let

$$\overline{h}_*(\xi) = \Bigl(h^{HH}_*(\xi), h^{HP}_*(\xi), h^{HP}_*(\xi), h^{PP}_*(\xi)\Bigr)^T. \tag{4.20}$$

Corollary 4.5. *Let* ($\mathbf{HP_1}$)*–*($\mathbf{HP_3}$) *hold. Then* $\overline{h}_*(\xi)$ *is the unique solution to the following stationary counterpart of* (4.10)*:*

$$\Bigl(\mathcal{A}_*(\xi) + \mathcal{A}'_*(\xi)\Bigr)\overline{h}_*(\xi) + \mathcal{B}_*(\xi) = 0, \tag{4.21}$$

Moreover,

$$\Bigl(h^{HH}_*(\xi), h^{HP}_*(\xi), h^{PP}_*(\xi)\Bigr)^T = \Bigl(\mathcal{C}_*(\xi)\Bigr)^{-1} \bigl(-2A^+ q^H_*, mq^P_*, -2mq^P_*\bigr)^T. \tag{4.22}$$

4.2 Spatiotemporal characteristics

Similarly to Subsection 3.2, we consider the auxiliary dynamics of

$$\overline{\gamma}_t = (\gamma_t^O, \gamma_t^+, \gamma_t^-),$$

described by the generator

$$\overline{L} = \overline{L}_1 + \overline{L}_2 + \overline{L}_3 + \overline{L}_4,$$

where $\overline{L}_1, \overline{L}_2, \overline{L}_3, \overline{L}_4$ are defined according to Subsection 2.2. Namely, now

$$\overline{\mathrm{I}}_2 = \{HO, H+, H-, PO, P+, P-\},$$

and, for

$$\overline{\gamma} := (\overline{\gamma}^H, \overline{\gamma}^P) := (\gamma^{HO}, \gamma^{H+}, \gamma^{H-}, \gamma^{PO}, \gamma^{P+}, \gamma^{P-}),$$

we have, similarly to the corresponding operators in spatial logistic model

$$(\overline{L}_1 F)(\overline{\gamma}) = \sum_{x\in\gamma^{HO}\cup\gamma^{H+}} \int_{\mathbb{R}^d} a^+(x-y)\Big(F(\gamma^{HO}, \gamma^{H+}\cup\{y\}, \gamma^{H-}, \overline{\gamma}^P) - F(\overline{\gamma})\Big)\,dy;$$

$$\begin{aligned}(\overline{L}_2 F)(\overline{\gamma}) = &\sum_{x\in\gamma^{H+}} \sum_{y\in\gamma^{HO}\cup(\gamma^{H+}\setminus\{x\})} a^-(x-y)\Big(F(\gamma^{HO}, \gamma^{H+}\setminus\{x\}, \gamma^{H-}, \overline{\gamma}^P) - F(\overline{\gamma})\Big)\Big)\\ &+ \sum_{x\in\gamma^{HO}} \sum_{y\in(\gamma^{HO}\setminus\{x\})\cup\gamma^{H+}} a^-(x-y)\Big(F(\gamma^{HO}\setminus\{x\}, \gamma^{H+}, \gamma^{H-}\cup\{x\}, \overline{\gamma}^P) - F(\overline{\gamma})\Big);\end{aligned}$$

$$\begin{aligned}(\overline{L}_3 F)(\overline{\gamma}) = &\, m\sum_{x\in\gamma^{PO}} \Big(F(\overline{\gamma}^H, \gamma^{PO}\setminus\{x\}, \gamma^{P+}, \gamma^{P-}\cup\{x\}) - F(\overline{\gamma})\Big)\\ &+ m\sum_{x\in\gamma^{P+}} \Big(F(\overline{\gamma}^H, \gamma^{PO}, \gamma^{P+}\setminus\{x\}, \gamma^{P-}) - F(\overline{\gamma})\Big).\end{aligned}$$

The situation with $\overline{L}_4$ is more complicated: when HO-reactant of the spatial dynamics becomes $P+$-product (keeping the position), it should be also transformed to $H-$-product, according to the general scheme.

Hence, formally, one could write:

$$\begin{aligned}(\overline{L}_4' F)(\overline{\gamma}) = &\sum_{x\in\gamma^{HO}} \sum_{y\in\gamma^{PO}\cup\gamma^{P+}} b(x-y)\\ &\quad\times\Big(F(\gamma^{HO}\setminus\{x\}, \gamma^{H+}, \gamma^{H-}\cup\{x\}, \gamma^{PO}, \gamma^{P+}\cup\{x\}, \gamma^{P-}) - F(\overline{\gamma})\Big)\\ &+ \sum_{x\in\gamma^{H+}} \sum_{y\in\gamma^{PO}\cup\gamma^{P+}} b(x-y)\\ &\quad\times\Big(F(\gamma^{HO}, \gamma^{H+}\setminus\{x\}, \gamma^{H-}, \gamma^{PO}, \gamma^{P+}\cup\{x\}, \gamma^{P-}) - F(\overline{\gamma})\Big).\end{aligned}$$

However, the first summand in the latter expression does not satisfy the basic requirement that different agents should have different positions: there are agents

x^{H-} and x^{P+} simultaneously.[1] To overcome this, we consider a formal modification the host x^{HO} transforms to the parasite z^{P+} distributed in space according to the kernel given by the Dirac δ-function $\delta(x-z)$.

Therefore, we define

$$
\begin{aligned}
&(\overline{L}_4F)(\overline{\gamma}) \\
&= \sum_{x\in\gamma^{HO}} \sum_{y\in\gamma^{PO}\cup\gamma^{P+}} b(x-y) \\
&\quad\times \int_{\mathbb{R}^d} \delta(x-z)\Big(F(\gamma^{HO}\setminus\{x\},\gamma^{H+},\gamma^{H-}\cup\{x\},\gamma^{PO},\gamma^{P+}\cup\{z\},\gamma^{P-}) - F(\overline{\gamma})\Big)\,dz \\
&\quad+ \sum_{x\in\gamma^{H+}} \sum_{y\in\gamma^{PO}\cup\gamma^{P+}} b(x-y) \\
&\quad\times \Big(F(\gamma^{HO},\gamma^{H+}\setminus\{x\},\gamma^{H-},\gamma^{PO},\gamma^{P+}\cup\{x\},\gamma^{P-}) - F(\overline{\gamma})\Big).
\end{aligned}
$$

Considering the first summand of the operator $\overline{L}_4$ as such with a regular (integrable) kernel $\delta(x-z)$ one apply the technique described above and derive the corresponding differential equations for correlation functions, cumulants, and for the beyond mean-field expansion. We should then 'replace' the regular kernel by the real δ-function. This latter includes two steps. Firstly, in differential equations in terms of the Fourier transform, we replace $\widetilde{\delta}(\xi)$ by 1.

Secondly, one has to distinguish the terms which, in real coordinates (before passing to the Fourier transform), contained the δ-function, i.e.

$$\delta(x^{HO}-z^{P+}) = \delta(x-z).$$

If such term contained also the point x^{H-}, then, after the integration w.r.t. $z^{P+}=z$, the corresponding integral would disappear, and z will be replaced by x. The latter is however the position of the $H-$-agent. We will get as a result the δ-function between the positions of $H-$ and $P+$ agents. This can be written heuristically as follows: for a (regular) function f,

$$f(x^{H-})\delta(x^{HO}-z^{P+}) = f(x^{H-})\delta(x^{H-}-z^{P+}).$$

It means that the pair correlation between $H-$ and $P+$ will include now a δ-function. By (2.17) (considered in the case $\mathtt{i},\mathtt{j}=\mathtt{I}_2=\{H,P\}$), pair cumulant $u_{\Delta t}^{H-,P+} = u_{\Delta t}^{P+,P-}$ in the r.h.s. appears for $u_{t,\Delta t}^{HP}$ in the l.h.s. only.

Hence, in the space homogeneous case, $u_{t,\Delta t}^{HP}(x)$ is (the only) non-integrable pair cumulant. This is the effect of using $\overline{L}_4$ instead of $\overline{L}_4'$. Since the dynamics we consider is linear, one has

$$u_{t,\Delta t}^{HP}(x) = u_{t,\Delta t}^{HP'}(x) + u_{t,\Delta t,\mathrm{sing}}^{HP}\delta(x), \tag{4.23}$$

so, to get the answer, that is the corresponding cumulant $u_{t,\Delta t}^{HP'}(x)$ for the model

[1] Stress that this is different from the situation with the term L_4 itself: in L_4, we describe x^H occupied the position at x *before* the event, whereas in $\overline{L}_4'$ both x^{H-} and x^{P+} share the same position *after* the event.

generated by $\overline{L}_4'$, one has to get rid of the singular term. We will do this in the beyond mean-field approach below.[2]

Following the general scheme, we consider $\overline{L}_\varepsilon$ with $a^\pm(x-y)$ and $b(x-y)$ above replaced by $\varepsilon^d a^\pm(\varepsilon x - \varepsilon y)$ and $\varepsilon^d b(\varepsilon x - \varepsilon y)$, respectively; next, we consider the dynamics of $\overline{\gamma}_{\varepsilon,t}$ defined by (2.18) with $\overline{L}$ replaced by $\overline{L}_\varepsilon$. Let $q^X_{\Delta t}$, $g^{X,Y}_{\Delta t}$, $X, Y \in \{HO,\ H+,\ H-,\ PO,\ P+,\ P-\}$ be the corresponding functions from the beyond mean-field expansion. We consider, cf. (2.32), for all $A, B \in \{H, P\}$,

$$\widetilde{g}^{AB}_{t,\Delta t}(\xi) := \widetilde{g}^{AO,BO}_{\Delta t}(\xi) + \widetilde{g}^{AO,B+}_{\Delta t}(\xi) + \widetilde{g}^{A-,BO}_{\Delta t}(\xi) + \widetilde{g}^{A-,B+}_{\Delta t}(\xi), \tag{4.24}$$

and, cf. (2.38),

$$\begin{aligned} h^{HH}_{t,\Delta t}(\xi) &= \widetilde{g}^{HH}_{t,\Delta t}(\xi) + q^{HO}_{\Delta t}; \qquad & h^{HP}_{t,\Delta t}(\xi) &= \widetilde{g}^{HP}_{t,\Delta t}(\xi); \\ h^{PH}_{t,\Delta t}(\xi) &= \widetilde{g}^{PH}_{t,\Delta t}(\xi); \qquad & h^{PP}_{t,\Delta t}(\xi) &= \widetilde{g}^{PP}_{t,\Delta t}(\xi) + q^{PO}_{\Delta t}. \end{aligned} \tag{4.25}$$

Recall that, $\widetilde{g}^{HP}_{t,\Delta t} \neq \widetilde{g}^{PH}_{t,\Delta t}$ and hence $h^{HP}_{t,\Delta t} \neq h^{PH}_{t,\Delta t}$.

By (4.23), $g^{HP}_{t,\Delta t}(x)$ is (the only) non-regular function which should include the Dirac δ function, as a result $\widetilde{g}^{HP}_{t,\Delta t}$ will be non-integrable at infinity. By (4.23), if we subtract from $\widetilde{g}^{HP}_{t,\Delta t}$ its limit at infinity, the result will be nothing but the Fourier transform of $g^{HP'}_{t,\Delta t}(x)$, so that, cf. (2.31),

$$\begin{gathered} u^{AB}_{\varepsilon,t,\Delta t}(x) = \varepsilon^d g^{AB}_{t,\Delta t}(\varepsilon x) + o(\varepsilon^d), \\ AB \in \{HH, HP', PH, PP\}. \end{gathered} \tag{4.26}$$

We will get hence the values of the spatiotemporal cumulants for the initial model.

In Subsection 4.3 below, we will show that

$$\frac{d}{d\Delta t}\begin{pmatrix} h^{HH}_{t,\Delta t}(\xi) \\ h^{HP}_{t,\Delta t}(\xi) \end{pmatrix} = \mathcal{D}_{t,\Delta t}(\xi)\begin{pmatrix} h^{HH}_{t,\Delta t}(\xi) \\ h^{HP}_{t,\Delta t}(\xi) \end{pmatrix} \tag{4.27}$$

$$\frac{d}{d\Delta t}\begin{pmatrix} h^{PH}_{t,\Delta t}(\xi) \\ h^{PP}_{t,\Delta t}(\xi) \end{pmatrix} = \mathcal{D}_{t,\Delta t}(\xi)\begin{pmatrix} h^{PH}_{t,\Delta t}(\xi) \\ h^{PP}_{t,\Delta t}(\xi), \end{pmatrix} \tag{4.28}$$

where $\mathcal{D}_{t,\Delta t}(\xi)$ is the following matrix:

$$\begin{pmatrix} \widetilde{a}^+(\xi) - \big(A^- + \widetilde{a}^-(\xi)\big)\big(q^{HO}_{\Delta t} + q^{H+}_{\Delta t}\big) - B\big(q^{PO}_{\Delta t} + q^{P+}_{\Delta t}\big) & -\widetilde{b}(\xi)\big(q^{HO}_{\Delta t} + q^{H+}_{\Delta t}\big) \\ B\big(q^{PO}_{\Delta t} + q^{P+}_{\Delta t}\big) & \widetilde{b}(\xi)\big(q^{HO}_{\Delta t} + q^{H+}_{\Delta t}\big) - m \end{pmatrix}$$

By (2.33),

$$q^{HO}_{\Delta t} + q^{H+}_{\Delta t} = q^H_{t+\Delta t}, \qquad q^{PO}_{\Delta t} + q^{P+}_{\Delta t} = q^P_{t+\Delta t},$$

[2] It is worth noting that it can be done also in more mathematically rigorous way by considering a δ-sequence of functions which converge to the Dirac δ-function in the sense of distributions; then, in particular, their Fourier transforms converge pointwise to 1, see e.g. [4, Chapter 2].

and hence, cf. (4.4),

$$\mathcal{D}_{t,\Delta t}(\xi) = \begin{pmatrix} \mathtt{a}_{t+\Delta t}(\xi) & \mathtt{b}_{t+\Delta t}(\xi) \\ \mathtt{c}_{t+\Delta t} & \mathtt{d}_{t+\Delta t}(\xi) \end{pmatrix} =: \mathcal{E}_{t+\Delta t}(\xi). \tag{4.29}$$

Therefore, by (4.5), we can represent $\mathcal{A}_{t+\Delta t}(\xi)$ as a block matrix, namely,

$$\mathcal{A}_{t+\Delta t}(\xi) = \begin{pmatrix} \mathcal{E}_{t+\Delta t}(\xi) & \mathbf{0} \\ \mathbf{0} & \mathcal{E}_{t+\Delta t}(\xi), \end{pmatrix} \tag{4.30}$$

where $\mathbf{0}$ denotes 2×2 matrix of zeros, and hence, denoting, cf. (4.9),

$$\overline{h}_{t,\Delta t}(\xi) := \Big(h^{HH}_{t,\Delta t}(\xi), h^{HP}_{t,\Delta t}(\xi), h^{PH}_{t,\Delta t}(\xi), h^{PP}_{t,\Delta t}(\xi) \Big)^T,$$

we get, by (4.27), (4.28),

$$\frac{d}{d\Delta t}\overline{h}_{t,\Delta t}(\xi) = \mathcal{A}_{t+\Delta t}(\xi)\overline{h}_{t,\Delta t}(\xi), \tag{4.31}$$

cf. also (4.10). Note that, by (4.29), cf. (4.8),

$$\mathcal{A}_{t+\Delta t}(\xi) = \mathcal{A}[q^H_{t+\Delta t}, q^P_{t+\Delta t}](\xi).$$

Comparing this with (4.10), we see that the conjecture holds.

One can now solve, for each $\xi \in \mathbb{R}^d$, a linear ordinary differential equation (4.31) with the initial condition

$$\overline{h}_{t,\Delta t}(\xi)\Big|_{\Delta t=0} = \overline{h}_*(\xi),$$

where $\overline{h}_*(\xi)$ is given by (4.20), (4.22). From solution $\overline{h}_{t,\Delta t}(\xi)$ to (4.31), one can get $\widetilde{g}^{AB}_{t,\Delta t}(\xi)$, $A, B \in \{H, P\}$ from (4.25), to this end, one needs $q^{HO}_{\Delta t}$ and $q^{PO}_{\Delta t}$. The latter functions satisfy the following differential equations (see Subsection 4.3):

$$\begin{aligned} \frac{d}{d\Delta t} q^{HO}_{\Delta t} &= -\big(B(q^{PO}_{\Delta t} + q^{P+}_{\Delta t}) + A^-(q^{HO}_{\Delta t} + q^{H+}_{\Delta t})\big) q^{HO}_{\Delta t} \\ &= -\big(B q^P_{t+\Delta t} + A^- q^H_{t+\Delta t}\big) q^{HO}_{\Delta t}, \\ \frac{d}{d\Delta t} q^{PO}_{\Delta t} &= -m q^{PO}_{\Delta t}. \end{aligned} \tag{4.32}$$

The explicit form of functions $\widetilde{g}^{AB}_{t,\Delta t}(\xi)$, $A, B \in \{H, P\}$, can be found in the Appendix, in the proof of the next Proposition.

Proposition 4.6. *Let* ($\mathbf{HP_1}$)–($\mathbf{HP_3}$) *hold. Then functions* $\widetilde{g}^{HH}_{t,\Delta t}(\xi)$, $\widetilde{g}^{PH}_{t,\Delta t}(\xi)$, *and* $\widetilde{g}^{PP}_{t,\Delta t}(\xi)$ *converge to* 0 *as* $|\xi| \to \infty$, *whereas*

$$\widetilde{g}^{HP}_{t,\Delta t}(\infty) := \lim_{|\xi|\to\infty} \widetilde{g}^{HP}_{t,\Delta t}(\xi) \neq 0.$$

Moreover, all functions $\widetilde{g}^{HH}_{t,\Delta t}(\xi)$, $\widetilde{g}^{PH}_{t,\Delta t}(\xi)$, $\widetilde{g}^{PP}_{t,\Delta t}(\xi)$, *and*

$$\widetilde{g}^{HP'}_{t,\Delta t}(\xi) := \widetilde{g}^{HP}_{t,\Delta t}(\xi) - \widetilde{g}^{HP}_{t,\Delta t}(\infty)$$

are integrable.

An explicit formula for $\widetilde{g}^{HP}_{t,\Delta t}(\infty)$ is provided in the Appendix below, see (A.33).

As a result, for each $AB \in \{HH, HP', PH, PP\}$, one can apply the inverse Fourier transform to an integrable function $\widetilde{g}^{AB}_{t,\Delta t}(\xi)$, to get, for a.a. $x \in \mathbb{R}^d$, the needed for (4.26) function $g^{AB}_{t,\Delta t}(x)$.

Theorem 4.7. *Let* $(\mathbf{HP_1})$–$(\mathbf{HP_3})$ *hold.*

1. **(Convergence as $\Delta t \to \infty$)** *Let $AB \in \{HH, HP', PH, PP\}$. Then, for all $t \geq 0$ and $\xi \in \mathbb{R}^d$,*

$$\lim_{\Delta t\to\infty} \widetilde{g}^{AB}_{t,\Delta t}(\xi) = 0. \tag{4.33}$$

2. **(Convergence as $t \to \infty$)**

   (a) *Let $AB \in \{HH, HP', PH, PP\}$. Then $\widetilde{g}^{AB}_{t,\Delta t}(\xi)$ converges pointwise as $t \to \infty$ to an integrable function $\widetilde{g}^{AB}_{\infty,\Delta t}(\xi)$ described below. Moreover, $g^{AB}_{t,\Delta t}(x)$ converge uniformly in a.a. $x \in \mathbb{R}^d$ as $t \to \infty$ to the corresponding inverse Fourier transform $g^{AB}_{\infty,\Delta t}(x)$.*

   (b) *For all $\Delta t \geq 0$ and $\xi \in \mathbb{R}^d$,*

$$\begin{aligned}
\widetilde{g}^{HH}_{\infty,\Delta t}(\xi) &= h^{HH}_{\infty,\Delta t}(\xi) - q^H_* e^{-A^+\Delta t},\\
\widetilde{g}^{PP}_{\infty,\Delta t}(\xi) &= h^{PP}_{\infty,\Delta t}(\xi) - q^P_* e^{-m\Delta t},\\
\widetilde{g}^{PH}_{\infty,\Delta t}(\xi) &= h^{PH}_{\infty,\Delta t}(\xi),\\
\widetilde{g}^{HP'}_{\infty,\Delta t}(\xi) &= h^{HP}_{\infty,\Delta t}(\xi) - h^{HP}_{\infty,\Delta t}(\infty),
\end{aligned} \tag{4.34}$$

   where

$$h^{HP}_{\infty,\Delta t}(\infty) = \begin{cases} \dfrac{mq^P_*}{m - A^+}\big(e^{-A^+\Delta t} - e^{-m\Delta t}\big), & \text{if } A^+ \neq m;\\ mq^P_* \Delta t\; e^{-A^+\Delta t}, & \text{if } A^+ = m. \end{cases} \tag{4.35}$$

   (c) *In the above, $h^{AB}_{\infty,\Delta t}(\xi)$, $A, B \in \{H, P\}$ are the following limits which exist for all $\Delta t \geq 0$ and $\xi \in \mathbb{R}^d$:*

$$\begin{pmatrix} h^{HH}_{\infty,\Delta t}(\xi)\\ h^{HP}_{\infty,\Delta t}(\xi)\end{pmatrix} := \lim_{t\to\infty}\begin{pmatrix} h^{HH}_{t,\Delta t}(\xi)\\ h^{HP}_{t,\Delta t}(\xi)\end{pmatrix} = e^{\Delta t\cdot\mathcal{E}_*(\xi)}\begin{pmatrix} h^{HH}_*(\xi)\\ h^{HP}_*(\xi)\end{pmatrix} \tag{4.36}$$

$$\begin{pmatrix} h^{PH}_{\infty,\Delta t}(\xi)\\ h^{PP}_{\infty,\Delta t}(\xi)\end{pmatrix} := \lim_{t\to\infty}\begin{pmatrix} h^{PH}_{t,\Delta t}(\xi)\\ h^{PP}_{t,\Delta t}(\xi)\end{pmatrix} = e^{\Delta t\cdot\mathcal{E}_*(\xi)}\begin{pmatrix} h^{HP}_*(\xi)\\ h^{PP}_*(\xi)\end{pmatrix}, \tag{4.37}$$

   where, cf. (4.17), the matrix

$$\mathcal{E}_*(\xi) := \lim_{t\to\infty} \mathcal{E}_t(\xi) = \begin{pmatrix} \mathtt{a}_*(\xi) & \mathtt{b}_*(\xi) \\ \mathtt{c}_* & \mathtt{d}_*(\xi) \end{pmatrix} \tag{4.38}$$

has negative real parts of both eigenvalues, for each $\xi \in \mathbb{R}^d$*; and functions* $h_*^{AB}(\xi)$*,* $A, B \in \{H, P\}$*, are given by* (4.22).

As a result, we obtained the desired leading terms $g^{AB}_{\infty,\Delta t}(x)$ in the beyond mean-field expansion (4.26), for each $AB \in \{HH, HP', PH, PP\}$, of the spatiotemporal cumulant of the considered model in the stationary regime, i.e. when t in (4.26) is replaced by ∞.

4.3 Derivation of equations

In this Subsection, we are going to derive, in particular, equations (4.3), (4.10), and (4.31). Similarly to Subsection 4.3, we will partially use the Model Constructor toolbox from [1]. Firstly, we note that the operators L_1, L_2, L_3, L_4 defined above represent `Birth`, `DeathByCompetition`, `DensityIndependentDeath` and `Infection` components, respectively.

Next, we express $\overline{L}_1, \overline{L}_2, \overline{L}_3, \overline{L}_4$ given above as sums of *model components* in the terminology of [1, Supplementary Note 2]. Namely

$$\overline{L}_1 = L_{11} + L_{12}, \qquad \overline{L}_2 = L_{21} + L_{22} + L_{23} + L_{24},$$
$$\overline{L}_3 = L_{31} + L_{32}, \qquad \overline{L}_4 = L_{41} + L_{42} + L_{43} + L_{44}.$$

Here L_{11} represents the `Birth` component:

$$(L_{11}F)(\overline{\gamma}) = \sum_{x\in\gamma^{H+}} \int_{\mathbb{R}^d} a^+(x-y)\Big(F(\gamma^{HO}, \gamma^{H+} \cup \{y\}, \gamma^{H-}, \overline{\gamma}^P) - F(\overline{\gamma})\Big)\, dy;$$

L_{12} represents the `BirthToAnotherType` component:

$$(L_{12}F)(\overline{\gamma}) = \sum_{x\in\gamma^{HO}} \int_{\mathbb{R}^d} a^+(x-y)\Big(F(\gamma^{HO}, \gamma^{H+} \cup \{y\}, \gamma^{H-}, \overline{\gamma}^P) - F(\overline{\gamma})\Big)\, dy;$$

L_{21} represents the `DeathByCompetition` component:

$$(L_{21}F)(\overline{\gamma}) = \sum_{x\in\gamma^{H+}} \sum_{y\in(\gamma^{H+}\setminus\{x\})} a^-(x-y)\Big(F(\gamma^{HO}, \gamma^{H+} \setminus \{x\}, \gamma^{H-}, \overline{\gamma}^P) - F(\overline{\gamma})\Big)\Big)$$

L_{22} represents the `DeathByExternalFactor` component:

$$(L_{22}F)(\overline{\gamma}) = \sum_{x\in\gamma^{H+}} \sum_{y\in\gamma^{HO}} a^-(x-y)\Big(F(\gamma^{HO}, \gamma^{H+} \setminus \{x\}, \gamma^{H-}, \overline{\gamma}^P) - F(\overline{\gamma})\Big)\Big)$$

L_{23} and L_{24} both represent the `ChangeInTypeByFacilitation` component introduced in Subsection 4.3:

$$(L_{23}F)(\overline{\gamma}) = \sum_{x\in\gamma^{HO}} \sum_{y\in(\gamma^{HO}\setminus\{x\})} a^{-}(x-y)\Big(F(\gamma^{HO}\setminus\{x\}, \gamma^{H+}, \gamma^{H-}\cup\{x\}, \overline{\gamma}^{P}) - F(\overline{\gamma})\Big);$$

$$(L_{24}F)(\overline{\gamma}) = \sum_{x\in\gamma^{HO}} \sum_{y\in\gamma^{H+}} a^{-}(x-y)\Big(F(\gamma^{HO}\setminus\{x\}, \gamma^{H+}, \gamma^{H-}\cup\{x\}, \overline{\gamma}^{P}) - F(\overline{\gamma})\Big);$$

L_{31} represents the `DensityIndependentDeath` component:

$$(L_{31}F)(\overline{\gamma}) = m\sum_{x\in\gamma^{P+}} \Big(F(\overline{\gamma}^{H}, \gamma^{PO}, \gamma^{P+}\setminus\{x\}, \gamma^{P-}) - F(\overline{\gamma})\Big);$$

L_{32} represents the `ChangeInType` component:

$$(L_{32}F)(\overline{\gamma}) = m\sum_{x\in\gamma^{PO}} \Big(F(\overline{\gamma}^{H}, \gamma^{PO}\setminus\{x\}, \gamma^{P+}, \gamma^{P-}\cup\{x\}) - F(\overline{\gamma})\Big);$$

L_{41} also represents the `ChangeInTypeByFacilitation` component:

$$(L_{41}F)(\overline{\gamma}) = \sum_{x\in\gamma^{H+}} \sum_{y\in\gamma^{PO}} b(x-y)\Big(F(\gamma^{HO}, \gamma^{H+}\setminus\{x\}, \gamma^{H-}, \gamma^{PO}, \gamma^{P+}\cup\{x\}, \gamma^{P-}) - F(\overline{\gamma})\Big);$$

L_{42} represents the `Infection` component (which is, actually, a partial case of the `ChangeInTypeByFacilitation` component):

$$(L_{42}F)(\overline{\gamma}) = \sum_{x\in\gamma^{H+}} \sum_{y\in\gamma^{P+}} b(x-y)\Big(F(\gamma^{HO}, \gamma^{H+}\setminus\{x\}, \gamma^{H-}, \gamma^{PO}, \gamma^{P+}\cup\{x\}, \gamma^{P-}) - F(\overline{\gamma})\Big).$$

Finally, L_{43} and L_{44} both represent the `ChangeInTypeAndBirthByFacilitation` component defined below:

$$\begin{aligned}(L_{43}F)(\overline{\gamma}) = &\sum_{x\in\gamma^{HO}} \sum_{y\in\gamma^{PO}} b(x-y)\\ &\times\int_{\mathbb{R}^d} \delta(x-z)\Big(F(\gamma^{HO}\setminus\{x\}, \gamma^{H+}, \gamma^{H-}\cup\{x\}, \gamma^{PO}, \gamma^{P+}\cup\{z\}, \gamma^{P-}) - F(\overline{\gamma})\Big)\,dz;\\ (L_{44}F)(\overline{\gamma}) = &\sum_{x\in\gamma^{HO}} \sum_{y\in\gamma^{P+}} b(x-y)\\ &\times\int_{\mathbb{R}^d} \delta(x-z)\Big(F(\gamma^{HO}\setminus\{x\}, \gamma^{H+}, \gamma^{H-}\cup\{x\}, \gamma^{PO}, \gamma^{P+}\cup\{z\}, \gamma^{P-}) - F(\overline{\gamma})\Big)\,dz.\end{aligned}$$

We are going to describe the Wolfram Mathematica code we used. First six lines (In[1]–In[6]) are the same as in Subsection 3.3: we load libraries, set-up internal variables, define the `ChangeInTypeByFacilitation` and `Relax` components.

Next, we define the `ChangeInTypeAndBirthByFacilitation` model component needed for L_{43} and L_{44} above. It describes the event when an agent at a position x_2 changes own type from s_3 to s_2 and, simultaneously, it sends an off-spring of a

type s_1 to a position x_1. The off-spring is sent through a kernel $d(x_1 - x_2)$ (which is the Dirac δ-function $\delta(x_1 - x_2)$ in operators L_{43} and L_{44}). The event happened because of interaction of the agent with each of other agents of a type s_4 placed at a position x_3. The interaction is given through a kernel $b(x_2 - x_3)$. In particular, s_4 may be equal to s_1 as it is needed for L_{44}.

```
In[7]:= ChangeInTypeAndBirthByFacilitation[s1_,s2_,s3_,s4_,b_,Bf_,
                                            d_,Df_,Coeffic_]:=
          Module[{Products={{s1,x1}, {s2,x2}},
                 Reactants={{s3,x2}},Catalysts={{s4,x3}},
                 listAll,function,Interactions,name},
                 listAll={Products,Reactants,Catalysts};
                 function[x1_,x2_,x3_]:=b[x2-x3]d[x1-x2];
                 Interactions={{b,Bf,x2,x3},{d,Df,x1,x2}};
                 name="ChangeInTypeAndBirthByFacilitation";
                 {listAll,function,Interactions,name,Coeffic}];
```

We define now the `SpatialProcess` which includes all model components corresponding to operators L_1, L_2, L_3, L_4. Henceforth, for the spatial process, the agent types $1, 2$ correspond to H, P, respectively.

```
In[8]:= SpatialProcess={Birth[1,a^+,ã^+,1],
                  DeathByCompetition[1,a^-,ã^-,1],
                  DensityIndependentDeath[2,m,1],
                  Infection[2,1,b,b̃,1]};
```

Next, we define the `AuxiliaryProcess` which includes all model components corresponding to operators L_{ij} introduced above. Henceforth, for the auxiliary process, the agent types $1, 2, 3, 4, 5, 6$ correspond to $HO, H+, H-, PO, P+, P-$, respectively.

```
In[9]:= AuxiliaryProcess={
         Birth[2,a^+,ã^+,1],(*L11*)
         BirthToAnotherType[2,1,a^+,ã^+,1],(*L12*)
         DeathByCompetition[2,a^-,ã^-,1],(*L21*)
         DeathByExternalFactor[2,1,a^-,ã^-,1],(*L22*)
         ChangeInTypeByFacilitation[3,1,1,a^-,ã^-,1],(*L23*)
         ChangeInTypeByFacilitation[3,1,2,a^-,ã^-,1],(*L24*)
         DensityIndependentDeath[5,m,1],(*L31*)
         ChangeInType[6,4,m,1],(*L32*)
         ChangeInTypeByFacilitation[5,2,4,b,b̃,1],(*L41*)
         Infection[5,2,b,b̃,1],(*L42*)
         ChangeInTypeAndBirthByFacilitation[5,3,1,4,b,b̃,d,d̃,1],(*L43*)
         ChangeInTypeAndBirthByFacilitation[5,3,1,5,b,b̃,d,d̃,1],(*L44*)
         Relax[3], Relax[6] (*No dynamics of '-' agents*)};
```

(Note that the δ-function here is denoted by `d`.)

To simplify the representation of the calculations below, we introduce a replacement rule to replace values of the Fourier transform at the origin by the corresponding integral, i.e.

$$\widetilde{a}^{\pm}(0) = A^{\pm}, \qquad \widetilde{b}(0) = B;$$

we include here also the replacement $\widetilde{\delta} \equiv 1$:

```
In[10]:=  integrals={ã⁺[0]→A⁺,ã⁻[0]→A⁻,b̃[0]→B,d̃[0]→1,d̃[ξ]→1};
```

In the Model Constructor toolbox, `HQfALL` and also `HGfALL` are the functions providing the r.h.s. of the differential equations for functions q and g, respectively.

We define now a function which represents the r.h.s. of the corresponding mean-field equation on function q, cf. (1.25), for an agent of a type `i` under the replacement rule above:

```
In[11]:=  qEqn[process_,i_]:=HQfALL[qpgVariables,process,i]/.integrals;
```

We define also a function which represents the *sum* of the r.h.s. of equations for functions g, cf. (1.30), between pairs of types from a list:

```
In[12]:=  gEqns[process_,list_]:=Total[Apply[HGfALL[qpgVariables,
                     process,#1,#2,kVariable]&,#]&/@list]/.integrals;
```

We obtain the r.h.s. of the differential equation for functions h_t^{AB}, $A, B \in \{H, P\}$ defined by (4.2).

```
In[13]:=  qgEqnSpatial={qEqn[#,1]+gEqns[#,{{1,1}}],
                     gEqns[#,{{1,2}}],gEqns[#,{{2,1}}],
                     qEqn[#,2]+gEqns[#,{{2,2}}]
                    }&[SpatialProcess];
```

We obtain the r.h.s. of the differential equation for functions $h_{t,\Delta t}^{AB}$, $A, B \in \{H, P\}$ defined by (4.25):

```
In[14]:=  qgEqnAuxiliary={qEqn[#,1]+gEqns[#,{{1,1},{1,2},{1,3},{2,3}}],
                       gEqns[#,{{1,4},{1,5},{3,4},{3,5}}],
                       gEqns[#,{{1,4},{1,6},{2,4},{2,6}}],
                       qEqn[#,4]+gEqns[#,{{4,4},{4,5},{4,6},{5,6}}]
                      }&[AuxiliaryProcess];
```

Since are interested in the equations for h, we create a replacement rule to rewrite g through h, cf. (4.2):

```
In[15]:=  hReplaceSpatial={g[1,1,ξ]→h^HH-q[1],g[1,2,ξ]→h^HP,
                       g[2,2,ξ]→h^PP-q[2]};
```

We do the same for the auxiliary process, however now the replacement rule contains sum of different g, cf. (4.24) and (4.25):

```
In[16]:= hReplaceAuxiliary={g[1,1,ξ]+g[1,2,ξ]+g[1,3,ξ]+g[2,3,ξ]→h^HH-q[1],
                           g[1,4,ξ]+g[1,5,ξ]+g[3,4,ξ]+g[3,5,ξ]→h^HP,
                           g[1,4,ξ]+g[1,6,ξ]+g[2,4,ξ]+g[2,6,ξ]→h^PH,
                           g[4,4,ξ]+g[4,5,ξ]+g[4,6,ξ]+g[5,6,ξ]→h^PP-q[4]};
```

We obtain now the mean-field equations (4.3):

```
In[17]:= {qEqn[SpatialProcess,1],qEqn[SpatialProcess,2]}//Factor

Out[17]= {-q[1] (B q[2]+q[1] A^- -A^+),-(m-B q[1]) q[2]}
```

We find now all stationary solutions to the system (4.3):

```
In[18]:= qStationary={q[1],q[2]}/.Solve[{qEqn[SpatialProcess,1]==0,
                                 qEqn[SpatialProcess,2]==0},{q[1],q[2]}]
```

Out[18]= $\{\{\frac{m}{B},-\frac{m\ A^{-}-B\ A^{+}}{B^{2}}\},\{0,0\},\{\frac{A^{+}}{A^{-}},0\}\}$

We save values of the only pair (4.11) of positive stationary solutions to (4.3):

```
In[19]:= {qH,qP}=First[qStationary];
```

We find the r.h.s. of the differential equations (4.32):

```
In[20]:= {qEqn[AuxiliaryProcess,1],qEqn[AuxiliaryProcess,4]}//Factor

Out[20]= {-q[1] (B q[4]+B q[5]+q[1] A^- +q[2] A^-),-m q[4]}
```

We are going to derive/verify now equation (4.10). We create a vector of the notations for functions h_t^{AB}, $A, B \in \{H, P\}$. Note that the second and the third component are denoted identically as the corresponding functions are equal:

```
In[21]:= hbarSpatial={h^HH,h^HP,h^HP,h^PP};
```

We replace now the terms with functions g in the equations for h_t^{AB} obtained in In[13] by the corresponding notations:

```
In[22]:= hEqnSpatial=Expand[qgEqnSpatial/.hReplaceSpatial];
```

We define the coefficients (4.4) of matrix (4.5).

```
In[23]:= at=ã^+[ξ]- (A^- +ã^-[ξ])q[1]-B q[2];
         bt=-q[1] b̃[ξ]; ct=B q[2]; dt=q[1] b̃[ξ]-m;
```

We define matrices (4.5) and (4.6):

```
In[27]:=  matrA={{at,bt,0,0},{ct,dt,0,0},{0,0,at,bt},{0,0,ct,dt}};
          matrAprime={{at,bt,0,0},{0,0,at,bt},{ct,dt,0,0},{0,0,ct,dt}};
```

We define vector (4.7):

```
In[29]:=  vecB={A⁺q[1] +A⁻ q[1]^2 ,0,0,m q[2]}+B q[1] q[2]{1,-1,-1,1};
```

We verify now equation (4.10):

```
In[30]:=  (matrA+matrAprime).hbarSpatial+vecB==hEqnSpatial//FullSimplify

Out[30]=  True
```

We are going to verify now equations (4.27), (4.28), (4.31). Similarly to above, we define a vector of notations for the functions $h^{AB}_{t,\Delta t}$, $A, B \in \{H, P\}$. Note that the second and the third component are different now:

```
In[31]:=  hbarAuxiliary={h^HH,h^HP,h^PH,h^PP};
```

The r.h.s. of the equations for $h^{AB}_{t,\Delta t}$ obtained in In[14] are linear combinations of various $g[i, k, \xi]$. One needs some preparation to rearrange the terms there to separate the sums of g corresponding to h.

Firstly, we create a collection rule:

```
In[32]:=  collectRule={B,-B ,b̃[ξ] ,-b̃[ξ],ã⁺[ξ],ã⁻[ξ],-ã⁻[ξ],
                      A⁻,-A⁻,m,-m,-q[1] ,-q[2],-q[4],-q[5],
                      q[1],q[2],q[3],q[4],q[5]};
```

Next, we collect the terms in the r.h.s. of the equations obtained in In[14] according to the rule above; and then one can replace the sums of g by the corresponding notations for $h^{AB}_{t,\Delta t}$. Then we expand, and thereafter we collect again, now to distinguish the coefficients before functions h:

```
In[33]:=  hEqnAuxiliary=Collect[Expand[Collect[qgEqnAuxiliary,
                                   collectRule]/.hReplaceAuxiliary],
                      Join[hbarAuxiliary,collectRule]];
```

We verify now equations (4.27) and (4.28) by funding the matrix $\mathcal{D}_{t,\Delta t}$:

```
In[34]:=  hEqnAuxiliary[[1;;2]]

Out[34]=  {-h^HP (q[1]+q[2]) b̃[ξ]
            +h^HH (-B (q[4]+q[5])-(q[1]+q[2]) A⁻-(q[1]+q[2]) ã⁻[ξ]+ã⁺[ξ]),
            Bh^HH (q[4]+q[5])+h^HP (-m+(q[1]+q[2]) b̃[ξ])}
```

and also

```
In[35]:= hEqnAuxiliary[[3;;4]]

Out[35]= {-h^PP (q[1]+q[2]) b̃[ξ]
            +h^PH (-B (q[4]+q[5])-(q[1]+q[2]) A^- -(q[1]+q[2]) ã^-[ξ]+ã^+[ξ]),
            B h^PH (q[4]+q[5])+h^PP (-m+(q[1]+q[2]) b̃[ξ])}
```

We verify now equation (4.31):

```
In[36]:= (hEqnAuxiliary/.{q[1]+q[2]→q[1],q[4]+q[5]→q[2]})
                ==matrA.hbarAuxiliary//FullSimplify

Out[36]= True
```

We are going now to define the functions in the stationary regime, using the replacement according to (4.12). Firstly, we consider the stationary version (the limit as $t \to \infty$) of the matrix (4.5):

```
In[37]:= matrAst=matrA/.{q[1]→qH,q[2]→qP}//FullSimplify;
```

Then we define vector (4.19):

```
In[38]:= vecBst=vecB/.{q[1]→qH,q[2]→qP}//FullSimplify;
```

We define also the coefficients (4.17) of matrix $\mathcal{A}_*$, i.e. just the limits of (4.4):

```
In[39]:= ast=matrAst[[1,1]]; bst=matrAst[[1,2]];
         cst=matrAst[[2,1]]; dst=matrAst[[2,2]];
```

Then we define matrix $\mathcal{C}_*$, cf. (4.18):

```
In[43]:= matrC={{2ast,2bst,0},{cst,ast+dst,bst},{0,2cst,2dst}};
```

Therefore, one can obtain $h_*^{HH}(\xi), h_*^{HP}(\xi), h_*^{PP}(\xi)$ from (4.22):

```
In[44]:= {hHHst,hHPst,hPPst}=Inverse[matrC].(Delete[-vecBst,3])//FullSimplify;
```

We use now matrix $\mathcal{A}_*$ to get matrix $\mathcal{E}_*$, cf. (4.30) and also (A.25) below:

```
In[45]:= matrE=matrAst[[1;;2,1;;2]];
```

We can obtain now functions $h_{\infty,\Delta t}^{AB}(\xi)$, $A, B \in \{H, P\}$ from equations (4.36) and (4.37):

```
In[46]:= {hHH,hHP}=MatrixExp[t matrE].{hHHst,hHPst};
         {hPH,hPP}=MatrixExp[t matrE].{hHPst,hPPst};
```

Finally, we are going to verify the corrections obtained in (4.34)–(4.35) to get integrable functions. We were actually interested at the values of the obtained solutions $h_{\infty,\Delta t}^{AB}(\xi)$ as $|\xi| \to \infty$. By the Riemann–Lebesgue lemma, the Fourier transforms of $\widetilde{a}^{\pm}$, $\widetilde{b}$ converge to zero at infinity.

We create the corresponding replacement rule:

```
In[48]:= atInfinity={b̃[ξ]→0,ã⁻[ξ]→0,ã⁺[ξ]→0};
```

We get now the limits of $h^{AB}_{\infty,\vartriangle t}(\xi)$ at infinity:

```
In[49]:= hInfinity={hHH,hHP,hPH,hPP}/.atInfinity//FullSimplify
```

$$\text{Out[49]=}\ \left\{\frac{e^{-t\ A^+}\ m}{B},\frac{2\ e^{-\frac{1}{2}\ t\ (m+A^+)}\ m\ \text{Sinh}[\frac{1}{2}\ t\ (m-A^+)]\ (-m\ A^-+B\ A^+)}{B^2\ (m-A^+)},\ 0,\frac{e^{-m\ t}\ (-m\ A^-+B\ A^+)}{B^2}\right\}$$

Finally, we verify the correction in the limiting case $A^+ = m$:

```
In[50]:= hInfinityBalance=Limit[hInfinity,m→A⁺]
```

$$\text{Out[50]=}\ \left\{\frac{e^{-t\ A^+}\ A^+}{B},\frac{e^{-t\ A^+}\ t\ (B-A^-)\ (A^+)^2}{B^2},0,\frac{e^{-t\ A^+}\ (B-A^-)\ A^+}{B^2}\right\}$$

4.4 Analysis and numerics for the stationary regime

By Theorem 4.7, the eigenvalues of the matrix $\mathcal{E}_*(\xi)$ have negative real parts for each $\xi \in \mathbb{R}^d$. Then, by (4.36)–(4.37), functions $h^{AB}_{\infty,t,\vartriangle t}(\xi)$, $A, B \in \{H, P\}$ converge (for each ξ) to 0 as $\vartriangle t \to \infty$. Similarly to Proposition 4.1, this convergence to 0 can be monotone or oscillating with damping, depending on whether the eigenvalues of $\mathcal{E}_*(\xi)$ are real negative or complex with negative real parts, respectively. By (4.34) and (4.35), functions $\widetilde{g}^{AB}_{\infty,\vartriangle t}(\xi)$, $AB \in \{HH, HP', PH, PP\}$ have the same properties.

By the proof of Theorem 4.7, for a fixed $\xi \in \mathbb{R}^d$, the eigenvalues of $\mathcal{E}_*(\xi)$ are real negative iff

$$z_*(\xi) = \big(A^+ - \widetilde{J}^H_*(\xi) - q^H_*\big(B - \widetilde{b}(\xi)\big)\big)^2 - 4mq^P_*\widetilde{b}(\xi) \tag{4.39}$$

is non-negative; otherwise, they are complex with negative real parts.

The character of convergence hence depends on an interplay between behavior of the Fourier transforms $\widetilde{a}^+(\xi)$, $\widetilde{a}^-(\xi)$ and $\widetilde{b}(\xi)$ in different zones of $\xi \in \mathbb{R}^d$, and is especially non-trivial if the Fourier transforms take negative values, that may be the case e.g. for the Gaussian-like kernels considered in (A.18) in the Appendix.

One can make, however, several general observations about the mentioned convergence.

1. Since
$$z_*(0) = \frac{m^2}{B^2}(A^-)^2 - 4m\Big(A^+ - \frac{m}{B}(A^-)\Big),$$
we conclude that the convergence is oscillating for $\xi = 0$ if and only if (4.13) holds. By continuity and the strict inequality in (4.13), the oscillations will take place for ξ at a neighbourhood of the origin.

2. Directly from (4.39), one gets that the convergence is monotone for all $\xi \in \mathbb{R}^d$ such that $\widetilde{b}(\xi) \le 0$. We assume now that this is not the case and

$$\widetilde{b}(\xi) > 0, \qquad \xi \in \mathbb{R}^d. \tag{4.40}$$

3. To have oscillating convergence for *all large enough* $|\xi|$, one needs with necessity, by the Riemann–Lebesgue lemma, that

$$0 \ge \lim_{|\xi|\to\infty} z_*(\xi) = (A^+ - m)^2,$$

i.e.

$$A^+ = m. \tag{4.41}$$

As a result, to have oscillating in neighbourhoods of the origin and infinity, we require, with necessity that both (4.40), (4.41), and (4.13) hold. Note also that (4.13) under assumption (4.41) can be easily rewritten as follow:

$$B > \frac{1+\sqrt{2}}{2} A^-. \tag{4.42}$$

We assume now that both (4.40)–(4.42) hold; moreover, we also consider the case of equal shapes for all kernels:

$$\begin{aligned} a^\pm(x) = A^\pm c(x) \ge 0, &\qquad b(x) = Bc(x) \ge 0, \\ \int_{\mathbb{R}^d} c(x)\,dx = 1, &\qquad \widetilde{c}(\xi) > 0, \quad \xi \in \mathbb{R}^d. \end{aligned} \tag{4.43}$$

Then, since, by (3.16), $\widetilde{c}(\xi) \le 1$, one can easily rewrite (4.39) as follows:

$$\begin{aligned} z_*(\xi) &= \widetilde{c}(\xi)\biggl(\Bigl(\frac{mA^-}{B}\widetilde{c}(\xi)\Bigr)^2 - 4m\Bigl(m - \frac{mA^-}{B}\Bigr)\biggr) \\ &\le m^2\biggl(\Bigl(\frac{A^-}{B}\Bigr)^2 - 4\Bigl(1 - \frac{A^-}{B}\Bigr)\biggr) < 0, \end{aligned}$$

because of (4.42). Therefore, (4.40)–(4.42) imply that that all functions $\widetilde{g}^{AB}_{\infty,\Delta t}(\xi)$, $AB \in \{HH, HP', PH, PP\}$, converge to 0 as $\Delta t \to \infty$ with damping oscillations for all $\xi \in \mathbb{R}^d$.

Note that the opposite to (4.42) inequality does not imply that the convergence is monotone *for all* $\xi \in \mathbb{R}^d$. Moreover, one can not show analytically that the oscillations will be preserved for the inverse Fourier transforms $g^{AB}_{\infty,\Delta t}(x)$, $AB \in \{HH, HP', PH, PP\}$.

Instead, we are going to show this numerically, for the 2-dimensional case: $d = 2$, and for radially symmetric kernels with equal Gaussian shapes: (4.43) holds with

$$c(x) = \frac{1}{2\pi} e^{-\frac{|x|^2}{2}}, \qquad x \in \mathbb{R}^2. \tag{4.44}$$

We proceed as follows:

- by (4.38), (4.17), we find $e^{\Delta t \cdot \mathcal{E}_*(\xi)}$ (see also below for details);
- by (4.22), we find $h_*^{AB}(\xi)$, $A, B \in \{H, P\}$;
- by (4.36)–(4.37), we find $h_{\infty,\Delta t}^{AB}(\xi)$, $A, B \in \{H, P\}$;
- by (4.34)–(4.35), we find $\widetilde{g}_{\infty,\Delta t}^{AB}(\xi)$, $AB \in \{HH, HP', PH, PP\}$;
- we find numerically $g_{\infty,\Delta t}^{AB}(x)$, $AB \in \{HH, HP', PH, PP\}$.

Firstly, we define a replacement rule to replace both $\widetilde{a}^{\pm}(\xi)$ and $\widetilde{b}(\xi)$ by $A^{\pm}e^{-\frac{1}{2}|\xi|^2}$ and $Be^{-\frac{1}{2}|\xi|^2}$, respectively. Note that we use here $e^{-\frac{1}{2}|\xi|^2}$ instead of the corresponding Fourier transform $e^{-2\pi^2|\xi|^2}$ of the function (4.44), as we are going to use formula (3.32) for the inverse Fourier transform latter on.

```
In[43]:=  equalGaussian={ã⁺[ξ]→A⁺Exp[-ξ²/2],ã⁻[ξ]→A⁻Exp[-ξ²/2],
              b̃[ξ]→B Exp[-ξ²/2]};
```

Next, recall that matrix $\mathcal{E}_*(\xi)$ has either negative real eigenvalues or complex eigenvalues with negative real parts, depending on the value of $\xi \in \mathbb{R}^2$. Depending on this, one gets different expressions for the matrix exponential $e^{\Delta t \cdot \mathcal{E}_*(\xi)}$. More precisely, it is straightforward to check (e.g. with Wolfram Mathematica), that for

$$M = \begin{pmatrix} a & b \\ c & d \end{pmatrix}, \qquad a, b, c, d \in \mathbb{R},$$

its matrix exponential e^{tM} (we use often henceforth t instead of Δt, to simplify notations) can be found by the following formula:

$$e^{tM} = \exp\left(\frac{tx}{2}\right) \begin{pmatrix} \cosh\left(\frac{t\sqrt{z}}{2}\right) + \frac{y \sinh\left(\frac{t\sqrt{z}}{2}\right)}{\sqrt{z}} & \frac{2b \sinh\left(\frac{t\sqrt{z}}{2}\right)}{\sqrt{z}} \\ \frac{2c \sinh\left(\frac{t\sqrt{z}}{2}\right)}{\sqrt{z}} & \cosh\left(\frac{t\sqrt{z}}{2}\right) - \frac{y \sinh\left(\frac{t\sqrt{z}}{2}\right)}{\sqrt{z}} \end{pmatrix},$$

where sinh, cosh denote the hyperbolic sinus and cosine, respectively, and

$$x := \operatorname{tr}(M) = a + d, \qquad y := a - d, \qquad z := x^2 - 4\det(M),$$

where $\det(M) = ad - bc$.

In our case, $M = \mathcal{E}_*(\xi)$, and $z = z_*(\xi) \in \mathbb{R}$, given by (4.39), determines whether the eigenvalues are real or complex. The matrix exponential will have, however, real entries only, as if $z < 0$, then one has

$$\cosh\left(\frac{t\sqrt{z}}{2}\right) = \cosh\left(i\frac{t\sqrt{-z}}{2}\right) = \cos\left(\frac{t\sqrt{-z}}{2}\right), \tag{4.45}$$

$$\frac{\sinh\left(\frac{t\sqrt{z}}{2}\right)}{\sqrt{z}} = \frac{\sinh\left(i\frac{t\sqrt{-z}}{2}\right)}{i\sqrt{-z}} = \frac{i\sin\left(\frac{t\sqrt{-z}}{2}\right)}{i\sqrt{-z}} = \frac{\sin\left(\frac{t\sqrt{-z}}{2}\right)}{\sqrt{-z}}. \tag{4.46}$$

From this, we have also that

$$\lim_{z\to 0} \frac{1}{\sqrt{z}} \sinh\left(\frac{t\sqrt{z}}{2}\right) = \frac{t}{2}, \tag{4.47}$$

regardless of the sign of $z \in \mathbb{R}$. Despite Wolfram Mathematica can handle complex numbers, it may accumulate errors in the imaginary parts in course of the further numerical integration (of the inverse Fourier transform). To avoid this, we introduce auxiliary functions to calculate $e^{\Delta t\cdot\mathcal{E}_*(\xi)}$ depending on the sign of $z = z_*(\xi)$.

We start as follows, because of (4.45)–(4.47):

```
In[44]:=  sinF[t_,z_]:=Switch[Sign[z],1,(1/√z)Sinh[(t√z)/2],
                               -1,(1/√-z)Sin[(t√-z)/2],0,t/2];
          cosF[t_,z_]:=Switch[Sign[z],1,Cosh[(t√z)/2],-1,Cos[(t √-z)/2],0,1];
```

Now, we define the matrix exponential:

```
In[46]:=  expF[t_,x_,y_,z_,b_,c_]:=e^(t x/2){{cosF[t,z]+y sinF[t,z],
            2b sinF[t,z]}, {2 c sinF[t,z], cosF[t,z]-y sinF[t,z]}};
```

Finally, one can define the function to calculate the inverse Fourier transform for the functions $\widetilde{g}^{AB}_{\infty,\Delta t}(\xi)$ defined through (4.34)–(4.35). In the code below:

- `func` calculates the vector of these functions, at the order: HH, HP', PH, PP;
- `hHHstG`, `hHPstG`, `hPPstG` are values of previously found `hHHst`, `hHPst`, `hPPst`, which are functions $h^{HH}_*(\xi)$, $h^{HP}_*(\xi)$, $h^{PP}_*(\xi)$, cf. (4.22), in the case of equal Gaussian kernels;
- `mat` is the previously found matrix `matrE` (which is $\mathcal{E}_*(\xi)$) in the case of equal Gaussian kernels;
- $\mathtt{h}_1, \mathtt{h}_2$ denote *pairs* of functions $\big(h^{HH}_{\infty,\Delta t}(\xi), h^{HP}_{\infty,\Delta t}(\xi)\big)$ and $\big(h^{PH}_{\infty,\Delta t}(\xi), h^{PP}_{\infty,\Delta t}(\xi)\big)$, respectively, found by using (4.36) and (4.37);
- `hAtInf` is the vector of the previously found constants (4.34)–(4.35).

```
In[47]:= gReal[valAp_?NumericQ,valAm_?NumericQ,
             valB_?NumericQ,valm_?NumericQ,valt_?NumericQ,
             valx_?NumericQ]:=
         gReal[valAp,valAm,valB,valm,valt,valx]=Module[{func},
              func[valxi_]=Module[{coeffRule,hHHstG,hHPstG,hPPstG,
                                   x,y,z,b,c,h1,h2,hAtInf,mat,expf},
                coeffRule={A⁺→valAp,A⁻→valAm,B→valB,
                           m→valm,ξ→valxi};
                {hHHstG,hHPstG,hPPstG}={hHHst,hHPst,hPPst}
                                  /.equalGaussian/.coeffRule;
                mat=matrE/.equalGaussian/.coeffRule;
                x=Tr[mat];
                y=mat[[1,1]]-mat[[2,2]];
                z=x^2-4Det[mat];
                b=mat[[1,2]];
                c=mat[[2,1]];
                expf=expF[valt,x,y,z,b,c];
                h1=expf.{hHHstG,hHPstG};
                h2=expf.{hHPstG,hPPstG};
                hAtInf=If[valAp==valm,hInfinityBalance,hInfinity]
                                        /.coeffRule/.t→valt;
                Flatten[{h1,h2}]-hAtInf];
                    (*End of definition of ‘func’*)
          1/(2π) NIntegrate[func[k]k BesselJ[0,k valx],{k,0,3},
                Method →{LocalAdaptive,SymbolicProcessing→0},
                      PrecisionGoal→5]];
```

Remark 4.8. In the last line of the code above, we use formula (3.32) for the inverse Fourier transform. Note that we integrate here in `k` (that is s in (3.32)) from `k` $= 0$ till `k` $= 3$ instead of ∞. The reason is that the whole integrand there becomes extremely small for `k` around and above 3 for all considered values of the parameters $A^{\pm}, B$ and for all considered times Δt (that can be checked by looking at the intermediate computations), and hence the numerical integration may accumulate too many errors.

The result of function `gReal` is the vector of

$$g^{AB}_{\infty,\Delta t}(x), \qquad AB \in \{HH, HP', PH, PP\};$$

here `valt` $= \Delta t$ and `valx` $= |x|$. The result will be used to find the corresponding cumulants by (4.26): the simulations were done for $\varepsilon = \frac{1}{2}$, hence, the cumulants measured in simulations should be approximated as follows:

$$u^{AB}_{\frac{1}{2},\infty,\Delta t}(x) \approx \frac{1}{4} g^{AB}_{\infty,\Delta t}\Big(\frac{x}{2}\Big), \qquad AB \in \{HH, HP', PH, PP\}.$$

The parameters in simulations were chosen as follows:

$$A^+ = 1, \qquad A^- = 0.1, \qquad B = 1, \qquad m = 1, \tag{4.48}$$

that satisfies (4.41) and (4.42).

We are going to discuss now the results of the numerical calculations of the inverse Fourier transforms.

1. For a fixed space variable, e.g. for $x = 0$, the graphs of the g-functions as functions of $\Delta t \in [0, 20]$ are shown on Figure 2a. They demonstrate the damping oscillations. As we can see, values of $g^{HH}_{\infty,\Delta t}(0)$ and $g^{PP}_{\infty,\Delta t}(0)$ are pretty close, as well as values of $g^{HP}_{\infty,\Delta t,\mathrm{reg}}(0)$ and $-g^{PH}_{\infty,\Delta t}(0)$. We observe also the initial negative correlation between finding a host at the current position of a parasite for some positive time interval.

2. Next, in the considered case of monotone kernels, cumulants converge to 0 in $|x|$ monotonically, see Figure 2b for a fixed $\vartriangle t = 7$. In other words, for each $|x| \geq r_0$ (perhaps, staring with some $r_0 > 0$), we will see a picture similar to that on Figure 2a, however, the corresponding cumulants have smaller amplitudes.

3. We consider now the dependence of solutions on A^+ when $A^+ \neq m$. We keep other parameters in (4.48) and compare the graphs of Figure 2a with those for $A^+ = 2 > m$ and $A^+ = \frac{1}{2} < m$. We present the comparison on Figure 3, for e.g. $|x| = 5$. We can observe that, with the growth of A^+, the oscillation became more frequent (the period becomes smaller) and the amplitude becomes higher.

4. Finally, recall that the parameters in (4.48) satisfy (4.42), that is, for $A^+ = m$, nothing but (4.13). We consider the case when (4.42) fails. If we keep $A^+ = B = m = 1$, then (4.42) fails iff $A^- \geq 2(\sqrt{2} - 1) \approx 0.83$. We take $A^- = 0.85$, and then, see Figure 4, the convergence of g-functions in $\vartriangle t$ to 0 becomes monotone, without *visible* damping oscillations. Note that, however, we can not prove this analytically, so the fluctuation may still happen for large values of $\vartriangle t$ and/or $|x|$.

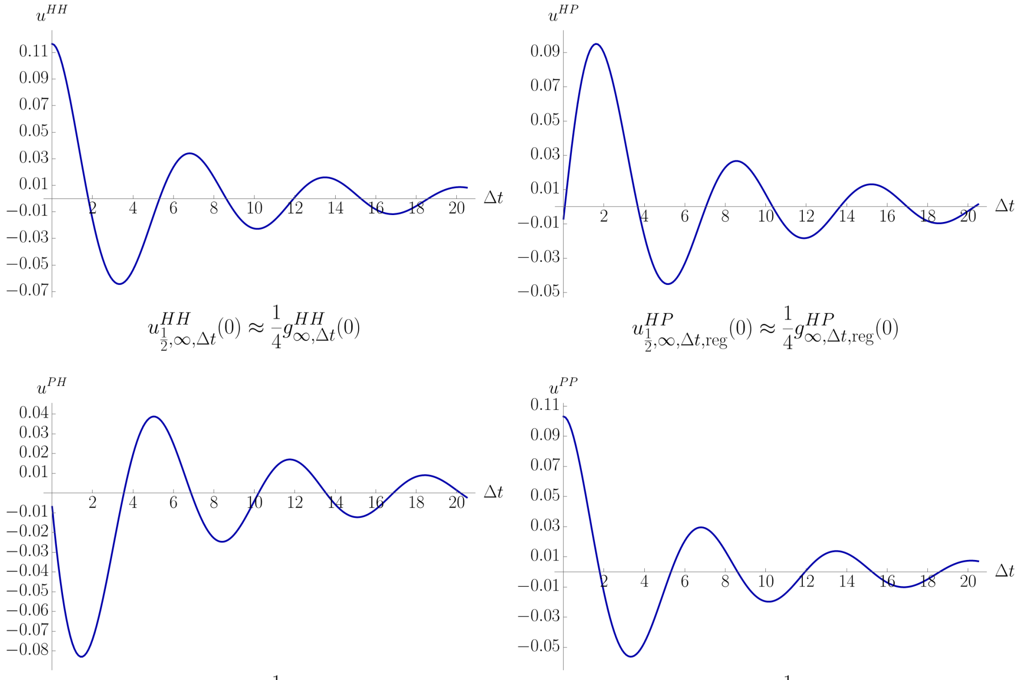

$u^{HH}_{\frac{1}{2},\infty,\Delta t}(0) \approx \frac{1}{4} g^{HH}_{\infty,\Delta t}(0)$ $u^{HP}_{\frac{1}{2},\infty,\Delta t,\mathrm{reg}}(0) \approx \frac{1}{4} g^{HP}_{\infty,\Delta t,\mathrm{reg}}(0)$

$u^{PH}_{\frac{1}{2},\infty,\Delta t}(0) \approx \frac{1}{4} g^{PH}_{\infty,\Delta t}(0)$ $u^{PP}_{\frac{1}{2},\infty,\Delta t}(0) \approx \frac{1}{4} g^{PP}_{\infty,\Delta t}(0)$

(a) Convergence in Δt to 0 with damping oscillation, for the fixed $|x| = 0$

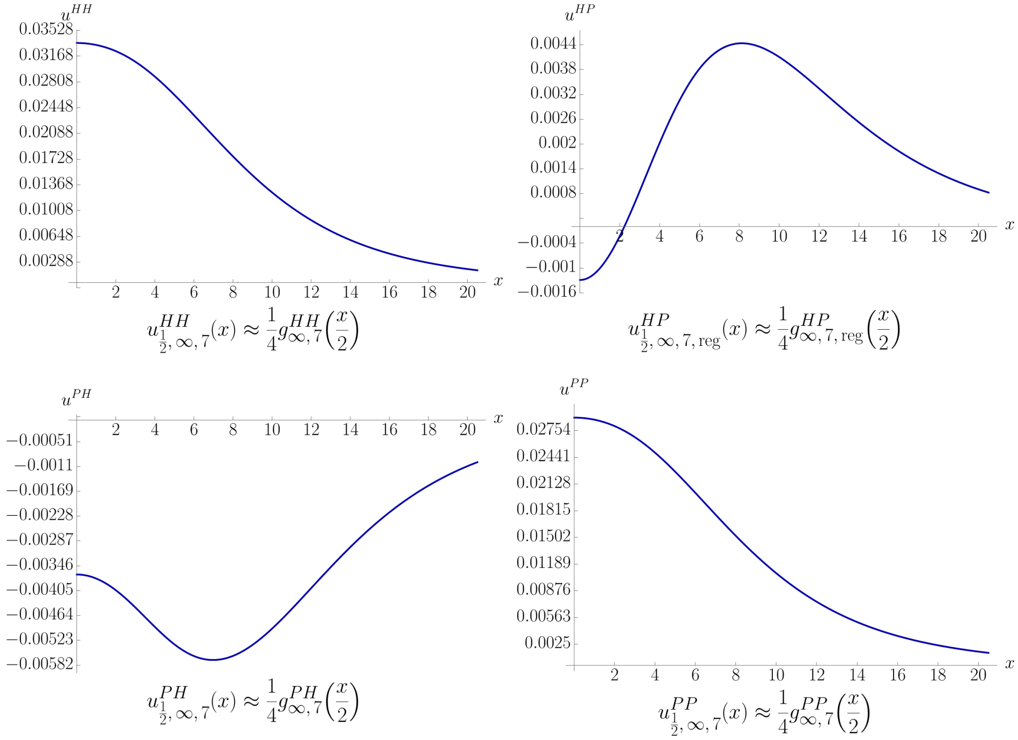

$u^{HH}_{\frac{1}{2},\infty,7}(x) \approx \frac{1}{4} g^{HH}_{\infty,7}\left(\frac{x}{2}\right)$ $u^{HP}_{\frac{1}{2},\infty,7,\mathrm{reg}}(x) \approx \frac{1}{4} g^{HP}_{\infty,7,\mathrm{reg}}\left(\frac{x}{2}\right)$

$u^{PH}_{\frac{1}{2},\infty,7}(x) \approx \frac{1}{4} g^{PH}_{\infty,7}\left(\frac{x}{2}\right)$ $u^{PP}_{\frac{1}{2},\infty,7}(x) \approx \frac{1}{4} g^{PP}_{\infty,7}\left(\frac{x}{2}\right)$

(b) Monotone convergence in $|x|$ to 0, for the fixed $\Delta t = 7$

Figure 2: Behavior of g-functions for $A^+ = m = B = 1$, $A^- = 0.1$

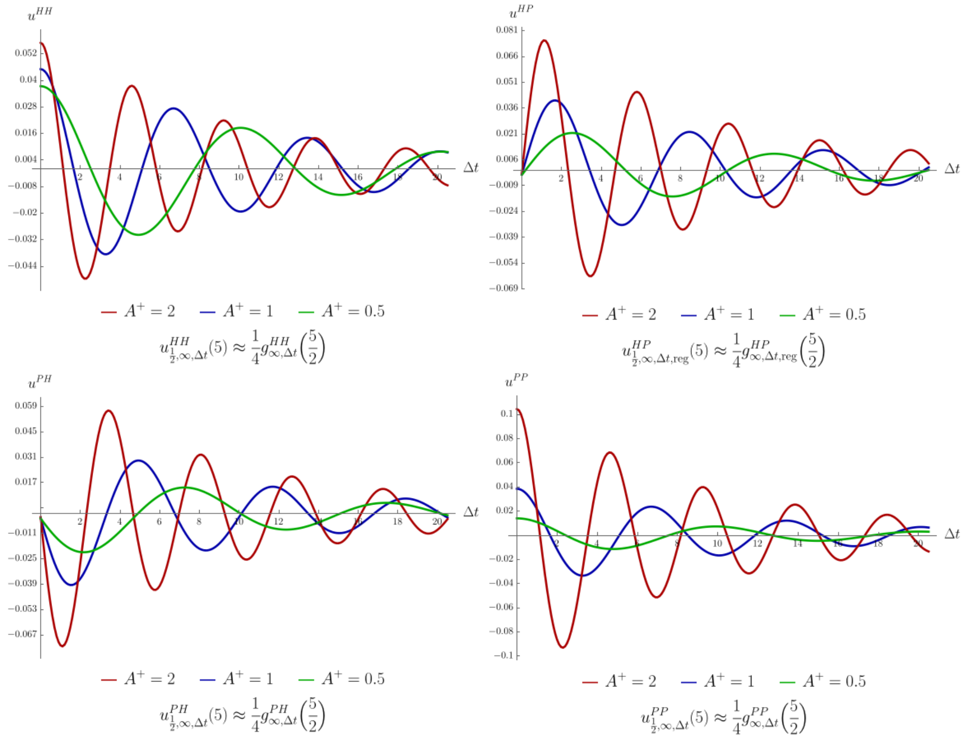

Figure 3: Comparison with different A^+ for $B = m = 1$, $A^- = 0.1$, and $|x| = 5$

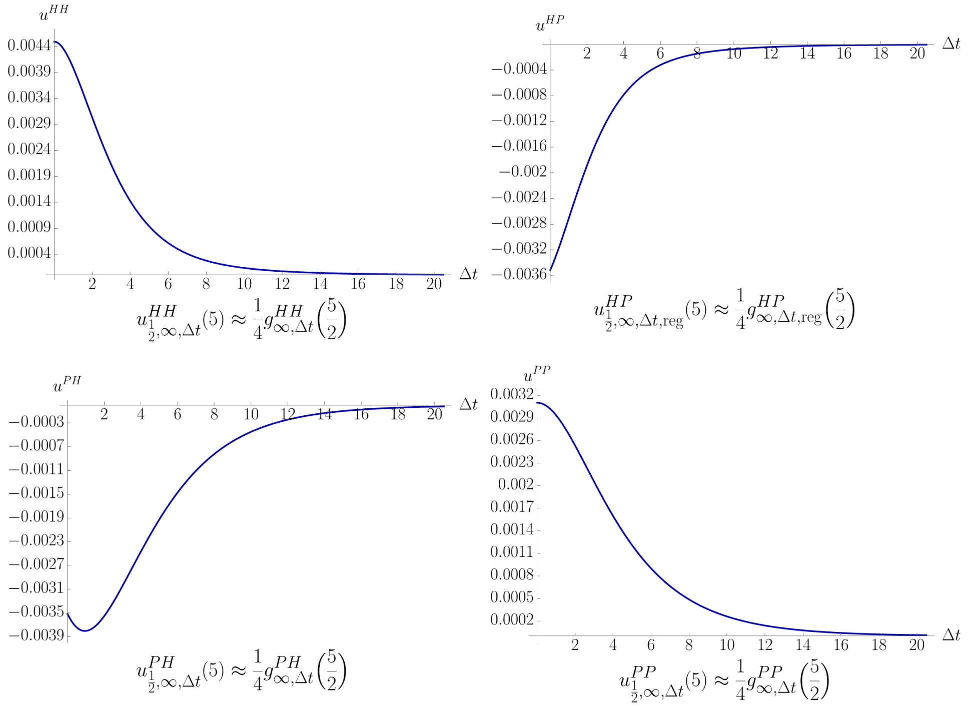

Figure 4: Monotone convergence in Δt to 0 when (4.42)/(4.13) fails:
$A^+ = B = m = 1$, $A^- = 0.85$, and $|x| = 5$

Appendix: Mathematical proofs and discussions

Proof of Theorem 3.2

Step 1. By (2.40),

$$h_{t,0}(\xi) = \widetilde{g}_t(\xi) + q_t.$$

Then, the solution to (3.24) has the form

$$\begin{aligned} h_{t,\vartriangle t}(\xi) &= \exp\biggl(\int_0^{\Delta t} \Bigl(\widetilde{a}^+(\xi) - m - q_{t+\tau}\bigl(\widetilde{a}^-(\xi) + A^-\bigr)\Bigr) d\tau\biggr) h_{t,0}(\xi) \\ &= \exp\biggl(\bigl(\widetilde{a}^+(\xi) - m\bigr)\Delta t - \bigl(\widetilde{a}^-(\xi) + A^-\bigr)\int_0^{\Delta t} q_{t+\tau} d\tau\biggr)\bigl(\widetilde{g}_t(\xi) + q_t\bigr). \end{aligned} \tag{A.1}$$

By the flow property of solutions to (3.3), $q_{t+\tau} = \widehat{q}_\tau$, where

$$\frac{d}{d\tau}\widehat{q}_\tau = A^+\widehat{q}_\tau - m\widehat{q}_\tau - A^-\widehat{q}_\tau^2$$

with the initial condition

$$\widehat{q}_\tau\Big|_{\tau=0} = q_t.$$

We have then

$$\frac{d}{d\tau}\log\widehat{q}_\tau = \frac{1}{\widehat{q}_\tau}\frac{d}{d\tau}\widehat{q}_\tau = A^+ - m - A^-\widehat{q}_\tau,$$

and integrating from $\tau = 0$ to $\tau = \vartriangle t$, we get

$$\log\widehat{q}_{\vartriangle t} - \log\widehat{q}_0 = \int_0^{\Delta t}\frac{d}{d\tau}\log\widehat{q}_\tau d\tau = (A^+ - m)\Delta t - A^-\int_0^{\Delta t}\widehat{q}_\tau d\tau.$$

Replacing back $\widehat{q}_\tau$ by $q_{t+\tau}$, we obtain

$$\int_0^{\Delta t} q_{t+\tau} d\tau = q_*\Delta t - \frac{1}{A^-}\log\frac{q_{t+\vartriangle t}}{q_t}. \tag{A.2}$$

Substituting into (A.1) and using that $m + q_*A^- = A^+$, one gets

$$h_{t,\vartriangle t}(\xi) = \exp\biggl(\bigl(\widetilde{J}_*(\xi) - A^+\bigr)\Delta t + \frac{\widetilde{a}^-(\xi) + A^-}{A^-}\log\frac{q_{t+\vartriangle t}}{q_t}\biggr)\bigl(\widetilde{g}_t(\xi) + q_t\bigr). \tag{A.3}$$

Then, one can rewrite:

$$h_{t,\vartriangle t}(\xi) = e^{(\widetilde{J}_*(\xi) - A^+)\vartriangle t}\biggl(\frac{q_{t+\vartriangle t}}{q_t}\biggr)^{\frac{\widetilde{a}^-(\xi)+A^-}{A^-}}\bigl(\widetilde{g}_t(\xi) + q_t\bigr).$$

To find $\widetilde{g}_{t,\vartriangle t}(\xi)$ from (3.21), one has to solve (3.25). By (2.35), $q^O_{\vartriangle t} = q_t$. Therefore, by (A.2),

$$\begin{aligned} q^O_{\vartriangle t} &= \exp\biggl(-m\Delta t - A^-\int_0^{\Delta t} q_{t+\tau}\, d\tau\biggr) q^O_0 \\ &= \exp\biggl(-A^+\Delta t + \log\Bigl(\frac{q_{t+\vartriangle t}}{q_t}\Bigr)\biggr) q_t = e^{-A^+\vartriangle t} q_{t+\vartriangle t}. \end{aligned} \tag{A.4}$$

As a result, (3.21) implies (3.26).

Step 2. Let now (3.10) and (**SL₂**) hold. Note that, by (3.3), (3.11).

$$\frac{d}{dt}q_t = A^- q_t(q_* - q_t) \geq 0,$$

hence q_t is increasing in $t \geq 0$, and, therefore,

$$\frac{q_{t+\Delta t}}{q_t} > 1, \qquad \Delta t > 0,\ t \geq 0. \tag{A.5}$$

Note also that, by (3.16),

$$|\widetilde{a}^-(\xi)| \leq A^-, \qquad \xi \in \mathbb{R}^d, \tag{A.6}$$

By (A.3), (A.4), (3.21), we have

$$\bigl|\widetilde{g}_{t,\Delta t}(\xi)\bigr| \leq E_1(t,\Delta t,\xi)\bigl|\widetilde{g}_t(\xi)\bigr| + \bigl|E_2(t,\Delta t,\xi)\bigr| q_t, \tag{A.7}$$

where

$$\begin{aligned} E_1(t,\Delta t,\xi) &= e^{(\widetilde{J}_*(\xi)-A^+)\Delta t}\Bigl(\frac{q_{t+\Delta t}}{q_t}\Bigr)^{\frac{\widetilde{a}^-(\xi)+A^-}{A^-}}, \\ E_2(t,\Delta t,\xi) &= E_1(t,\Delta t,\xi) - e^{-A^+\Delta t}\,\frac{q_{t+\Delta t}}{q_t}. \end{aligned}$$

By (3.11), ($\mathbf{SL_2}$), (A.5), (A.6),

$$E_1(t,\Delta t,\xi) \leq e^{-\alpha\Delta t}\Bigl(\frac{q_*}{q_0}\Bigr)^2.$$

To estimate $E_2(t,\Delta t,\xi)$, we can use an elementary inequality which holds for any constants $a,b,p,q \geq 0$:

$$\bigl|pe^{-a} - qe^{-b}\bigr| \leq e^{-a}|p-q| + q\max\bigl\{e^{-a},e^{-b}\bigr\}|a-b|;$$

note that here we take $a = A^+ - \widetilde{J}_*(\xi) \geq \alpha > 0$, because of ($\mathbf{SL_2}$). One gets then

$$\begin{aligned} \bigl|E_2(t,\Delta t,\xi)\bigr| &\leq e^{(\widetilde{J}_*(\xi)-A^+)\Delta t}\left|\Bigl(\frac{q_{t+\Delta t}}{q_t}\Bigr)^{\frac{\widetilde{a}^-(\xi)+A^-}{A^-}} - \frac{q_{t+\Delta t}}{q_t}\right| \\ &\quad + \frac{q_{t+\Delta t}}{q_t}\max\Bigl\{e^{(\widetilde{J}_*(\xi)-A^+)\Delta t}, e^{-A^+\Delta t}\Bigr\}\bigl|\widetilde{J}_*(\xi)\Delta t\bigr| \\ &\leq \frac{q_*}{q_t}e^{-\alpha\Delta t}\left|\Bigl(\frac{q_{t+\Delta t}}{q_t}\Bigr)^{\frac{\widetilde{a}^-(\xi)}{A^-}} - 1\right| + \frac{q_*}{q_t}\Delta t\, e^{-\min\{A^+,\alpha\}\Delta t}\bigl|\widetilde{J}_*(\xi)\bigr|. \end{aligned}$$

Next, for any $a \geq 1$ and $|x| \leq b$, we have

$$\begin{aligned} |a^x - 1| &= |e^{x\ln a} - 1| \leq \sum_{n=1}^\infty \frac{1}{n!}|x|^n(\ln a)^n \\ &\leq |x|\sum_{n=1}^\infty \frac{1}{n!}b^{n-1}(\ln a)^n \leq \frac{1}{b}|x|e^{b\ln a} = \frac{a^b}{b}|x|. \end{aligned} \tag{A.8}$$

Therefore, by (A.6), (3.11),

$$\left|\left(\frac{q_{t+\Delta t}}{q_t}\right)^{\frac{\widetilde{a}^-(\xi)}{A^-}} - 1\right| \le \frac{q_*}{q_t}\frac{|\widetilde{a}^-(\xi)|}{A^-}. \tag{A.9}$$

Substituting the obtained estimates into (A.7), we get

$$\begin{aligned}\bigl|\widetilde{g}_{t,\Delta t}(\xi)\bigr| &\le e^{-\alpha\Delta t}\Bigl(\frac{q_*}{q_0}\Bigr)^2\bigl|\widetilde{g}_t(\xi)\bigr| \\ &\quad + \frac{q_*^2}{q_0}e^{-\alpha\Delta t}\frac{|\widetilde{a}^-(\xi)|}{A^-} + q_*\Delta t\, e^{-\min\{A^+,\alpha\}\Delta t}\bigl|\widetilde{J}_*(\xi)\bigr|. \end{aligned}\tag{A.10}$$

Therefore, for each $\xi \in \mathbb{R}^d$ and $t \ge 0$,

$$\lim_{\Delta t\to\infty}\widetilde{g}_{t,\Delta t}(\xi) = 0.$$

Next, (A.10) implies that $\widetilde{g}_{t,\Delta t}(\xi)$ is integrable, and hence one can apply the inverse Fourier transform which will be then equal to $g_{t,\Delta t}(x)$ for a.a. $x \in \mathbb{R}^d$. Moreover, for each $t \ge 0$ and for a.a. $x \in \mathbb{R}^d$, we get, by (3.16), (A.10),

$$|g_{t,\Delta t}(x)| \le \int_{\mathbb{R}^d}\widetilde{g}_{t,\Delta t}(\xi)\,d\xi \to 0, \qquad \Delta t \to \infty.$$

Step 3. By (3.9), for any $t \ge 0$

$$\lim_{\Delta t\to\infty}\left(\frac{q_{t+\Delta t}}{q_t}\right)^{\frac{\widetilde{a}^-(\xi)+A^-}{A^-}} = \left(\frac{q_*}{q_t}\right)^{\frac{\widetilde{a}^-(\xi)+A^-}{A^-}} \le \left(\frac{q_*}{q_0}\right)^2;$$

and hence, by ($\mathbf{SL_2}$), we get that (3.26) implies (3.27).

Therefore, by (3.26), for any $\Delta t \ge 0$

$$\widetilde{g}_{\infty,\Delta t}(\xi) = \lim_{t\to\infty}\widetilde{g}_{t,\Delta t}(\xi) = e^{(\widetilde{J}_*(\xi)-A^+)\Delta t}\bigl(\widetilde{g}_*(\xi) + q_*\bigr) - e^{-A^+\Delta t}q_*,$$

where we used (3.18). From this, by (3.18), one gets (3.28). Note also that, by (3.12), (3.8),

$$h_*(\xi) = \lim_{t\to\infty}h_t(\xi) = \frac{q_*A^+}{A^+ - \widetilde{J}_*(\xi)}, \qquad \xi \in \mathbb{R}^d.$$

Next, by ($\mathbf{SL_2}$) and (A.6),

$$\begin{aligned}&\left|\frac{A^+q_*}{A^+ - \widetilde{J}_*(\xi)}e^{(\widetilde{J}_*(\xi)-A^+)\Delta t} - q_*e^{-A^+\Delta t}\right| \le q_*\left|\frac{A^+}{A^+ - \widetilde{J}_*(\xi)}e^{\widetilde{J}_*(\xi)\Delta t} - 1\right| \\ &\le q_*\left|\frac{A^+}{A^+ - \widetilde{J}_*(\xi)} - 1\right| + q_*\bigl|e^{\widetilde{J}_*(\xi)\Delta t} - 1\bigr| \le q_*\frac{|\widetilde{J}_*(\xi)|}{\alpha} + q_*\bigl|e^{\widetilde{J}_*(\xi)\Delta t} - 1\bigr|,\end{aligned}$$

and hence, by (A.8), $\widetilde{g}_{\infty,\Delta t}(\xi)$ is integrable.

Step 4. We have

$$
\begin{aligned}
&\bigl|\widetilde{g}_{t,\Delta t}(\xi)-\widetilde{g}_{\infty,\Delta t}(\xi)\bigr|\\
&\leq e^{(\widetilde{J}_*(\xi)-A^+)\Delta t}\Bigl(\frac{q_{t+\Delta t}}{q_t}\Bigr)^{\frac{\widetilde{a}^-(\xi)+A^-}{A^-}}\Bigl|\widetilde{g}_t(\xi)-\widetilde{g}_*(\xi)\Bigr|\\
&\quad+e^{(\widetilde{J}_*(\xi)-A^+)\Delta t}\left|\Bigl(\frac{q_{t+\Delta t}}{q_t}\Bigr)^{\frac{\widetilde{a}^-(\xi)+A^-}{A^-}}-1\right|\bigl|\widetilde{g}_*(\xi)\bigr|\\
&\quad+e^{-A^+\Delta t}\left|\biggl(e^{\widetilde{J}_*(\xi)\Delta t}\Bigl(\frac{q_{t+\Delta t}}{q_t}\Bigr)^{\frac{\widetilde{a}^-(\xi)}{A^-}}-1\biggr)q_{t+\Delta t}-e^{\widetilde{J}_*(\xi)\Delta t}q_*+q_*\right|\\
&\leq e^{-\alpha\Delta t}\Bigl(\frac{q_*}{q_0}\Bigr)^2\Bigl|\widetilde{g}_t(\xi)-\widetilde{g}_*(\xi)\Bigr|+e^{-\alpha\Delta t}\left|\Bigl(\frac{q_{t+\Delta t}}{q_t}\Bigr)^{\frac{\widetilde{a}^-(\xi)+A^-}{A^-}}-1\right|\bigl|\widetilde{g}_*(\xi)\bigr|\\
&\quad+q_*e^{-A^+\Delta t}e^{\widetilde{J}_*(\xi)\Delta t}\left|\Bigl(\frac{q_{t+\Delta t}}{q_t}\Bigr)^{\frac{\widetilde{a}^-(\xi)}{A^-}}-1\right|\\
&\quad+e^{-A^+\Delta t}\left|e^{\widetilde{J}_*(\xi)\Delta t}\Bigl(\frac{q_{t+\Delta t}}{q_t}\Bigr)^{\frac{\widetilde{a}^-(\xi)}{A^-}}-1\right|\bigl|q_{t+\Delta t}-q_*\bigr|\\
&=: I_1(\xi,t,\Delta t)+I_2(\xi,t,\Delta t)+I_3(\xi,t,\Delta t)+I_4(\xi,t,\Delta t).
\end{aligned}
$$

By [3, Theorem 3.2, Remark 3.3],

$$
\lim_{t\to\infty}\int_{\mathbb{R}^d} I_1(\xi,t,\Delta t)\,d\xi=0. \tag{A.11}
$$

By (3.9), we have, for all $\xi\in\mathbb{R}^d$, $\Delta t\geq 0$,

$$
\lim_{t\to\infty} I_2(\xi,t,\Delta t)=\lim_{t\to\infty} I_3(\xi,t,\Delta t)=\lim_{t\to\infty} I_4(\xi,t,\Delta t)=0.
$$

Next, we have

$$
I_2(\xi,t,\Delta t)\leq e^{-\alpha\Delta t}\biggl(\Bigl(\frac{q_*}{q_0}\Bigr)^2+1\biggr)\bigl|\widetilde{g}_*(\xi)\bigr|\in L^1(\mathbb{R}^d),\qquad t\geq 0;
$$

and by (A.9), (A.5),

$$
I_3(\xi,t,\Delta t)\leq q_*e^{-\alpha\Delta t}\cdot\frac{q_*}{q_0}\frac{|\widetilde{a}^-(\xi)|}{A^-}\in L^1(\mathbb{R}^d).
$$

Finally, rewriting back

$$
e^{\widetilde{J}_*(\xi)\Delta t}\Bigl(\frac{q_{t+\Delta t}}{q_t}\Bigr)^{\frac{\widetilde{a}^-(\xi)}{A^-}}=\exp\biggl(\widetilde{J}_*(\xi)\Delta t+\frac{\widetilde{a}^-(\xi)}{A^-}\ln\frac{q_{t+\Delta t}}{q_t}\biggr)
$$

and using that

$$
\left|\widetilde{J}_*(\xi)\Delta t+\frac{\widetilde{a}^-(\xi)}{A^-}\ln\frac{q_{t+\Delta t}}{q_t}\right|\leq(A^+-\alpha)\Delta t+\ln\frac{q^*}{q_0},
$$

we obtain from (A.8), that

$$I_4(\xi,t,\Delta t) \le 2q_* e^{-A^+\Delta t} \frac{\frac{q^*}{q_0} e^{(A^+-\alpha)\Delta t}}{(A^+-\alpha)\Delta t + \ln\frac{q^*}{q_0}} \biggl(|\widetilde{J}_*(\xi)|\Delta t + \frac{|\widetilde{a}^-(\xi)|}{A^-}\frac{q_*}{q_0} \biggr) \in L^1(\mathbb{R}^d).$$

Hence, by the dominated convergence theorem, (A.11) holds with I_1 replaced by each of I_2, I_3, I_4.

Therefore, $\widetilde{g}_{t,\Delta t}$ converges to $\widetilde{g}_{\infty,\Delta t}$ in $L^1(\mathbb{R}^d)$ as $t \to \infty$. Since $g_{t,\Delta t} - g_{\infty,\Delta t}$ is the inverse Fourier transform of $\widetilde{g}_{t,\Delta t} - \widetilde{g}_{\infty,\Delta t}$, we obtain from an analogue of (3.16) that $g_{t,\Delta t}$ converges to $g_{\infty,\Delta t}$ in $L^\infty(\mathbb{R}^d)$. □

Proof of Lemma 3.3

Firstly, for the Fourier transform defined as in (1.29), formula (3.31) follows from e.g. [4, Example 2.2.9, Proposition 2.2.11]. Next, we note that the Fourier transform of a (radially) symmetric function is also (radially) symmetric, and it coincides with the inverse Fourier transform of that function. Therefore, by e.g. [4, Back Matters B.5],

$$g(x) = 2\pi \int_0^\infty f\bigl(e^{-2\pi^2 r^2}\bigr) r \, \mathsf{J}_0\bigl(2\pi r|x|\bigr)\, dr, \qquad x \in \mathbb{R}^2.$$

Making the substitution $s = 2\pi r$, one gets the desired formula (3.32). □

Proof of Proposition 4.1

We set $f(x,y) = x(A^+ - A^-x - By)$, $g(x,y) = y(Bx - m)$. Consider the Jacobian

$$j(x,y) = \begin{pmatrix} f'_x(x,y) & f'_y(x,y) \\ g'_x(x,y) & g'_y(x,y) \end{pmatrix} = \begin{pmatrix} A^+ - 2A^-x - By & -Bx \\ By & Bx - m \end{pmatrix}.$$

Then, it is straightforward to check that

$$\mathsf{J} := j(q_*^H, q_*^P) = \begin{pmatrix} -\dfrac{A^-m}{B} & -m \\ A^+ - \dfrac{A^-m}{B} & 0 \end{pmatrix}.$$

Since

$$\mathrm{tr}(\mathsf{J}) = -\frac{A^-m}{B} < 0,$$
$$\det(\mathsf{J}) = m\biggl(A^+ - \frac{A^-m}{B}\biggr) > 0,$$

under $(\mathbf{HP_1})$, we conclude that both eigenvalues of $j(q_*^H, q_*^P)$ have negative real parts, hence (4.12) holds.

The monotone convergence in (4.12) takes place when the eigenvalues of J are real (and hence negative), otherwise there will be damping oscillations. The eigenvalues of J are real iff

$$\bigl(\mathrm{tr}(\mathsf{J})\bigr)^2 \ge 4\det(\mathsf{J}),$$

that is equivalent to

$$A^+ \le \frac{m}{B}A^- + \frac{m(A^-)^2}{4B^2},$$

and hence the statement is proved. $\square$

Proof of Proposition 4.2

We have, by (4.14)

$$\int_{\mathbb{R}^d} J_*^H(x)\,dx = A^+ - \frac{m}{B}A^-,$$

and hence (4.15) implies ($\mathbf{HP_1}$). Next, by (3.16), one has

$$\widetilde{J}_*^H(\xi) \le A^+ - \frac{m}{B}A^- < A^+, \qquad \xi\in\mathbb{R}^d, \tag{A.12}$$

$$\widetilde{b}(\xi) \le B, \qquad \xi\in\mathbb{R}^d. \tag{A.13}$$

Therefore,

$$A^+ - \widetilde{J}_*^H(\xi) \ge A^+ - \Bigl(A^+ - \frac{m}{B}A^-\Bigr) = \frac{m}{B}A^-, \qquad \xi\in\mathbb{R}^d,$$

hence ($\mathbf{HP_2}$) holds. Then,

$$\begin{aligned} r(\xi) :&= \bigl(A^+ - \widetilde{J}_*^H(\xi)\bigr)\bigl(B-\widetilde{b}(\xi)\bigr) + Bq_*^P\widetilde{b}(\xi) \\ &\ge \frac{m}{B}A^-\bigl(B-\widetilde{b}(\xi)\bigr) + Bq_*^P\widetilde{b}(\xi) = mA^- + \Bigl(A^+ - 2\frac{m}{B}A^-\Bigr)\widetilde{b}(\xi). \end{aligned} \tag{A.14}$$

If $A^+ \ge 2\frac{m}{B}A^-$, then, by (4.16), $r(\xi)\ge mA^-$. If, cf. ($\mathbf{HP_1}$),

$$\frac{m}{B}A^- < A^+ < 2\frac{m}{B}A^-,$$

then by (4.16) and (A.13), we have

$$\begin{aligned} r(\xi) &\ge mA^- - \Bigl(2\frac{m}{B}A^- - A^+\Bigr)\widetilde{b}(\xi) \ge mA^- - \Bigl(2\frac{m}{B}A^- - A^+\Bigr)B \\ &= A^+B - mA^- > 0, \end{aligned}$$

by ($\mathbf{HP_1}$). As a result,

$$\bigl(A^+ - \widetilde{J}_*^H(\xi)\bigr)\bigl(B-\widetilde{b}(\xi)\bigr) + Bq_*^P\widetilde{b}(\xi) \ge \max\bigl\{mA^-, A^+B - mA^-\bigr\} \tag{A.15}$$

for all $\xi\in\mathbb{R}^d$. $\square$

Possible relaxation of assumption (4.16)

Let (4.15) hold. One can weaken the assumption (4.16) as follows.

Let $\xi \in \mathbb{R}^d$ be such that $\widetilde{b}(\xi) < 0$ (provided that such ξ exists). If, additionally, $\widetilde{J}^H_*(\xi) \le \frac{m}{B} A^-$, then, cf. (A.14),

$$r(\xi) \ge \Bigl(A^+ - \frac{m}{B} A^-\Bigr)\bigl(B - 2\widetilde{b}(\xi)\bigr) > 0,$$

as $\widetilde{b}(\xi) < 0$.

Let now $\xi \in \mathbb{R}^d$ be such that $\widetilde{b}(\xi) < 0$ and $\widetilde{J}^H_*(\xi) > \frac{m}{B} A^-$. By (A.12), we have that then, with necessity, $A^+ > 2\frac{m}{B} A^-$. Then, by (A.12) and (A.13), one has:

$$\begin{aligned} r(\xi) &\ge \Bigl(\frac{m}{B} A^- \bigl(B - \widetilde{b}(\xi)\bigr) + \Bigl(A^+ - \frac{m}{B} A^-\Bigr)\widetilde{b}(\xi)\Bigr) \\ &= \Bigl(mA^- + \Bigl(A^+ - 2\frac{m}{B} A^-\Bigr)\widetilde{b}(\xi)\Bigr) > 0 \end{aligned}$$

if only

$$\Bigl(A^+ - 2\frac{m}{B} A^-\Bigr)|\widetilde{b}(\xi)| \le \Bigl(A^+ - 2\frac{m}{B} A^-\Bigr)B < mA^-,$$

i.e. if $A^+ < 3\frac{m}{B} A^-$.

Therefore, assuming (4.15), one can replace (4.16) by the following assumption:

- let either

$$A^+ < 3\frac{A^- m}{B}$$

- or

$$A^+ \ge 3\frac{A^- m}{B}, \tag{A.16}$$

$$\widetilde{b}(\xi) \ge 0, \qquad \xi \in \Lambda := \Bigl\{\xi \in \mathbb{R}^d : \widetilde{J}^H_*(\xi) \ge \frac{A^- m}{B}\Bigr\}. \tag{A.17}$$

Note that since $\widetilde{J}^H_*(0) = A^+ - \frac{A^- m}{B} > \frac{A^- m}{B}$ under (A.16), the set Λ is not empty, it contains a neighborhood of the origin. □

Remark A.1. A natural example when (A.17) may fail is

$$b(x) = C(1 + |x|^2)e^{-s|x|^2}, \qquad s > 0, \quad C > 0, \quad x \in \mathbb{R}^d, \tag{A.18}$$

with small enough $s > 0$. Indeed, it is straightforward to check that $\widetilde{b}(\xi) < 0$ for $|\xi| > d(s)$ for certain continuous $d(s)$ with $d(0) = 0$. Therefore, taking $s > 0$ small enough we ensure that $\widetilde{b}(\xi) < 0$ for some $\xi \in \Lambda$ given by (A.17). Note also that a small s here corresponds to a large length scale of the kernel $b(x)$ describing the influence of parasites on hosts.

Proof of Theorem 4.4

Denoting, cf. (4.9),

$$\overline{\overline{g}}_t(\xi) := \Bigl(\widetilde{g}_t^{HH}(\xi), \widetilde{g}_t^{HP}(\xi), \widetilde{g}_t^{PH}(\xi), \widetilde{g}_t^{PP}(\xi)\Bigr)^T,$$

we get

$$\begin{aligned}
\frac{d}{dt}\overline{\overline{g}}_t(\xi) &= \frac{d}{dt}\overline{h}_t(\xi) - \frac{d}{dt}(q_t^H, 0, 0, q_t^P)^T \\
&= \Bigl(\mathcal{A}_t(\xi) + \mathcal{A}_t'(\xi)\Bigr)\overline{h}_t(\xi) + \mathcal{B}_t(\xi) - \frac{d}{dt}(q_t^H, 0, 0, q_t^P)^T \\
&= \Bigl(\mathcal{A}_t(\xi) + \mathcal{A}_t'(\xi)\Bigr)\overline{\overline{g}}_t(\xi) + f_t(\xi),
\end{aligned}$$

where

$$\begin{aligned}
f_t(\xi) :&= \mathcal{B}_t(\xi) + \Bigl(\mathcal{A}_t(\xi) + \mathcal{A}_t'(\xi)\Bigr)(q_t^H, 0, 0, q_t^P)^T - \frac{d}{dt}(q_t^H, 0, 0, q_t^P)^T \\
&= \mathcal{B}_t(\xi) + \Bigl(\mathcal{A}_t(\xi) + \mathcal{A}_t'(\xi)\Bigr)(q_t^H, 0, 0, q_t^P)^T \\
&\quad - \bigl(q_t^H(A^+ - A^- q_t^H - Bq_t^P), 0, 0, q_t^P(Bq_t^H - m)\bigr)^T.
\end{aligned}$$

Recall that $\widetilde{g}_t^{HP}(\xi) = \widetilde{g}_t^{HP}(\xi)$. We can hence rewrite this in terms of the vector

$$\overline{\mathtt{g}}_t(\xi) := (\widetilde{g}_t^{HH}(\xi), \widetilde{g}_t^{HP}(\xi), \widetilde{g}_t^{PP}(\xi))^T.$$

It is easy to see that, if the remove the third component (equal to the second one) of the vector $\Bigl(\mathcal{A}_t(\xi) + \mathcal{A}_t'(\xi)\Bigr)\overline{\overline{g}}_t(\xi)$, we will get the vector $\mathcal{C}_t(\xi)\overline{\mathtt{g}}_t(\xi)$, where

$$\mathcal{C}_t(\xi) := \begin{pmatrix} 2\mathtt{a}_t(\xi) & 2\mathtt{b}_t(\xi) & 0 \\ \mathtt{c}_t & \mathtt{a}_t(\xi) + \mathtt{d}_t(\xi) & \mathtt{b}_t(\xi) \\ 0 & 2\mathtt{c}_t & 2\mathtt{d}_t(\xi). \end{pmatrix}$$

We consider also

$$\mathtt{B}_t(\xi) := \Bigl(A^+ q_t^H + A^- \bigl(q_t^H\bigr)^2, 0, m q_t^P\Bigr)^T + Bq_t^H q_t^P \Bigl(1, -1, 1\Bigr)^T$$

Then

$$\begin{aligned}
\frac{d}{dt}\overline{\mathtt{g}}_t(\xi) &= \mathcal{C}_t(\xi)\overline{\mathtt{g}}_t(\xi) + \mathtt{f}_t(\xi), \\
\mathtt{f}_t(\xi) :&= \mathtt{B}_t(\xi) + \mathcal{C}_t(\xi)(q_t^H, 0, q_t^P)^T - \frac{d}{dt}(q_t^H, 0, q_t^P)^T \\
&= \mathtt{B}_t(\xi) + \mathcal{C}_t(\xi)(q_t^H, 0, q_t^P)^T - \bigl(q_t^H(A^+ - A^- q_t^H - Bq_t^P), 0, q_t^P(Bq_t^H - m)\bigr)^T.
\end{aligned}$$

It is straightforward to check that we can apply now [3, Lemma 3.1] in both Banach spaces $X = \bigl(L^1(\mathbb{R}^d)\bigr)^{\otimes 3}$ or $X = \bigl(L^\infty(\mathbb{R}^d)\bigr)^{\otimes 3}$, provided that there exist limits $\mathtt{f}_* := \lim\limits_{t\to\infty} \mathtt{f}_t \in X$ and $\mathcal{C}_* := \lim\limits_{t\to\infty} \mathcal{C}_t \in \mathcal{L}(X)$ (the space of bounded linear

operators on X) and also provided that the operator (matrix) $\mathcal{C}_*$ is invertible. We have that $\mathcal{C}_*(\xi)$ is given by (4.18) and

$$\begin{aligned}
\mathtt{f}_*(\xi) := {} & \Bigl(A^+ q_*^H + A^- \bigl(q_*^H\bigr)^2, 0, m q_*^P\Bigr)^T + B q_*^H q_*^P \Bigl(1, -1, 1\Bigr)^T \\
& + \mathcal{C}_*(\xi)(q_*^H, 0, q_*^P)^T + (0,0,0)^T \\
= {} & \Bigl(q_*^H (A^+ + A^- q_*^H + B q_*^P), -B q_*^H q_*^P, q_*^P (m + B q_*^H)\Bigr)^T + \mathcal{C}_*(\xi)(q_*^H, 0, q_*^P)^T \\
= {} & \Bigl(2A^+ q_*^H, -B q_*^H q_*^P, 2m q_*^P\Bigr)^T + \begin{pmatrix} 2\mathtt{a}_*(\xi) & 2\mathtt{b}_*(\xi) & 0 \\ \mathtt{c}_* & \mathtt{a}_*(\xi) + \mathtt{d}_*(\xi) & \mathtt{b}_*(\xi) \\ 0 & 2\mathtt{c}_* & 2\mathtt{d}_*(\xi) \end{pmatrix} (q_*^H, 0, q_*^P)^T \\
= {} & \Bigl(2 q_*^H \widetilde{J}_*^H(\xi), -q_*^H q_*^P \widetilde{b}(\xi), 2 q_*^H q_*^P \widetilde{b}(\xi)\Bigr)^T.
\end{aligned}$$

Th show that $\mathcal{C}_*$ is invertible in X it is evidently enough to show that the function $\det\bigl(\mathcal{C}_*(\xi)\bigr)$ is separated from 0. We have

$$\begin{aligned}
\det\bigl(\mathcal{C}_*(\xi)\bigr) &= 4\mathtt{a}_*(\xi)\mathtt{d}_*(\xi)\bigl(\mathtt{a}_*(\xi) + \mathtt{d}_*(\xi)\bigr) - 4\mathtt{a}_*(\xi)\mathtt{b}_*(\xi)\mathtt{c}_* - 4\mathtt{b}_*(\xi)\mathtt{c}_*\mathtt{d}_*(\xi) \\
&= 4\bigl(\mathtt{a}_*(\xi) + \mathtt{d}_*(\xi)\bigr)\bigl(\mathtt{a}_*(\xi)\mathtt{d}_*(\xi) - \mathtt{b}_*(\xi)\mathtt{c}_*\bigr).
\end{aligned}$$

By ($\mathbf{HP_2}$) and (A.13),

$$\mathtt{a}_*(\xi) + \mathtt{d}_*(\xi) = \widetilde{J}_*^H(\xi) - A^+ + q_*^H\bigl(\widetilde{b}(\xi) - B\bigr) \le -\alpha < 0. \tag{A.19}$$

By (A.15),

$$\begin{aligned}
\mathtt{a}_*(\xi)\mathtt{d}_*(\xi) - \mathtt{b}_*(\xi)\mathtt{c}_* &= q_*^H \bigl(\widetilde{J}_*^H(\xi) - A^+\bigr)\bigl(\widetilde{b}(\xi) - B)\bigr) + B q_*^P q_*^H \widetilde{b}(\xi) \\
&= q_*^H \Bigl(\bigl(A^+ - \widetilde{J}_*^H(\xi)\bigr)\bigl(B - \widetilde{b}(\xi))\bigr) + B q_*^P \widetilde{b}(\xi)\Bigr) \\
&\ge \frac{m}{B}\max\bigl\{mA^-, A^+B - mA^-\bigr\} > 0.
\end{aligned} \tag{A.20}$$

Therefore,

$$\det\bigl(\mathcal{C}_*(\xi)\bigr) \le -4\frac{m^2}{B^2} A^- \max\bigl\{mA^-, A^+B - mA^-\bigr\} < 0, \tag{A.21}$$

the proves the needed.

As a result, by [3, Lemma 3.1],

$$\begin{aligned}
\Bigl(\widetilde{g}_*^{HH}(\xi), \widetilde{g}_*^{HP}(\xi), \widetilde{g}_*^{PP}(\xi)\Bigr)^T &= -\Bigl(\mathcal{C}_*(\xi)\Bigr)^{-1} \mathtt{f}_*(\xi) \\
&= q_*^H \Bigl(\mathcal{C}_*(\xi)\Bigr)^{-1} \Bigl(-2\widetilde{J}_*^H(\xi), q_*^P \widetilde{b}(\xi), -2 q_*^P \widetilde{b}(\xi)\Bigr)^T,
\end{aligned}$$

and moreover $\widetilde{g}_t^{AB} \to \widetilde{g}_*^{AB}$ in both $L^1(\mathbb{R}^d)$ and $L^\infty(X)$ as $t \to \infty$, $A, B \in \{H, P\}$. The convergence in L^1 implies, by ($\mathbf{SL_2}$), the convergence in $L^\infty(\mathbb{R}^d)$ for the inverse Fourier transforms, that fulfills the proof. □

Proof of Corollary 4.5

Since $h_*^{HP}(\xi) = h_*^{PH}(\xi)$, (4.21) is equivalent to

$$\mathcal{C}_*(\xi)\Big(h_*^{HH}(\xi), h_*^{HP}(\xi), h_*^{PP}(\xi)\Big)^T = \big(-2A^+q_*^H, mq_*^P, -2mq_*^P\big)^T,$$

where $\mathcal{C}_*(\xi)$ is given by (4.18). By (A.21), we have then (4.22).

On the other hand,

$$\begin{aligned}
&\Big(g_*^{HH}(\xi), g_*^{HP}(\xi), g_*^{PP}(\xi)\Big)^T + (q_*^H, 0, q_*^P)^T \\
&= q_*^H\Big(\mathcal{C}_*(\xi)\Big)^{-1}\Big(-2\widetilde{J}_*^H(\xi), q_*^P\widetilde{b}(\xi), -2q_*^P\widetilde{b}(\xi)\Big)^T + (q_*^H, 0, q_*^P)^T \\
&= \Big(\mathcal{C}_*(\xi)\Big)^{-1}\Big(q_*^H\big(-2\widetilde{J}_*^H(\xi), q_*^P\widetilde{b}(\xi), -2q_*^P\widetilde{b}(\xi)\big)^T + \mathcal{C}_*(\xi)(q_*^H, 0, q_*^P)^T\Big) \\
&= \Big(\mathcal{C}_*(\xi)\Big)^{-1}\bigg(q_*^H\big(-2\widetilde{J}_*^H(\xi), q_*^P\widetilde{b}(\xi), -2q_*^P\widetilde{b}(\xi)\big)^T \\
&\qquad\qquad + \Big(2q_*^H(\widetilde{J}_*^H(\xi) - A^+), q_*^Hq_*^P(B - \widetilde{b}(\xi)), 2q_*^Hq_*^P(\widetilde{b}(\xi) - B)\Big)^T\bigg) \\
&= \Big(\mathcal{C}_*(\xi)\Big)^{-1}\big(-2A^+q_*^H, mq_*^P, -2mq_*^P\big)^T = \Big(h_*^{HH}(\xi), h_*^{HP}(\xi), h_*^{PP}(\xi)\Big)^T,
\end{aligned}$$

that fulfills the statement. □

Proof of Proposition 4.6

Step 1. We have, by (2.40),

$$h_{t,0}^{AB}(\xi) = h_t^{AB}(\xi), \qquad A, B \in \{H, P\},$$

where, recall, h_t^{AB} satisfy (4.9), (4.10). Then, by (4.31), (4.30), we obtain

$$\begin{pmatrix} h_{t,\Delta t}^{HH}(\xi) \\ h_{t,\Delta t}^{HP}(\xi) \end{pmatrix} = \exp\Big(\int_0^{\Delta t} \mathcal{E}_{t+\tau}(\xi)\, d\tau\Big)\begin{pmatrix} h_t^{HH}(\xi) \\ h_t^{HP}(\xi) \end{pmatrix}$$

and

$$\begin{pmatrix} h_{t,\Delta t}^{PH}(\xi) \\ h_{t,\Delta t}^{PP}(\xi) \end{pmatrix} = \exp\Big(\int_0^{\Delta t} \mathcal{E}_{t+\tau}(\xi)\, d\tau\Big)\begin{pmatrix} h_t^{HP}(\xi) \\ h_t^{PP}(\xi) \end{pmatrix},$$

where

$$\int_0^{\Delta t} \mathcal{E}_{t+\tau}(\xi)\, d\tau = \begin{pmatrix} \displaystyle\int_0^{\Delta t} \mathtt{a}_{t+\tau}(\xi)\, d\tau & \displaystyle\int_0^{\Delta t} \mathtt{b}_{t+\tau}(\xi)\, d\tau \\ \displaystyle\int_0^{\Delta t} \mathtt{c}_{t+\tau}\, d\tau & \displaystyle\int_0^{\Delta t} \mathtt{d}_{t+\tau}(\xi)\, d\tau \end{pmatrix}. \tag{A.22}$$

Similarly to the proof of Theorem 3.2, we note that $q^H_{t+\tau}$ and $q^P_{t+\tau}$ solves the system

$$\begin{aligned} \frac{d}{d\tau}\widehat{q}^H_\tau &= \widehat{q}^H_\tau\big(A^+ - A^-\widehat{q}^H_\tau - B\widehat{q}^P_\tau\big),\\ \frac{d}{d\tau}\widehat{q}^P_\tau &= \widehat{q}^P_\tau\big(B\widehat{q}^H_\tau - m\big) \end{aligned} \tag{A.23}$$

with the initial conditions $\widehat{q}^H_0 = q^H_t$, $\widehat{q}^P_0 = q^P_t$. Next, we rewrite (A.23) as follows

$$\begin{aligned} \frac{d}{d\tau}\log\widehat{q}^H_\tau &= A^+ - A^-\widehat{q}^H_\tau - B\widehat{q}^P_\tau,\\ \frac{d}{d\tau}\log\widehat{q}^P_\tau &= B\widehat{q}^H_\tau - m. \end{aligned}$$

Integrating over $\tau \in [0, \Delta t]$, we get

$$\begin{aligned} \log\frac{\widehat{q}^H_{\Delta t}}{\widehat{q}^H_0} &= A^+\Delta t - A^-\int_0^{\Delta t}\widehat{q}^H_\tau\,d\tau - B\int_0^{\Delta t}\widehat{q}^P_\tau\,d\tau,\\ \log\frac{\widehat{q}^P_{\Delta t}}{\widehat{q}^P_0} &= B\int_0^{\Delta t}\widehat{q}^H_\tau\,d\tau - m\Delta t. \end{aligned}$$

Therefore,

$$\begin{aligned} \int_0^{\Delta t}\widehat{q}^H_\tau\,d\tau &= q^H_*\Delta t + \frac{1}{B}\log\frac{\widehat{q}^P_{\Delta t}}{\widehat{q}^P_0},\\ \int_0^{\Delta t}\widehat{q}^P_\tau\,d\tau &= q^P_*\Delta t - \frac{1}{B}\log\frac{\widehat{q}^H_{\Delta t}}{\widehat{q}^H_0} - \frac{A^-}{B^2}\log\frac{\widehat{q}^P_{\Delta t}}{\widehat{q}^P_0}, \end{aligned}$$

hence

$$\begin{aligned} \int_0^{\Delta t} q^H_{t+\tau}\,d\tau &= q^H_*\Delta t + \frac{1}{B}\log\frac{q^P_{t+\Delta t}}{q^P_t},\\ \int_0^{\Delta t} q^P_{t+\tau}\,d\tau &= q^P_*\Delta t - \frac{1}{B}\log\frac{q^H_{t+\Delta t}}{q^H_t} - \frac{A^-}{B^2}\log\frac{q^P_{t+\Delta t}}{q^P_t}. \end{aligned} \tag{A.24}$$

Remark A.2. It should be mentioned that the trajectories of the system (4.3) cannot intersect, therefore, $q^H_t > 0$ and $q^P_t > 0$ for all $t > 0$, provided that it holds for $t = 0$.

By (A.24) and (4.4), we have

$$\begin{aligned} \int_0^{\Delta t}\mathsf{a}_{t+\tau}(\xi)\,d\tau &= \widetilde{a}^+(\xi)\Delta t - \big(A^- + \widetilde{a}^-(\xi)\big)\bigg(q^H_*\Delta t + \frac{1}{B}\log\frac{q^P_{t+\Delta t}}{q^P_t}\bigg)\\ &\quad - B\bigg(q^P_*\Delta t - \frac{1}{B}\log\frac{q^H_{t+\Delta t}}{q^H_t} - \frac{A^-}{B^2}\log\frac{q^P_{t+\Delta t}}{q^P_t}\bigg)\\ &= \big(\widetilde{J}^H_*(\xi) - A^+\big)\Delta t + \log\frac{q^H_{t+\Delta t}}{q^H_t} - \frac{\widetilde{a}^-(\xi)}{B}\log\frac{q^P_{t+\Delta t}}{q^P_t}, \end{aligned}$$

$$\int_0^{\Delta t} \mathsf{b}_{t+\tau}(\xi)\,d\tau = -\widetilde{b}(\xi)\biggl(q_*^H\Delta t + \frac{1}{B}\log\frac{q_{t+\Delta t}^P}{q_t^P}\biggr),$$

$$\int_0^{\Delta t} \mathsf{c}_{t+\tau}\,d\tau = B\biggl(q_*^P\Delta t - \frac{1}{B}\log\frac{q_{t+\Delta t}^H}{q_t^H} - \frac{A^-}{B^2}\log\frac{q_{t+\Delta t}^P}{q_t^P}\biggr),$$

$$\int_0^{\Delta t} \mathsf{d}_{t+\tau}(\xi)\,d\tau = \widetilde{b}(\xi)\biggl(q_*^H\Delta t + \frac{1}{B}\log\frac{q_{t+\Delta t}^P}{q_t^P}\biggr) - m\Delta t.$$

Therefore, by (A.22),

$$\int_0^{\Delta t} \mathcal{E}_{t+\tau}(\xi)\,d\tau = \Delta t\cdot\mathcal{E}_*(\xi) + \mathcal{F}_{t,\Delta t}(\xi),$$

where $\mathcal{E}_*(\xi)$ is given by (4.38), i.e., cf. (4.17),

$$\mathcal{E}_*(\xi) = \begin{pmatrix} \widetilde{J}_*^H(\xi) - A^+ & -q_*^H\widetilde{b}(\xi) \\ Bq_*^P & \widetilde{b}(\xi)q_*^H - m \end{pmatrix} \tag{A.25}$$

and

$$\mathcal{F}_{t,\Delta t}(\xi) = \log\frac{q_{t+\Delta t}^H}{q_t^H}\begin{pmatrix} 1 & 0 \\ -1 & 0 \end{pmatrix} - \frac{1}{B}\log\frac{q_{t+\Delta t}^P}{q_t^P}\begin{pmatrix} \widetilde{a}^-(\xi) & \widetilde{b}(\xi) \\ A^- & -\widetilde{b}(\xi) \end{pmatrix}. \tag{A.26}$$

Note that (4.38) holds.

As a result,

$$\begin{pmatrix} h_{t,\Delta t}^{HH}(\xi) \\ h_{t,\Delta t}^{HP}(\xi) \end{pmatrix} = \exp\Bigl(\Delta t\cdot\mathcal{E}_*(\xi) + \mathcal{F}_{t,\Delta t}(\xi)\Bigr)\begin{pmatrix} h_t^{HH}(\xi) \\ h_t^{HP}(\xi) \end{pmatrix}, \tag{A.27}$$

$$\begin{pmatrix} h_{t,\Delta t}^{PH}(\xi) \\ h_{t,\Delta t}^{PP}(\xi) \end{pmatrix} = \exp\Bigl(\Delta t\cdot\mathcal{E}_*(\xi) + \mathcal{F}_{t,\Delta t}(\xi)\Bigr)\begin{pmatrix} h_t^{HP}(\xi) \\ h_t^{PP}(\xi) \end{pmatrix}, \tag{A.28}$$

Step 2. Find limits of $\widetilde{g}_{t,\Delta t}^{AB}(\xi)$ as $|\xi|\to\infty$, $A,B\in\{H,P\}$. Let $\Delta t>0$ (otherwise, there is nothing to prove). By (4.25),

$$\widetilde{g}_{t,\Delta t}^{HH}(\xi) = h_{t,\Delta t}^{HH}(\xi) - q_{\Delta t}^{HO}, \qquad \widetilde{g}_{t,\Delta t}^{PP}(\xi) = h_{t,\Delta t}^{PP}(\xi) - q_{\Delta t}^{PO};$$

and by (4.32), for each $t\geq 0$, $\xi\in\mathbb{R}^d$,

$$q_{\Delta t}^{HO} = \exp\biggl(-\int_0^{\Delta t}\bigl(Bq_{t+\tau}^P + A^-q_{t+\tau}^H\bigr)\,d\tau\biggr)q_0^{HO}$$

and using (A.24), one can continue

$$\begin{aligned} &= \exp\biggl(-(Bq_*^P + A^-q_*^H)\Delta t + \log\frac{q_{t+\Delta t}^H}{q_t^H}\biggr)q_t^H \\ &= e^{-A^+\Delta t}\, q_{t+\Delta t}^H; \end{aligned} \tag{A.29}$$

and also by (4.32),

$$q^{PO}_{\Delta t} = e^{-m\Delta t} q^{PO}_0 = e^{-m\Delta t} q^P_t. \tag{A.30}$$

Denoting henceforth $f(\infty) = \lim_{|\xi|\to\infty} f(\xi)$ for a function (or a matrix-valued function) f and using the Riemann–Lebesgue lemma, we obtain

$$\mathtt{a}_*(\infty) = -A^+, \quad \mathtt{b}_*(\infty) = 0, \quad \mathtt{c}_* = Bq^P_*, \quad \mathtt{d}_*(\infty) = -Bq^H_* = -m,$$

and hence

$$\begin{aligned}
&\Delta t \cdot \mathcal{E}_*(\infty) + \mathcal{F}_{t,\Delta t}(\infty)\\
&= \Delta t \cdot \begin{pmatrix} -A^+ & 0 \\ Bq^P_* & -m \end{pmatrix} + \log \frac{q^H_{t+\Delta t}}{q^H_t} \begin{pmatrix} 1 & 0 \\ -1 & 0 \end{pmatrix} - \frac{1}{B} \log \frac{q^P_{t+\Delta t}}{q^P_t} \begin{pmatrix} 0 & 0 \\ A^- & 0 \end{pmatrix}\\
&= \begin{pmatrix} -A^+\Delta t + \log \frac{q^H_{t+\Delta t}}{q^H_t} & 0 \\ Bq^P_*\Delta t - \log \frac{q^H_{t+\Delta t}}{q^H_t} - \frac{1}{B}\log \frac{q^P_{t+\Delta t}}{q^P_t} & -m\Delta t \end{pmatrix}.
\end{aligned}$$

Next, any matrix

$$M = \begin{pmatrix} a & 0 \\ c & d \end{pmatrix}, \qquad a, c, d \in \mathbb{R},$$

has eigenvalues a and d and the corresponding eigenvectors $\left(\frac{a-d}{c}, 1\right)^T$ and $(0,1)^T$. Therefore,

$$\begin{aligned}
e^M &= \begin{pmatrix} \frac{a-d}{c} & 0 \\ 1 & 1 \end{pmatrix} \begin{pmatrix} e^a & 0 \\ 0 & e^d \end{pmatrix} \begin{pmatrix} \frac{a-d}{c} & 0 \\ 1 & 1 \end{pmatrix}^{-1} = \begin{pmatrix} \frac{a-d}{c} & 0 \\ 1 & 1 \end{pmatrix} \begin{pmatrix} e^a & 0 \\ 0 & e^d \end{pmatrix} \begin{pmatrix} \frac{c}{a-d} & 0 \\ -\frac{c}{a-d} & 1 \end{pmatrix}\\
&= \begin{pmatrix} e^a & 0 \\ \frac{c}{a-d}(e^a - e^d) & e^d \end{pmatrix}.
\end{aligned}$$

Therefore,

$$\exp\Big(\Delta t \cdot \mathcal{E}_*(\xi) + \mathcal{F}_{t,\Delta t}(\xi)\Big) = \begin{pmatrix} \frac{q^H_{t+\Delta t}}{q^H_t} e^{-A^+\Delta t} & 0 \\ v_{t,\Delta t} & e^{-m\Delta t}, \end{pmatrix}$$

where

$$v_{t,\Delta t} = \frac{Bq^P_*\Delta t - \log \frac{q^H_{t+\Delta t}}{q^H_t} - \frac{1}{B}\log\frac{q^P_{t+\Delta t}}{q^P_t}}{-(A^+ - m)\Delta t + \log \frac{q^H_{t+\Delta t}}{q^H_t}} \left(\frac{q^H_{t+\Delta t}}{q^H_t} e^{-A^+\Delta t} - e^{-m\Delta t} \right).$$

Next, by Theorem 4.4, Corollary 4.5,

$$h^{HH}_t(\infty) = q^H_t, \qquad h^{HP}_t(\infty) = h^{PH}_t(\infty) = 0, \qquad h^{PP}_t(\infty) = q^P_t.$$

Then, by (A.27), (A.28),

$$\begin{pmatrix} h^{HH}_{t,\Delta t}(\infty) \\ h^{HP}_{t,\Delta t}(\infty) \end{pmatrix} = \begin{pmatrix} \frac{q^H_{t+\Delta t}}{q^H_t}\, e^{-A^+\Delta t} & 0 \\ v_{t,\Delta t} & e^{-m\Delta t}, \end{pmatrix} \begin{pmatrix} q^H_t \\ 0 \end{pmatrix} = \begin{pmatrix} \frac{q^H_{t+\Delta t}}{q^H_t}\, e^{-A^+\Delta t} q^H_t \\ v_{t,\Delta t} q^H_t \end{pmatrix}, \tag{A.31}$$

$$\begin{pmatrix} h^{PH}_{t,\Delta t}(\infty) \\ h^{PP}_{t,\Delta t}(\infty) \end{pmatrix} = \begin{pmatrix} \frac{q^H_{t+\Delta t}}{q^H_t}\, e^{-A^+\Delta t} & 0 \\ v_{t,\Delta t} & e^{-m\Delta t}, \end{pmatrix} \begin{pmatrix} 0 \\ q^P_t \end{pmatrix} = \begin{pmatrix} 0 \\ e^{-m\Delta t} q^P_t \end{pmatrix}. \tag{A.32}$$

As a result,

$$\widetilde{g}^{PH}_{t,\Delta t}(\infty) = h^{PH}_{t,\Delta t}(\infty) = 0,$$

next, by (A.29), (A.30)

$$\begin{aligned} h^{HH}_{t,\Delta t}(\infty) &= q^{HO}_{\Delta t}, \quad \text{and hence } \widetilde{g}^{HH}_{t,\Delta t}(\infty) = 0, \\ h^{PP}_{t,\Delta t}(\infty) &= q^{PO}_{\Delta t}, \quad \text{and hence } \widetilde{g}^{PP}_{t,\Delta t}(\infty) = 0, \end{aligned}$$

whereas

$$\begin{aligned} \widetilde{g}^{HP}_{t,\Delta t}(\infty) &= h^{HP}_{t,\Delta t}(\infty) = v_{t,\Delta t} q^H_t \\ &= \frac{B q^P_* \Delta t - \log \frac{q^H_{t+\Delta t}}{q^H_t} - \frac{1}{B} \log \frac{q^P_{t+\Delta t}}{q^P_t}}{-(A^+ - m)\Delta t + \log \frac{q^H_{t+\Delta t}}{q^H_t}} \Bigl(q^H_{t+\Delta t}\, e^{-A^+\Delta t} - q^H_t\, e^{-m\Delta t} \Bigr), \end{aligned} \tag{A.33}$$

and hence, in general, $\widetilde{g}^{HP}_{t,\Delta t}(\infty) \neq 0$.

Step 3. Prove the integrability of $\widetilde{g}^{AB}_{t,\Delta t}(\xi)$, $AB \in \{HH, HP', PH, PP\}$. We denote

$$M_{t,\Delta t}(\xi) = \Delta t \cdot \mathcal{E}_*(\xi) + \mathcal{F}_{t,\Delta t}(\xi). \tag{A.34}$$

By the above,

$$\begin{aligned} \begin{pmatrix} \widetilde{g}^{HH}_{t,\Delta t}(\xi) \\ \widetilde{g}^{HP'}_{t,\Delta t}(\xi) \end{pmatrix} &= \begin{pmatrix} h^{HH}_{t,\Delta t}(\xi) \\ h^{HP}_{t,\Delta t}(\xi) \end{pmatrix} - \begin{pmatrix} h^{HH}_{t,\Delta t}(\infty) \\ h^{HP}_{t,\Delta t}(\infty) \end{pmatrix} \\ &= e^{M_{t,\Delta t}(\xi)} \begin{pmatrix} h^{HH}_t(\xi) \\ h^{HP}_t(\xi) \end{pmatrix} - e^{M_{t,\Delta t}(\infty)} \begin{pmatrix} q^H_t \\ 0 \end{pmatrix} \\ &= e^{M_{t,\Delta t}(\xi)} \begin{pmatrix} \widetilde{g}^{HH}_t(\xi) \\ \widetilde{g}^{HP}_t(\xi) \end{pmatrix} + \bigl(e^{M_{t,\Delta t}(\xi)} - e^{M_{t,\Delta t}(\infty)} \bigr) \begin{pmatrix} q^H_t \\ 0 \end{pmatrix}. \end{aligned} \tag{A.35}$$

The entries of matrix $M_{t,\Delta t}(\xi)$ are bounded in ξ functions, and also functions $\widetilde{g}^{HH}_t(\xi)$, $\widetilde{g}^{HP}_t(\xi)$ are integrable, by the proof of Theorem 4.4 above. Then the first summand in (A.35) is integrable. The second summand in (A.35) is a vector which has integrable in ξ entries iff its (any) norm is integrable. The latter evidently holds if $\bigl\| e^{M_{t,\Delta t}(\xi)} - e^{M_{t,\Delta t}(\infty)} \bigr\|$ is integrable.

For any (square) matrices A and B, one has, for $n \in \mathbb{N}$,

$$A^n - B^n = \sum_{j=0}^{n-1} A^j (A - B) B^{n-1-j},$$

hence

$$\|A^n - B^n\| \leq n \max\{\|A\|, \|B\|\}^{n-1} \|A - B\|.$$

Therefore,

$$\begin{aligned}\|e^A - e^B\| &\leq \sum_{n=1}^{\infty} \frac{1}{n!} \|A^n - B^n\| \leq \sum_{n=1}^{\infty} \frac{1}{(n-1)!} \max\{\|A\|, \|B\|\}^{n-1} \|A - B\| \\ &= e^{\max\{\|A\|, \|B\|\}} \|A - B\|. \end{aligned} \tag{A.36}$$

Hence, since

$$M_{t,\Delta t}(\xi) - M_{t,\Delta t}(\infty) = \Delta t \cdot \begin{pmatrix} \widetilde{J}^H_*(\xi) & -q^H_* \widetilde{b}(\xi) \\ 0 & q^H_* \widetilde{b}(\xi) \end{pmatrix} - \frac{1}{B} \log \frac{q^P_{t+\Delta t}}{q^P_t} \begin{pmatrix} \widetilde{a}^-(\xi) & \widetilde{b}(\xi) \\ 0 & -\widetilde{b}(\xi) \end{pmatrix},$$

one can conclude that $\left\| e^{M_{t,\Delta t}(\xi)} - e^{M_{t,\Delta t}(\infty)} \right\|$ is integrable, so such are the entries of the second summand in (A.35).

The proof of the integrability of $\widetilde{g}^{PH}_t(\xi)$ and $\widetilde{g}^{PP}_t(\xi)$ can be done in the same way. □

Proof of Theorem 4.7

***Step 1:* Eigenvalues of $\mathcal{E}_*(\xi)$** We prove firstly that the eigenvalues of $\mathcal{E}_*(\xi)$ have indeed negative real parts (and even uniformly in $\xi \in \mathbb{R}^d$).

For each $\xi \in \mathbb{R}^d$, the eigenvalues $\lambda_1(\xi), \lambda_2(\xi) \in \mathbb{C}$ of $\mathcal{E}_*(\xi)$ are equal to

$$\frac{1}{2}\Bigl(\mathrm{tr}\bigl(\mathcal{E}_*(\xi)\bigr) \pm \sqrt{\bigl(\mathrm{tr}(\mathcal{E}_*(\xi))\bigr)^2 - 4\det(\mathcal{E}_*(\xi))}\Bigr).$$

By (A.19), (A.20), there exist $\alpha, \beta > 0$ such that, for all $\xi \in \mathbb{R}^d$,

$$\begin{aligned}\mathrm{tr}\bigl(\mathcal{E}_*(\xi)\bigr) &= \mathtt{a}_*(\xi) + \mathtt{d}_*(\xi) \leq -\alpha, \\ \det\bigl(\mathcal{E}_*(\xi)\bigr) &= \mathtt{a}_*(\xi)\mathtt{d}_*(\xi) - \mathtt{b}_*(\xi)\mathtt{c}_* \geq \beta.\end{aligned}$$

We denote

$$z_*(\xi) := \bigl(\mathrm{tr}(\mathcal{E}_*(\xi))\bigr)^2 - 4\det(\mathcal{E}_*(\xi)) = \bigl(\mathtt{a}_*(\xi) - \mathtt{d}_*(\xi)\bigr)^2 + 4\mathtt{b}_*(\xi)\mathtt{c}_*.$$

Substituting the expressions from (4.17), one can easily rewrite

$$z_*(\xi) = \bigl(A^+ - \widetilde{J}^H_*(\xi) - q^H_*\bigl(B - \widetilde{b}(\xi)\bigr)\bigr)^2 - 4mq^P_*\widetilde{b}(\xi).$$

Therefore, if $z_*(\xi) < 0$, then the eigenvalues are not real, and

$$\mathrm{Re}\,\lambda_1(\xi) = \mathrm{Re}\,\lambda_2(\xi) \leq -\frac{\alpha}{2} < 0;$$

otherwise, if $z_*(\xi) \geq 0$ then the eigenvalues are real, and e.g.

$$\lambda_1(\xi) \leq \lambda_2(\xi) = \frac{1}{2}\Bigl(\mathrm{tr}\bigl(\mathcal{E}_*(\xi)\bigr) + \sqrt{\bigl(\mathrm{tr}(\mathcal{E}_*(\xi))\bigr)^2 - 4\det(\mathcal{E}_*(\xi))}\Bigr).$$

We have also, by (A.19), (3.16),

$$\operatorname{tr}\big(\mathcal{E}_*(\xi)\big) = \widetilde{J}^H_*(\xi) - A^+ + q^H_*\big(\widetilde{b}(\xi) - B\big) \geq -A^+ - q^H_* A^- - A^+ - 2Bq^H_* =: -\gamma.$$

For a fixed $d > 0$, function $f(x) := x + \sqrt{x^2 - 4d}$ is negative and decreasing on $[-\gamma, -\alpha]$, therefore, $f(x) \leq f(-\gamma)$. As a result,

$$\lambda_2(\xi) \leq \frac{1}{2}\Big(-\gamma + \sqrt{\gamma^2 - 4\det(\mathcal{E}_*(\xi))}\Big) \leq \frac{1}{2}\Big(-\gamma + \sqrt{\gamma^2 - 4\beta}\Big) < 0.$$

Hence, in both cases, there exists $\delta > 0$, such that

$$\operatorname{Re}\lambda_1(\xi) \leq \operatorname{Re}\lambda_2(\xi) \leq -\delta, \qquad \xi \in \mathbb{R}^d.$$

Step 2: **Convergence as** $\Delta t \to \infty$ By (A.26), for $t \geq 0$ and $\xi \in \mathbb{R}^d$,

$$\lim_{\Delta t \to \infty} \mathcal{F}_{t,\Delta t}(\xi) = \log \frac{q^H_*}{q^H_t} \begin{pmatrix} 1 & 0 \\ -1 & 0 \end{pmatrix} - \frac{1}{B} \log \frac{q^P_*}{q^P_t} \begin{pmatrix} \widetilde{a}^-(\xi) & \widetilde{b}(\xi) \\ A^- & -\widetilde{b}(\xi) \end{pmatrix}, \tag{A.37}$$

there exists $T > 0$ such that, for all $\Delta t > T$, the eigenvalues $\widehat{\lambda}_i = \widehat{\lambda}_i(t, \Delta t, \xi)$, $i = 1, 2$, of the matrix

$$\mathcal{E}_{\Delta t} := \frac{1}{\Delta t} M_{t,\Delta t} = \mathcal{E}_*(\xi) + \frac{1}{\Delta t}\mathcal{F}_{t,\Delta t}(\xi) \tag{A.38}$$

satisfy

$$\operatorname{Re}\widehat{\lambda}_1 \leq \operatorname{Re}\widehat{\lambda}_2 < -\delta < 0, \qquad \Delta t > T.$$

Let $P_{\Delta t}$ be the matrix constructed by eigenvectors of $\mathcal{E}_{\Delta t}$, so that

$$\mathcal{E}_{\Delta t} = P_{\Delta t}\mathcal{L}_{\Delta t}P^{-1}_{\Delta t}, \qquad \mathcal{L}_{\Delta t} := \begin{pmatrix} \widehat{\lambda}_1 & 0 \\ 0 & \widehat{\lambda}_2 \end{pmatrix}.$$

By (A.38) and (A.37),

$$\lim_{\Delta t \to \infty} P_{\Delta t} = P(\xi), \qquad \lim_{\Delta t \to \infty} P^{-1}_{\Delta t} = P(\xi)^{-1},$$

where $P(\xi)$ is the matrix constructed by eigenvectors of $\mathcal{E}_*(\xi)$.

Let $\|\cdot\|_2$ denote the Euclidean (a.k.a. spectral) matrix norm. Using the representation

$$e^{M_{t,\Delta t}(\xi)} = e^{\Delta t \cdot \mathcal{E}_{\Delta t}} = e^{P_{\Delta t}(\Delta t \cdot \mathcal{L}_{\Delta t})P^{-1}_{\Delta t}} = P_{\Delta t} e^{\Delta t \cdot \mathcal{L}_{\Delta t}} P^{-1}_{\Delta t},$$

with

$$e^{\Delta t \cdot \mathcal{L}_{\Delta t}} = \begin{pmatrix} e^{\Delta t \cdot \widehat{\lambda}_1} & 0 \\ 0 & e^{\Delta t \cdot \widehat{\lambda}_2} \end{pmatrix},$$

we may estimate, for large enough Δt,

$$\begin{aligned} \Big\| e^{M_{t,\Delta t}(\xi)} \Big\|_2 &\leq \big\|P_{\Delta t}\big\|_2 \big\|P^{-1}_{\Delta t}\big\|_2 e^{\Delta t \cdot \operatorname{Re}\widehat{\lambda}_2} \\ &\leq \Big(1 + \big\|P(\xi)\big\|_2 \big\|P(\xi)^{-1}\big\|_2\Big) e^{-\delta \Delta t} \to 0, \quad \Delta t \to \infty. \end{aligned}$$

Therefore both summand in (A.35) converges to zero-vectors pointwise as $\Delta t \to \infty$.

Step 3: **Convergence as $t \to \infty$, item (a)** By Proposition 4.1 and Remark A.2, there exists $c_1, c_2 > 0$, such that $0 < c_1 < q_t^A < c_2$, $A \in \{H, P\}$, for all $t > 0$, provided that $q_0^H > 0$, $q_0^P > 0$. Therefore, by (A.25), (A.26), (A.34), the entries of matrix $M_{t,\Delta t}(\xi)$ are uniformly bounded in both $t \geq 0$ and $\xi \in \mathbb{R}^d$. Next, by Theorem 4.4, $\widetilde{g}_t^{AB}(\xi)$ converges to $\widetilde{g}_t^{AB}(\xi)$, $A, B \in \{H, P\}$, in $L^1(\mathbb{R}^d)$. Clearly, for any function $|u_t(\xi)| \leq C$, $t \geq 0$, $\xi \in \mathbb{R}^d$, which converges to some $u_*(\xi)$ pointwise as $t \to \infty$, and for any $w_t \to w_*$, $t \to \infty$, in $L^1(\mathbb{R}^d)$, we have

$$\|u_t w_t - u_* w_*\|_{L^1(\mathbb{R}^d)} \leq C\|w_t - w_*\|_{L^1(\mathbb{R}^d)} + \|(u_t - u_*)w_*\|_{L^1(\mathbb{R}^d)} \to 0,$$

by the assumption and the dominated convergence theorem. Therefore, the following entry-wise limit, cf. (A.35), takes place in $L^1(\mathbb{R}^d)$:

$$e^{M_{t,\Delta t}(\xi)} \begin{pmatrix} \widetilde{g}_t^{HH}(\xi) \\ \widetilde{g}_t^{HP}(\xi) \end{pmatrix} \to e^{\Delta t \cdot \mathcal{E}_*(\xi)} \begin{pmatrix} \widetilde{g}_*^{HH}(\xi) \\ \widetilde{g}_*^{HP}(\xi) \end{pmatrix}, \qquad t \to \infty,$$

where we used that, by (A.26),

$$\lim_{t\to\infty} \mathcal{F}_{t,\Delta t}(\xi) = \mathbf{0}, \qquad \Delta t \geq 0, \qquad \xi \in \mathbb{R}^d, \tag{A.39}$$

where, recall, $\mathbf{0}$ denotes 2×2 matrix of zeros.

Next, by (A.36),

$$\begin{aligned}\left\|e^{M_{t,\Delta t}(\xi)} - e^{M_{t,\Delta t}(\infty)}\right\| &\leq e^{\max\{\|M_{t,\Delta t}(\xi)\|, \|M_{t,\Delta t}(\infty)\|\}} \\ &\times \left(\left\| \Delta t \cdot \begin{pmatrix} \widetilde{J}_*^H(\xi) & -q_*^H \widetilde{b}(\xi) \\ 0 & q_*^H \widetilde{b}(\xi) \end{pmatrix} \right\| + \left\| \frac{1}{B} \log \frac{q_{t+\Delta t}^P}{q_t^P} \begin{pmatrix} \widetilde{a}^-(\xi) & \widetilde{b}(\xi) \\ 0 & -\widetilde{b}(\xi) \end{pmatrix} \right\| \right),\end{aligned}$$

and the latter can be majorized uniformly in t by the norm of a matrix with integrable in ξ entries. As a result, the second summand in (A.35) converges to its limit in $L^1(\mathbb{R}^d)$ as well:

$$\left(e^{M_{t,\Delta t}(\xi)} - e^{M_{t,\Delta t}(\infty)}\right) \begin{pmatrix} q_t^H \\ 0 \end{pmatrix} \to \left(e^{\Delta t \cdot \mathcal{E}_*(\xi)} - e^{\Delta t \cdot \mathcal{E}_*(\infty)}\right) \begin{pmatrix} q_*^H \\ 0 \end{pmatrix}, \qquad t \to \infty.$$

As a result, the following convergence is in $L^1(X)$ (entrywise) as $t \to \infty$

$$\begin{pmatrix} \widetilde{g}_{t,\Delta t}^{HH}(\xi) \\ \widetilde{g}_{t,\Delta t}^{HP'}(\xi) \end{pmatrix} \to e^{\Delta t \cdot \mathcal{E}_*(\xi)} \begin{pmatrix} \widetilde{g}_*^{HH}(\xi) \\ \widetilde{g}_*^{HP}(\xi) \end{pmatrix} + \left(e^{\Delta t \cdot \mathcal{E}_*(\xi)} - e^{\Delta t \cdot \mathcal{E}_*(\infty)}\right) \begin{pmatrix} q_*^H \\ 0 \end{pmatrix}.$$

As a result, the inverse Fourier transforms converges uniformly in space. For the rest two functions, the proof is the same.

Step 4: **Convergence as $t \to \infty$, items (b)–(c)** The convergences in (4.36)–(4.37) follows immediately from the expressions (A.27)–(A.28) and the limit in (A.39). This, together with Step 1 above finishes the proof of item (c).

Next, by the above, for each $AB \in \{HH, HP', PH, PP\}$,

$$\widetilde{g}^{AB}_{t,\Delta t}(\xi) = h^{AB}_{t,\Delta t}(\xi) - h^{AB}_{\infty,t,\Delta t}(\infty).$$

By the proved in (4.36)–(4.37), one can pass here to the limit as $t \to \infty$. Then, by passing t to ∞ in (A.31)–(A.32), we immediately get the first three equalities in (4.34). Finally, by (A.33), for $A^+ \neq m$,

$$\begin{aligned} h^{AB}_{\infty,t,\Delta t}(\infty) &= \lim_{t\to\infty} \frac{Bq^P_* \Delta t - \log \frac{q^H_{t+\Delta t}}{q^H_t} - \frac{1}{B} \log \frac{q^P_{t+\Delta t}}{q^P_t}}{-(A^+ - m)\Delta t + \log \frac{q^H_{t+\Delta t}}{q^H_t}} \Bigl(q^H_{t+\Delta t}\, e^{-A^+\Delta t} - q^H_t\, e^{-m\Delta t} \Bigr) \\ &= \frac{Bq^P_* q^H_* \Delta t}{-(A^+ - m)\Delta t} \Bigl(e^{-A^+\Delta t} - e^{-m\Delta t} \Bigr) = \frac{mq^P_*}{m - A^+} \Bigl(e^{-A^+\Delta t} - e^{-m\Delta t} \Bigr); \end{aligned}$$

and for $A^+ = m$, also from (A.33), we have:

$$\begin{aligned} h^{AB}_{\infty,t,\Delta t}(\infty) &= \lim_{t\to\infty} \frac{Bq^P_* \Delta t - \log \frac{q^H_{t+\Delta t}}{q^H_t} - \frac{1}{B} \log \frac{q^P_{t+\Delta t}}{q^P_t}}{\log \frac{q^H_{t+\Delta t}}{q^H_t}} \bigl(q^H_{t+\Delta t} - q^H_t \bigr) e^{-A^+\Delta t} \\ &= \lim_{t\to\infty} \frac{Bq^P_* \Delta t - \frac{1}{B} \log \frac{q^P_{t+\Delta t}}{q^P_t}}{\log \frac{q^H_{t+\Delta t}}{q^H_t}} \left(\frac{q^H_{t+\Delta t}}{q^H_t} - 1 \right) q^H_t e^{-A^+\Delta t}, \end{aligned}$$

and since, by L'Hôpital's rule, $\lim\limits_{x\to 1} \frac{x-1}{\ln x} = \lim\limits_{x\to 1} \frac{1}{\frac{1}{x}} = 1$, we conclude that

$$h^{AB}_{\infty,t,\Delta t}(\infty) = Bq^P_* \Delta t q^H_* e^{-A^+\Delta t} = mq^P_* \Delta t\, e^{-A^+\Delta t}$$

for $A^+ = m$, that finishes the proof of (4.35). □

References

[1] S. Cornell, Y. Suprunenko, D. Finkelshtein, P. Somervuo, and O. Ovaskainen. A unified framework for analysis of individual-based models in ecology and beyond. *Nat. Commun.*, 10(1), 2019.

[2] S. Cornell, Y. Suprunenko, O. Ovaskainen, and P. Somervuo. Software for "A unified framework for analysis of individual-based models in ecology and beyond", Sep 2019.

[3] D. Finkelshtein. Extinction threshold in spatial stochastic logistic model: Space homogeneous case, 2020. Preprint, arXiv:2004.04830.

[4] L. Grafakos. *Classical Fourier analysis*, volume 249 of *Graduate Texts in Mathematics*. Springer, New York, third edition, 2014. ISBN 978-1-4939-1193-6; 978-1-4939-1194-3. xviii+638 pp.

[5] O. Ovaskainen, D. Finkelshtein, O. Kutoviy, S. Cornell, B. Bolker, and Y. Kondratiev. A mathematical framework for the analysis of spatial-temporal point processes. *Theor. Ecol.*, 7(1):101–113, 2014.
